# Genome-wide analyses of chromatin interactions after the loss of Pol I, Pol II, and Pol III

**DOI:** 10.1186/s13059-020-02067-3

**Published:** 2020-07-02

**Authors:** Yongpeng Jiang, Jie Huang, Kehuan Lun, Boyuan Li, Haonan Zheng, Yuanjun Li, Rong Zhou, Wenjia Duan, Chenlu Wang, Yuanqing Feng, Hong Yao, Cheng Li, Xiong Ji

**Affiliations:** 1 Key Laboratory of Cell Proliferation and Differentiation of the Ministry of Education, School of Life Sciences, Peking-Tsinghua Center for Life Sciences, Peking University, Beijing, 100871 China; 2 Academy for Advanced Interdisciplinary Studies, School of Life Sciences, Center for Statistical Science, Center for Bioinformatics, Peking University, Beijing, 100871 China

**Keywords:** RNA polymerases, 3D chromatin organization, Transcription

## Abstract

**Background:**

The relationship between transcription and the 3D chromatin structure is debated. Multiple studies have shown that transcription affects global Cohesin binding and 3D genome structures. However, several other studies have indicated that inhibited transcription does not alter chromatin conformations.

**Results:**

We provide the most comprehensive evidence to date to demonstrate that transcription plays a relatively modest role in organizing the local, small-scale chromatin structures in mammalian cells. We show degraded Pol I, Pol II, and Pol III proteins in mESCs cause few or no changes in large-scale 3D chromatin structures, selected RNA polymerases with a high abundance of binding sites or active promoter-associated interactions appear to be relatively more affected after the degradation, transcription inhibition alters local, small loop domains, as indicated by high-resolution chromatin interaction maps, and loops with bound Pol II but without Cohesin or CTCF are identified and found to be largely unchanged after transcription inhibition. Interestingly, Pol II depletion for a longer time significantly affects the chromatin accessibility and Cohesin occupancy, suggesting that RNA polymerases are capable of affecting the 3D genome indirectly. These direct and indirect effects explain the previous inconsistent findings on the influence of transcription inhibition on the 3D genome.

**Conclusions:**

We conclude that Pol I, Pol II, and Pol III loss alters local, small-scale chromatin interactions in mammalian cells, suggesting that the 3D chromatin structures are pre-established and relatively stable.

## Background

The relationship between transcription and 3D chromatin structures is one of the most fundamental questions in the postgenomic era [1–4]. Mounting evidence has shown that a high level of transcription activity is correlated with a higher frequency of DNA interactions in both development and diseases [5–8]. Topologically associating domain (TAD) boundaries, insulated neighborhoods, or CTCF loop domains are usually enriched with active transcription accompanied by both protein machineries and noncoding RNAs [9–11]. In *Drosophila melanogaster*, transcription predicts chromatin organization [12–14], suggesting a potential causal relationship between transcription and the 3D chromatin landscape.

Recent studies combining knockouts and inhibition showed that transcription could relocate Cohesin over mammalian chromatin [15], indicating that Pol II may regulate the 3D genome via its impact on Cohesin. Blocking of the transcription elongation enhances Cohesin binding and loop formation at CTCF-binding sites within the gene bodies in mammalian cells [16]. As the *D. melanogaster* genome has a much higher gene density than the mammalian genome, inhibiting *D. melanogaster* transcription significantly alters chromatin interactions both within and between domains, but has very little effect on the 3D topology of TADs [12–14]. Therefore, it is unclear whether Pol II regulates 3D chromatin landscapes via Cohesin directly.

The inhibition of Pol II transcription during the early development of mouse embryos did not affect TAD structures [17, 18], but the finding was difficult to interpret because of the relatively low sequencing depth used in these experiments and developmental arrest after transcription inhibition. The chromatin organization of transcriptionally inactive mature oocytes and sperm is quite similar to that of the embryonic stem cells [17, 19–21], implying that it might not be transcription activity per se, but proteins involved in the transcription process may contribute to 3D genome organization. It is also possible that transcription changes Cohesin occupancy on a mostly small, gene scale, which may not have a notable effect on the large-scale chromatin structures that can be detected with the Hi-C method used on a large scale.

An unchanged pattern after transcription inhibition in mammalian cells is usually based on the aggregate analyses of all chromatin loops [17, 18, 21–23]. As CTCF and Cohesin play a predominant role in the 3D chromatin landscape and because they occupy most of the loops in mammalian cells, it is difficult to evaluate the contribution of transcription on chromatin structures [24–26]. It is premature to conclude that transcription has no impact on chromatin interactions in mammals because one of the critical pieces of evidence is missing in the field and needs to be evaluated: the precise roles of Pol II on the 3D genome in the absence of CTCF and Cohesin.

Pol I, Pol II, and Pol III are three distinct DNA-dependent RNA polymerases that function together with thousands of transcriptional and chromatin regulators to synthesize rRNA, mRNA, and tRNA, respectively [9, 27–33]. RNA polymerases may involve in 3D chromatin organization directly by interacting with structural proteins or noncoding RNAs or indirectly through the downstream effects of transcription. Although the structure and function of RNA polymerases have been studied for 50 years [34], the roles of Pol I and Pol III in 3D chromatin organization, in particular, have been poorly investigated compared to Pol II.

Here, we specifically degraded the largest essential subunits of Pol I, Pol II, and Pol III in murine embryonic stem cells. Large-scale chromatin interactions remained unchanged after Pol II depletion, while small, specific regions were more affected. Chromatin organization re-forms during mitotic exit in the absence of Pol II, supporting the pre-established model of the 3D genome. Therefore, we identified loops with bound Pol II but not with Cohesin or CTCF, and with both our findings and public data sets from different laboratories, we found that they were largely unchanged after transcription was inhibited. Additionally, acute depletion of Pol I and Pol III also did not result in changes in the large-scale chromatin structures, but a fraction of local, small-scale chromatin interactions seemed to be more affected. These results collectively demonstrate that RNA polymerases play a role in organizing local, small-scale 3D chromatin landscapes in mammalian cells. Interestingly, longer-term depletion of Pol II (≥ 6 h) reduced chromatin accessibility and Cohesin occupancy over chromatin. We propose that immediate transcription inhibition does not affect large-scale 3D chromatin structures but that indirect effects of transcription inhibition do affect them.

## Results

### Acute degradation of Pol I, Pol II, and Pol III in the mESCs with auxin-inducible degron technology

**Fig. 1:**
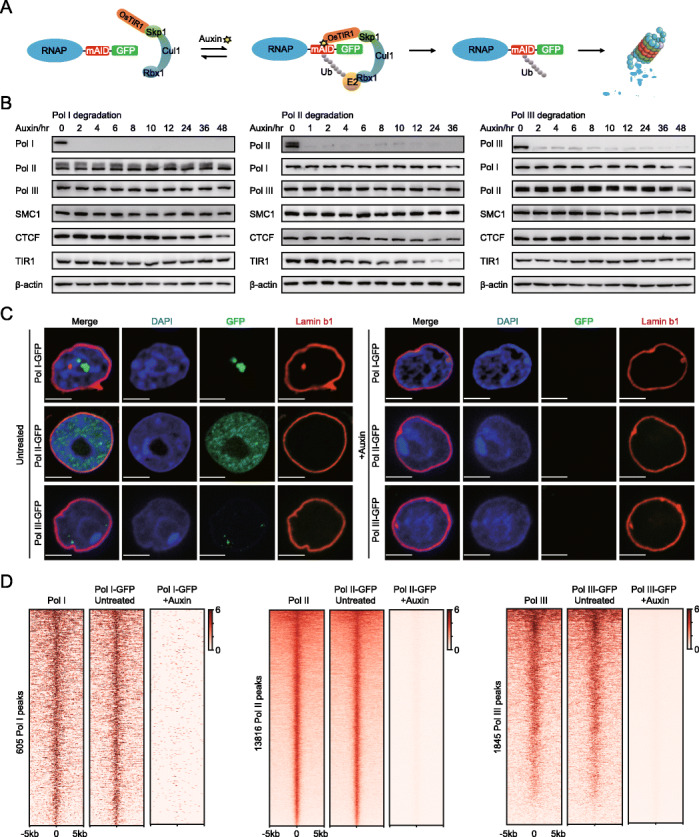
Rapid depletion of endogenous Pol I, Pol II, and Pol III proteins in the mESCs. **a** Schematic of auxin-inducible degron technology. Endogenous RNA polymerases are fused to the mAID-GFP tag in the C terminal with CRISPR genome editing. The fusion protein is recognized by OsTIR1 and subsequently degraded in the presence of auxin. Upon the removal of auxin, the Pol II protein was restored. **b** Western blot analyses of Pol I (RPA1), Pol II (RPB1), and Pol III (RPC1) protein levels after auxin treatment at different time points. Pol I (RPA1), Pol II (RPB1), Pol III (RPC1), Cohesin (SMC1), CTCF, and TIR1 (OsTIR1) protein levels were also examined. β-actin served as a loading control. **c** Lamin b1 immunofluorescence, DAPI, and GFP fluorescence signals for the RNA polymerases before and after a 6 h auxin treatment for Pol II and a 24 h auxin treatment for Pol I and Pol III. Images were obtained using a × 100 objective. **d** Left: Heatmap of the normalized ChIP-Seq signal centered at the Pol I peaks (*n* = 605) detected in the untreated cells, showing the marked reduction in Pol I binding in the degraded cells; the middle panel shows the Pol II peaks (*n* = 13,816); and the right panel shows the Pol III peaks (*n* = 1845). Heatmaps are ordered by descending ChIP-Seq signal intensity. RNA polymerases lost chromatin binding ability after auxin treatment (6 h for Pol II and 24 h for Pol I and Pol III)

To investigate the roles of RNA polymerase proteins in 3D chromatin organization, we subjected mESCs to RNA polymerase degradation with degron technology. RNA polymerase-mediated transcription can be inhibited with different inhibitors; however, they either do not have the capacity to distinguish different RNA polymerases or are specific to a single RNA polymerase (such as alpha-amanitin) and only work at high concentrations, thus requiring a long treatment duration [35, 36]. The auxin-inducible degron system was applied to RNA polymerase subunits because it degrades protein rapidly and specifically (Fig. 1a; Additional file 1: Fig. S1a) [37, 38]. This system enabled us to specifically investigate endogenous Pol I (Rpa1), Pol II (Rpb1), and Pol III (Rpc1) with a GFP tag and the perturbation effects of loss of function through the use of a mAID tag.

We confirmed that the RPA1, RPB1, and RPC1 proteins, as mAID-GFP fusion proteins, could be depleted in murine embryonic stem cells. To determine the time point for Pol II loss of function, we first induced OsTIR1 expression for 12 h, added auxin, and then performed western blot analyses at different time points. Indeed, rapid degradation can be achieved for a specific RNA polymerase after the addition of auxin (Fig. 1b, c). RNA polymerase ChIP-Seq data sets were generated after depletion. A ChIP-Seq heatmap analysis confirmed that protein depletion was efficient with the auxin-inducible degron technology (Fig. 1d). A Venn diagram shows the common and specific peaks among Pol I, Pol II, and Pol III, with each having a different peak size distribution (Additional file 1: Fig. S1d). Furthermore, the global level of mature mRNAs measured by polyA RNA-Seq was not changed dramatically within 6 h of Pol II depletion (Additional file 1: Fig. S1b; Additional file 2: Table S1). The RNA polymerase chromatin binding affinity, mRNA transcriptome, and 3D chromatin structures in the wild-type and tagged RNA polymerase cells are highly correlated (Fig. 1d; Additional file 1: Fig. S1c, S1e, S2d), indicating that we could use tagged cells (untreated) as controls for our downstream analyses.

To determine whether the rapid depletion of RNA polymerase causes pleiotropic effects on mESCs, we compared the cellular and molecular properties of the engineered and wild-type mESCs under our experimental conditions. The cell viability, cell cycle, caspase 3/7 activities, and γH2AX levels were comparable in the wild-type and the engineered cells upon treatment with doxycycline and auxin (Additional file 1: Fig. S1f-h, S1j). These engineered cells behaved similarly to the vehicle-treated wild-type cells, indicating that the rapid depletion of the RNA polymerases did not cause measurable effects in the cells at the time points when they were evaluated and that mAID-GFP tagging did not interfere with the physiological properties of mESCs (Additional file 1: Fig. S1f-j). A degradation time of 6 h for Pol II and 24 h for Pol I and Pol III was chosen for the downstream Hi-C analyses because these proteins are degraded quickly. The depletion did not cause noticeable changes in the protein level of other chromatin structural regulators (i.e., SMC1 and CTCF) or on the largest subunits of Pol I, Pol II, and Pol III (Fig. 1b), although a slight destabilization of endogenously tagged proteins was observed, as reported previously [37]. These results suggest that the mAID-GFP fusion supports the essential functions of RNA polymerases.

### A/B compartments and TAD structures were largely unchanged after acute depletion of Pol I, Pol II, and Pol III

Previous studies on the relationships between transcription and 3D genome usually focused on Pol II [3, 39–41]; here, we investigated Pol I, Pol II, and Pol III separately for their roles in the 3D genome. To generate high-resolution chromatin structure data after RNA polymerase depletion, we used our recently developed BAT Hi-C method (Bridge linker-Alul-Tn5 Hi-C) [42], which can efficiently delineate chromatin conformational features such as DNA loops (the “Materials and methods” section, Additional file 3). A combination of the BAT Hi-C technique and a RNA polymerase rapid degradation system enabled us to adequately investigate the relationships between specific RNA polymerases and chromatin structures.

Pol I, Pol II, and Pol III degron cells were subjected to degradation and then were collected for BAT Hi-C analyses. Two biological Hi-C replicates for both untreated cells (total reads = 277 million) and Pol II-degraded cells (total reads = 300 million) were obtained (Additional file 2: Table S2). The Hi-C data were reproducible (Additional file 1: Fig. S2a) and consistent with data in the mESC Hi-C data quality matrix and for the A/B compartments as published previously (Additional file 1: Fig. S2b-c) [43]. We pooled the data and acquired a 25-kb resolution Hi-C data set under both untreated and degraded Pol II conditions. The quality of the Hi-C data sets after Pol I or Pol III degradation was similar to that of the Pol II degron Hi-C data sets (data not shown). Since the method using Hi-C to detect structures is sensitive to sequencing depth, we sampled our data sets to the same sequencing depths for comparison (Additional file 2: Table S2). Indeed, the insulation score and compartment PC1 values were comparable for the untreated Pol I, Pol II, and Pol III Hi-C data sets, and the average contact frequencies in each data set were also similar across the genome (Additional file 1: Fig. S2d-e).

**Fig. 2:**
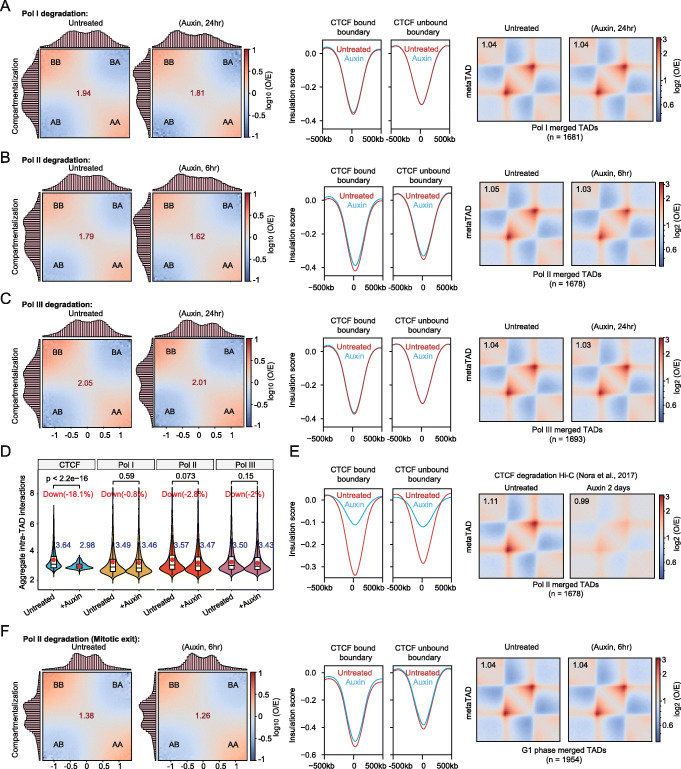
The compartments and TADs do not notably change after RNA polymerase depletion. **a** Saddle plots representing compartmentalization strength (left), average insulation score displayed for the CTCF-bound and CTCF-unbound TAD boundaries, from Bonev et al. [44] (middle); heat map of the average observed/expected Hi-C interactions in the TAD regions (right) under untreated and Pol I-degraded conditions (24 h). **b** Illustrated as **a**, but with Pol II degradation Hi-C data sets. **c** Illustrated as **a**, but with Pol III degradation Hi-C data sets. **d** Violin plots showing quantification of the aggregated intra-TAD observed/expected contact enrichment values for various RNAP-degraded conditions based on the BAT Hi-C data presented in **a**–**c**. Compared to that upon CTCF depletion, there was no significant change in these chromatin structures 6 h or 24 h after RNAP degradation. *p* values were calculated using a two-sided Wilcoxon test. **e** Averaged insulation score displayed for the CTCF-bound and CTCF-unbound TAD boundaries from Bonev et al. [44] (left), and heat map of the average observed/expected Hi-C interactions in the TAD regions (right) under untreated and CTCF-degraded conditions (2 days). **f** Illustrated as **a**, but with Pol II degradation during mitotic exit in Hi-C data sets

We next sought to investigate whether perturbations occurred in the A/B compartments or TADs upon the rapid depletion of each RNA polymerase in the mESCs. The A/B compartments were first delineated using Eigenvectors. Then, a Saddle plot displaying the compartmentalization strengths indicated that the degradation of the RNA polymerases did not cause apparent effects on the B-B, B-A, A-B, or A-A contact frequencies (Fig. 2a–c, left panel, Additional file 1: Fig. S2f). The TADs are identified by an arrowhead, and we identified 1589 TADs in the Hi-C data set of the untreated cells and 1496 TADs in the Hi-C data set of the cells with Pol II degradation (Additional file 2: Table S3). We then compared the Hi-C-detected TAD structures in cells under untreated and degraded conditions. The TAD boundaries identified in our Hi-C data set confirmed the TAD boundaries reported previously [43, 44]. The averaged chromatin interactions around TAD structures showed no noticeable changes (data not shown). The observed reads were resampled to equal amount divided by the expected reads (O/E) and used to calculate the averaged Hi-C signals around TAD regions. These O/E values of the interactions underestimated the differences but reduced the interference caused by experimental variations under the two conditions. Consistently, the observed/expected chromatin interactions for the TAD structures did not change notably after RNA polymerase degradation (Fig. 2a–c, right panel), and the relative chromatin interaction frequencies were almost the same for different distances (Additional file 1: Fig. S2g). For comparison, the Hi-C data sets for the CTCF degradation condition were reanalyzed with the same methods described above for the polymerases, and the results indicated that CTCF degradation caused a significant decrease in the number of intra-TAD interactions (Fig. 2d, e). We also performed insulation score analyses with the TAD boundaries defined through high-resolution Hi-C analyses published previously [44]. The results showed that there were no obvious changes in either the CTCF-bound or CTCF-unbound boundaries under the untreated and auxin conditions used for the Pol I, Pol II, or Pol III degron cells (Fig. 2a–c), while the insulation score showed dramatic decreases in the publicly available CTCF degradation Hi-C data sets (Fig. 2e) [24]. These results indicate that the TADs and compartments were largely unchanged after Pol I, Pol II, and Pol III depletion.

### Chromatin structures were re-established upon mitotic exit after Pol II depletion

Our evidence suggested that RNA polymerases have little or no impact on the maintenance of the large-scale 3D genome, but RNA polymerases might function during the process of its establishment. To explore this assumption, we performed Pol II degradation followed by chromatin structure analyses during mitotic exit. Previous studies reported that TAD structures disappear during mitosis and reappear in the early G1 phase [45]. We synchronized our cells into the M phase, simultaneously degraded Pol II, and then collected both the untreated and Pol II-degraded cells for Hi-C analyses upon mitotic exit. Following previously published protocols, we analyzed the cell cycle and found that approximately 90% of the cells were synchronized into M phase (Additional file 1: Fig. S2h) [46]. By comparing the cells with or without Pol II during mitotic exit, we found that the degradation of Pol II during mitotic exit did not cause obvious changes in the A/B compartments or TAD structures (Fig. 2f). These results revealed that the Pol II protein is nonessential for both the maintenance and establishment of large-scale chromatin structures in mESCs.

### Pol I, Pol II, and Pol III loss altered local, small-scale chromatin interactions

**Fig. 3:**
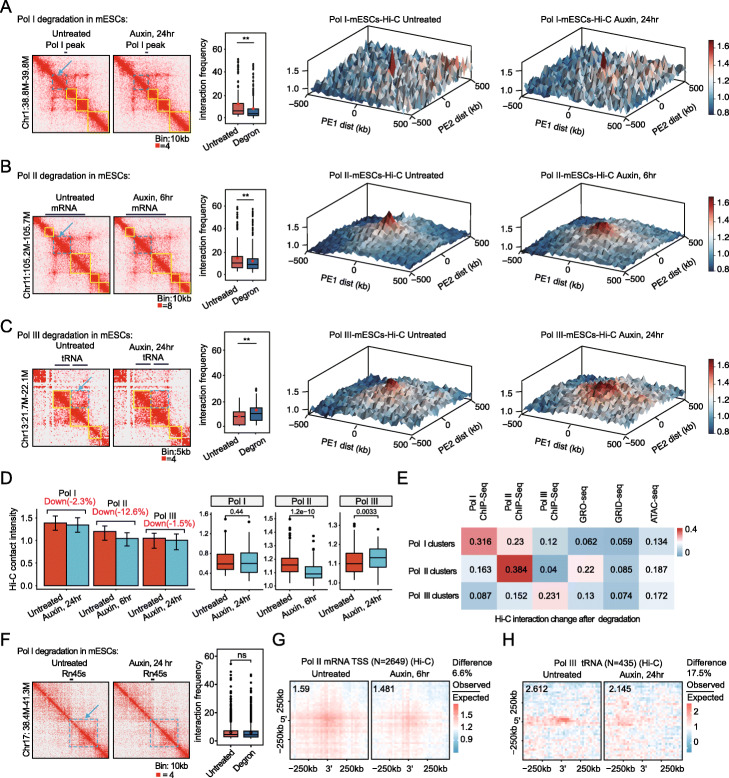
RNA polymerase depletion caused few or no changes in the local, small-scale chromatin interactions according to the Hi-C data sets. **a** Hi-C contact maps for the Pol I peak: 38.8–39.8-Mb region of chromosome 1. The yellow line marks regions of insulated domains, and the signals indicated by the blue dashed line mark regions were quantified as illustrated with the box plot in the left panel. Significance was determined using a Wilcoxon test (**p < 0.01). PE-SCAn analysis results (10-kb resolution) depicting the Hi-C contact frequency for the high-density clusters of Pol I under untreated and degraded conditions are displayed in the right panel. The area shown is centered on the respective RNAP-binding sites (including 500 kb upstream and downstream). The *y* axis indicates the Hi-C contact frequency. **b** Hi-C contact maps for the mRNA (*Tlk2*) loci: 105.2–105.7-Mb region of chromosome 11 at 10-kb resolution in the Pol II degron cell line under untreated (left) and auxin-treated (right) conditions with Pol II degron cell line. **b** is illustrated as **a** but based on the Pol II degradation Hi-C data sets. **c** Hi-C contact maps for the tRNA cluster: 21.7–22.1-Mb region of chromosome 13 at 10-kb resolution in the Pol III degron cell under untreated (left) and auxin-treated (right) conditions with Pol III degron cell line. **c** is illustrated as **a** but based on the Pol III degradation Hi-C data sets. **d** Bar graph shows the highest reproducible Hi-C contact intensity (means ± SEM) detected for Pol I-, Pol II-, or Pol III-binding clusters (red bars) and compared with the intensity of each in the degraded cells (blue bars) at each respective region, shown in the left panel. Box plot displaying the changes in the average contact frequency for the Pol I-, Pol II-, or Pol III-binding clusters after auxin treatment, shown in the right panel. For all the box plots, the centerline denotes the median; box limits denote the 25th–75th percentile. *p* values were calculated using a two-sided Wilcoxon test. **e** Correlation of Hi-C contact frequency changes in the Pol I clusters (top), Pol II clusters (middle), and Pol III clusters (bottom) after Pol I, Pol II, and Pol III depletion and Pol I, Pol II, and Pol III ChIP-Seq, GRO-Seq, GRID-Seq, and ATAC-Seq signals in the mESCs. **f** Normalized Hi-C contact maps for 45S rRNA (Chr17:38.4 M–41.3 M). The **f** is illustrated as **a**, but based on Pol I degradation Hi-C data sets. **g** PE-SCAn plots showing the aggregate Hi-C contact maps around pairs of active gene promoters (*N* = 2649) under untreated and Pol II-degraded conditions, respectively. The values of the central pixel relative to the bottom-left corner are annotated on the maps. **h** Same as **g**, but for tRNA (*N* = 435) under untreated and Pol III-degraded conditions

To test whether Pol I, Pol II, and Pol III organize local, small-scale chromatin structures, we first identified Pol I-, Pol II-, and Pol III-binding hotspots or clusters using the ROSE algorithm [47]. Overall, the Hi-C analysis showed that the contact frequency decreased 2.3%, 12.6%, and 1.5% in the binding clusters of Pol I, Pol II, and Pol III, respectively (Fig. 3d), and small, specific regions were more affected (Fig. 3a–c). Interestingly, Pol III-binding hotspots seemed to be more clustered after Pol III depletion (Fig. 3c), a finding in agreement with those on the previously known insulator functions of tRNA elements [48–50], and with a small but increased frequency of chromatin interactions across the tRNA clusters after Pol III depletion (Fig. 3c, d). Further correlation analyses of Hi-C interaction changes using different types of functional genomic data sets were performed, and we found that Hi-C interaction changes had a better correlation with the corresponding ChIP-Seq signals (Fig. 3e). The chromatin interactions of different genes that are known to be regulated by different RNA polymerases were also investigated. We found that the 45S rRNA locus did not obviously change after Pol I depletion, although relatively modest effects were found on the chromatin interactions in the mRNA and tRNA loci after the depletion of Pol II and Pol III (Fig. 3f–h), respectively. These results lead us to conclude that Pol I, Pol II, and Pol III play modest roles in structuring local, small-scale chromatin interactions, and Pol II seems to contribute more. Therefore, we focused on Pol II in the following study.

### Higher resolution chromatin interaction analyses indicated that Pol II contributes little to the organization of small loop domains

**Fig. 4:**
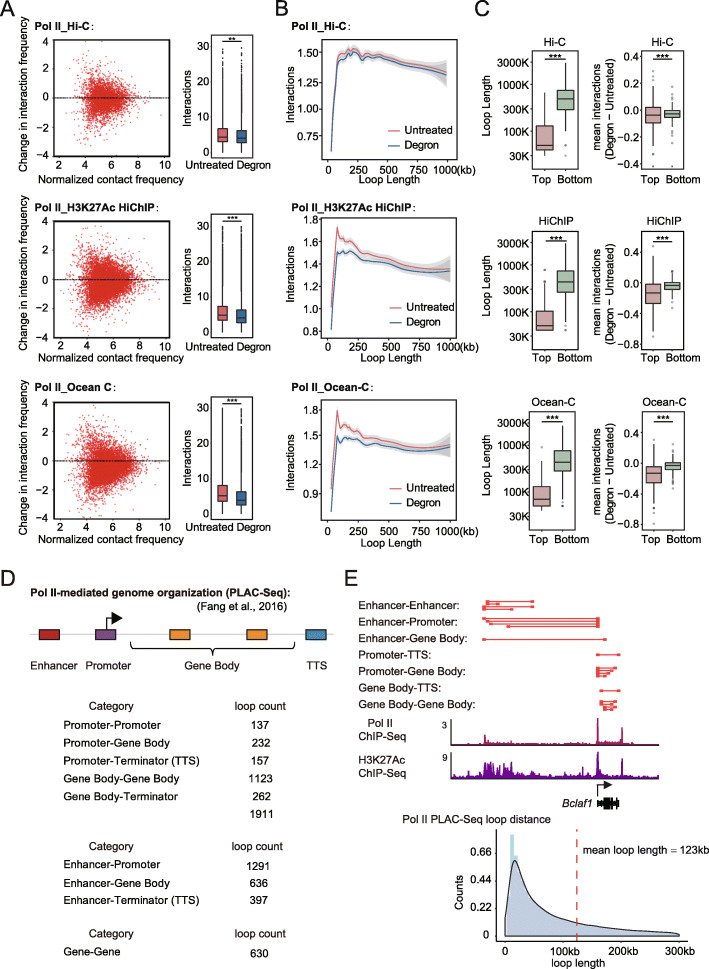
Higher resolution chromatin interaction analyses indicated that Pol II loss alters actively transcribed local, small loop domains. **a** Left: Scatter plot of the log fold change of contact frequency compared to the normalized mean contact frequency of the chromatin loops before and after Pol II degradation. Contact frequency was measured by Hi-C (top), H3K27ac HiChIP (middle), and Ocean-C (bottom). Right: Box plot of the contact frequency of the loops in the untreated and degron cells. Significance was calculated by Student’s *t* test (***< 0.001, **< 0.01). **b** Mean contact frequency ranked by the length of the chromatin loops under untreated and degron conditions using the same data sets as used in **a**. Data were smoothed by loess regression. **c** Chromatin loop length (left) and changes in mean contact frequency inside the loop domains (right) at high levels of transcription (top) and low levels of transcription (bottom), based on the same data sets as used in **a**. Significance was calculated using a Kolmogorov-Smirnov test (***< 0.001). **d** Global analyses of the Pol II PLAC-Seq-identified chromatin loops at gene regulatory elements (enhancers, promoters, gene bodies, and terminators (TTS)) in wild-type mESCs [56]. The category is listed in the left panel, and the loop counts for each category are listed in the right panel. Contact enrichment (obs/exp) for promoter-promoter (average PET number 11.16, hypergeometric *p* value 3.563683e−04) and promoter-enhancer loops (average PET number 15.28, and hypergeometric *p* value 6.477318e−06) were quantified. **e** The top panel shows Pol II-mediated chromatin loops (red lines). ChIP-Seq profiles for Pol II and H3K27ac are shown for the *Bclaf1* locus (middle). A schematic of the Pol II-mediated 3D chromatin structure at the *Bclaf1* locus is shown in the right panel. The histogram of the loop counts ranked by the length of the loops is shown at the bottom

The ability to determine the prevalent mechanisms underlying chromatin folding appear to highly depend on the resolution of the chromatin structure analyses. To obtain higher resolution information on the Pol II-dependent intra-TAD structures, we performed H3K27ac HiChIP and Ocean-C analyses after Pol II depletion for 6 h. The H3K27ac HiChIP data are used to map chromatin loops associated with active promoters and enhancers [51, 52], and Ocean-C, a recently developed antibody-free chromosome conformation capture technique, combines FAIRE-Seq with Hi-C to map hubs of open chromatin interactions [53]. Chromatin loops were identified by hichipper and HiCCUPS [54, 55] (Additional file 2: Table S4). The histogram shows that most of the loop interaction strength seems to be affected after Pol II degradation for the loops identified by both algorithms with the Hi-C, HiChIP, and Ocean-C data sets (Additional file 1: Fig. S3). The scatter plot and box plot of the HiCCUPS-identified chromatin loops in all three data sets consistently show a slight decrease after Pol II depletion (Fig. 4a).

We next sought to identify the features of the chromatin loops that were sensitive to Pol II depletion. Previous studies reported that active transcription defines the small compartmental domains throughout Eukarya, but that transcription itself does not predict 3D chromatin organization in mammals at specific loci [13]. Our analyses of chromatin structures after Pol II degradation were used to explore the existence of Pol II transcription-dependent loop domains. A loop-length analysis indicated that Pol II degradation mostly affected the interaction strength of the small loop domains (Fig. 4b), usually less than 250 kb, which appears to be similar to the small compartmental domains in *Drosophila* [13]. Considering that the current resolution of the Hi-C method is not sufficient to make a conclusion, we analyzed two other higher resolution data sets, HiChIP and Ocean-C, and both of them exhibited trends consistent with those shown by the Hi-C data. The HiChIP and Ocean-C data sets showed more significant changes, 14% and 15%, respectively, than were indicated by the Hi-C data set (4%) because they preferentially capture interactions involving open chromatin, and at a similar sequencing depth, they provide higher resolution than Hi-C.

If Pol II was involved in structuring small chromatin loops, we would anticipate the active and silent regions involved with Pol II transcription to behave differently after Pol II depletion. To test this idea, we ranked the average GRO-Seq (nascent transcript) signals for the loop domains and those of their upstream and downstream 100-kb windows, in decreasing order. Indeed, the top GRO-Seq signal-associated loop domains were smaller and showed a more significant decrease in the frequency of chromatin interactions than the bottom GRO-Seq signal-associated domains in the three different data sets (Fig. 4c). These results indicate that, although Pol II has no effect on large-scale genome structures, it contributes to actively transcribed local, small loops.

If Pol II contributes to local chromatin organization, then proximity ligation-assisted ChIP-seq (PLAC-Seq) analyses for Pol II would be expected to show that Pol II is preferentially associated with short-range chromatin loops [56]. We reanalyzed previously published Pol II PLAC-Seq in mESCs. The results showed that Pol II-associated interactions connected promoters, gene bodies, terminators, and enhancers (Fig. 4d; Additional file 2: Table S5). Most of these interactions were within 100 kb (Fig. 4e) and constrained within CTCF loop domains [57, 58]. Some Pol II-associated interactions involved chromatin clusters among genes and their potential regulatory elements, as observed for the *Bclaf1* locus (Fig. 4e). These results indicate that Pol II mediates chromatin loops within a short range around gene regulatory elements.

### Loops with bound Pol II but without Cohesin or CTCF were largely unchanged after Pol II depletion

Our RNA polymerase degron system degraded Pol II within 1 h (Fig. 1b), which provided the unique ability to separate the impact of transcription states (including other transcription apparatuses and active transcription-associated chromatin environments) from the presence of RNA polymerase proteins per se. Therefore, we performed BAT Hi-C analyses in mESCs 0 h, 1 h, and 6 h after Pol II depletion. These Hi-C data sets were highly reproducible, as we showed previously (Additional file 1: Fig. S2a, S4a). The compartment and TAD structure analyses reproducibly showed no obvious differences among 0 h, 1 h, and 6 h after Pol II depletion (Fig. 2b, f; Additional file 1: Fig. S4b-e). Taken together, our results demonstrate that Pol II proteins are dispensable for large-scale chromatin structures in mESCs.

**Fig. 5:**
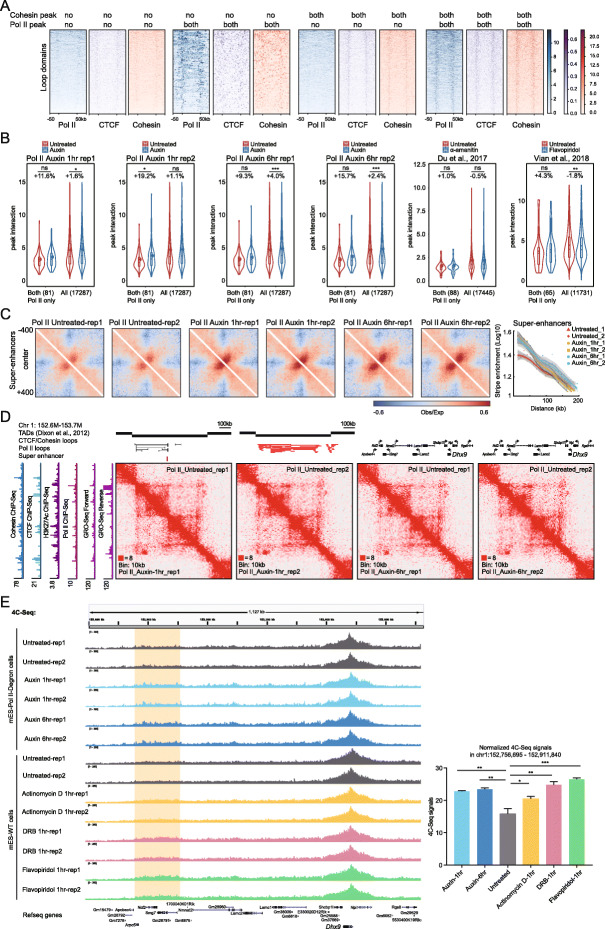
Loops with bound Pol II but without CTCF or Cohesin were largely unchanged after transcription inhibition. **a** Heatmap of the ChIP-Seq signals of loops classified by the extent of their overlap with Cohesin or Pol II peaks. “Both” means that both of loop anchors overlap with ChIP-Seq peaks, and “no” means that no anchor overlaps with a ChIP-Seq peak. The number of loops was determined based on the H3K27ac HiChIP data sets. The numbers in each group are 773, 101, 2649, and 787. **b** Violin plots of the loop peak interaction according to the Hi-C data under untreated and auxin(/inhibitors) conditions. Loops of Pol II bound at both anchors without Cohesin at either anchor were selected out and plotted with all loops included. The number of loops is indicated in parentheses. The dot inside the box plot denotes the mean interaction, and the percentage of change was calculated as the difference in the mean value divided by the untreated mean value. Significance was calculated by Student’s *t* test (***< 0.001, **< 0.01, *0.05). **c** Aggregate target-centered Hi-C maps reveal that Pol II degradation slightly increased of super-enhancer (SE) stripe formation. Pile-up maps were plotted with the 10-kb resolution contact matrix and normalized to the distance, with a positive signal in red, and a negative signal in blue. In the right panel, the signal decay curve of the SE stripes. The *x*-axis shows the enrichment of the SE stripes, and the *y*-axis shows the distance to the target center, up to 200 kb. **d** Hi-C contact maps for *Dhx9*: 152.6–153.7-Mb region of chromosome 1 at 10-kb resolution in the untreated and auxin-treated Pol II degron cell lines. The Cohesin, CTCF, H3K27ac ChIP-Seq signals, and GRO-Seq signals are displayed on the left. The black bars indicate TAD structures. The black lines indicate CTCF/Cohesin loops, and the red lines indicate Pol II-associated loops. **e** The 4C analyses at the *Dhx9* locus after Pol II degradation and transcription inhibition are shown. Transcription was inhibited with flavopiridol, actinomycin D, or DRB treatment for 1 h. The orange background regions indicate the 4C-enriched region, and the quantitative analysis (mean with SD) is shown on the right. The untreated signals indicate the average signals under untreated conditions for mES-WT and mES-Pol II degron cells. Significance was calculated using Student’s *t* test

Previous transcription inhibition followed by chromatin structure analyses also exhibited no changes, as determined through aggregated analyses [17, 18, 22]; these analyses did not exclude the possibility that subsets of chromatin loops might change after transcription inhibition. On the other hand, most of the chromatin loops in mammals are occupied by CTCF and Cohesin; therefore, it was better to select loops with bound Pol II but without Cohesin or CTCF to evaluate the contributions of Pol II in 3D genome organization. We found that the loops with bound Pol II but without Cohesin showed very little CTCF binding (Fig. 5a). A similar classification was applied to our Pol II degron Hi-C data sets, and these loops were largely preserved 1 h and 6 h after Pol II degradation (Fig. 5b). Then, we identified loops with bound Pol II but without Cohesin or CTCF and found that they were also preserved after Pol II depletion (Additional file 1: Fig. S5a). We then performed the same analyses with the previously obtained transcription inhibition Hi-C data sets on early embryo development and activated B cells [18, 22]. The analyses consistently showed that loops predominantly bound by Pol II were largely unchanged after transcription inhibition (Fig. 5b; Additional file 1: Fig. S5a). In contrast, CTCF-bound loops had a significantly decreased interaction strength after CTCF depletion, as determined using the same analysis pipeline (Additional file 1: Fig. S5b).

Super-enhancer loci are usually associated with extremely high levels of transcription [59, 60]. Therefore, we examined these regions for changes in chromatin interactions. The target-centered maps indicated that the super-enhancer regions had mildly enhanced chromatin interactions both 1 h and 6 h after Pol II degradation (Fig. 5c). Specifically, the interactions around the key pluripotency gene *Esrrb* and housekeeping gene *Dhx9* adjacent to super-enhancer-associated loop domains were also enhanced (Fig. 5d; Additional file 1: Fig. S5c). Moreover, this observation was independently validated by 4C-Seq analyses after Pol II depletion (Fig. 5e). We then inhibited transcription with actinomycin D, DRB, or flavopiridol and observed a consistent increasing trend in interaction frequencies (Fig. 5e). Recent live-cell imaging analyses indicated that Pol II transcription restrains the dynamics of chromatin [61]. This finding explains our observation that Pol II degradation caused a slight increase in the frequency of chromatin interactions at super-enhancer regions. These results further suggest that Pol II is not directly involved in large-scale chromatin organization but restrains the dynamics of chromatin associated with active genes, underlying a potential mechanism for the increase in the frequency of chromatin interactions after Pol II depletion.

### Pol II depletion altered the subset of promoter-associated chromatin interactions

**Fig. 6:**
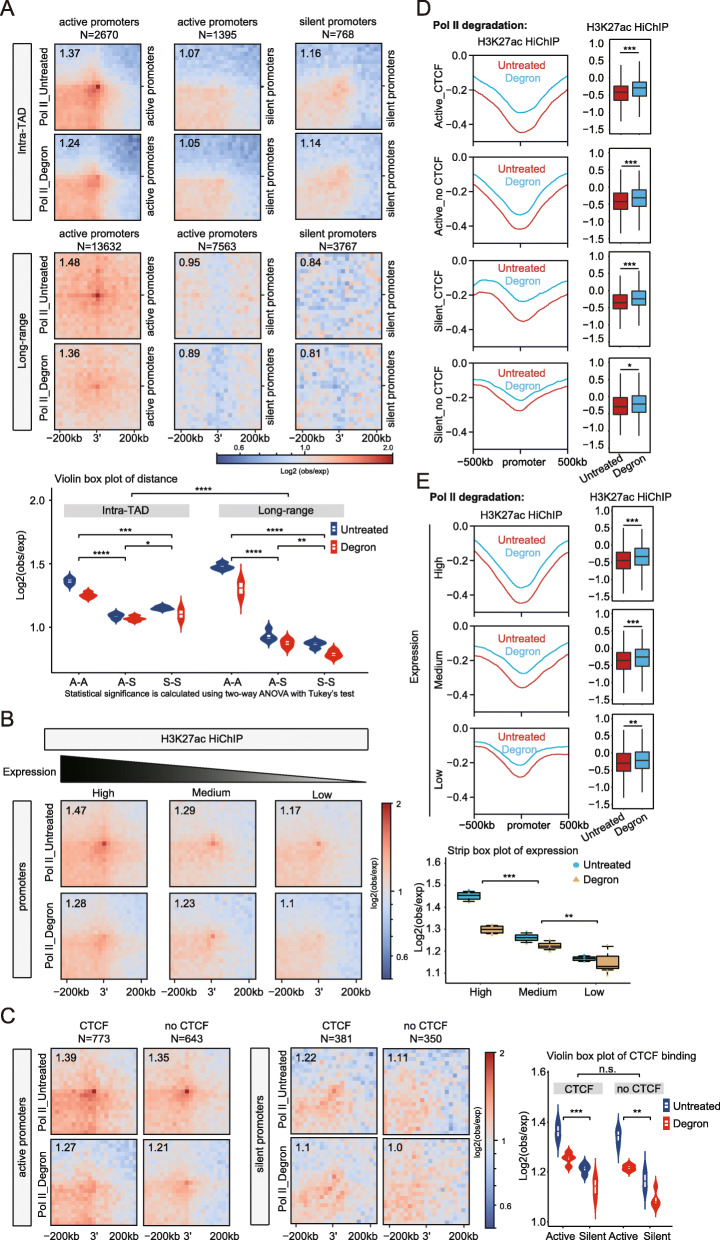
Pol II depletion had a relatively modest effect on the promoter-associated chromatin interactions. **a** Aggregate H3K27ac HiChIP contact maps around pairs of either active or silent gene promoters in the mESCs grouped by intra-TAD pairs (between 200 kb and 2 Mb) or cis long-range (from 2 to 10 Mb) (upper panel). Data are represented in a violin box plot showing the means ± SD (bottom panel). Significance was calculated using two-way ANOVA with Tukey’s test. **b** Aggregate H3K27ac HiChIP contact maps around pairs of active gene promoters in the mESCs and separated into three equal slices (each *N* = 833) based on the expression level (left panel). Data are represented as in a stripe box plot showing the means ± SD (right panel). Significance was calculated using two-way ANOVA with Tukey’s test. **c** Aggregated H3K27ac HiChIP contact maps around pairs of either active or silent gene promoters in the mESCs, which were classified based on the presence of CTCF-binding sites within ± 5 kb (left panel). Data are represented in a violin box plot showing the means ± SD (right panel). Significance was calculated using two-way ANOVA with Tukey’s test. **d** Average insulation score based on the H3K27ac HiChIP data set for promoters under untreated and Pol II-degraded conditions (6 h) (left panel), and the insulation score for promoters were quantified and are illustrated in the box plot in the right panel. Significance was determined using a Wilcoxon test (*p < 0.05, **p < 0.01, ***p < 0.001). **e** Illustrated as **d**, but the promoters were classified according to the gene expression level (high/medium/low)

Multiple studies have shown that active promoters engage in interactions over a long range [44, 58, 62, 63], but the roles of Pol II proteins in these promoter pairs remain unclear. We performed paired-end spatial chromatin analysis (PE-SCAn) among different subgroups with our high-resolution H3K27ac HiChIP and Ocean-C data sets on interactions after Pol II degradation. The PE-SCAn plots of pairs of promoters across multiple distances (intra-TAD < 2 MB, long-range > 2 MB) (Fig. 6a; Additional file 1: Fig. S6a) for a different expression levels (high/medium/low) (Fig. 6b; Additional file 1: Fig. S6b), with CTCF binding and without CTCF binding (Fig. 6c; Additional file 1: Fig. S6c), collectively showed that the distance and expression level modestly affected the active transcription-mediated promoter-promoter interactions in a CTCF-independent manner.

To further investigate the roles of Pol II in promoter-associated interactions, we performed insulation score analyses for different groups of promoters. We observed relatively few or no changes in the insulation scores of the active_CTCF-bound, active_no CTCF, silent_CTCF-bound, and silent_no CTCF promoters in the Pol II depletion H3K27ac HiChIP and Ocean-C data sets (Fig. 6d; Additional file 1: Fig. S6d). We then created high/medium/low subgroups of interactions associated with no CTCF promoters based on gene expression with GRO-Seq data. The analyses also showed that there were no or few differences in the changes in the insulation scores under untreated or Pol II degron conditions (Fig. 6e; Additional file 1: Fig. S6e).

Loops between promoters and the gene bodies of highly transcribed genes indicated stripe signals in C-types of data on chromatin structures [64, 65]. We performed stripe analyses with our H3K27ac HiChIP data sets at enhancer, promoter, and CTCF insulator regions. The results showed that there were relatively few or no differences in these stripe signals (Additional file 1: Fig. S6f), which is less than the changes with acute transcription inhibition with inhibitors [64]. The reasons for that could be (1) these interactions could be dynamic, which can only be robustly detected with the high-resolution Micro-C; or (2) our Pol II degradation inhibits gene transcription, but the Pol II depletion may not be complete in specific gene loci as recently reported in a different cell line [66]; or (3) these interactions are specific and setup by the transcription factors etc. that are present, and not by the general transcriptional process [67, 68].

### Immediate depletion of Pol II did not perturb Cohesin-chromatin binding, but prolonged depletion had an effect

**Fig. 7:**
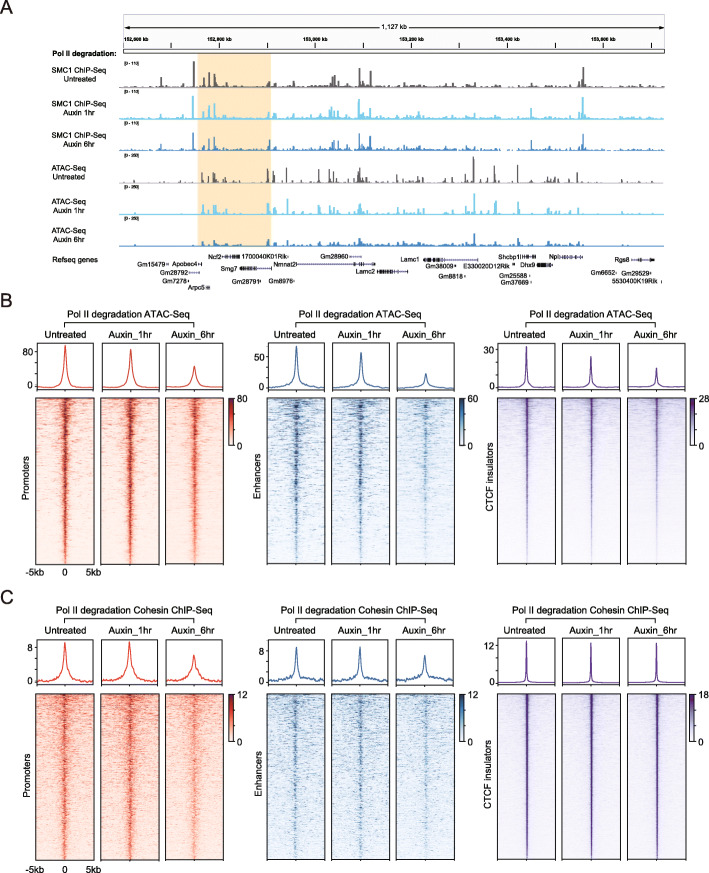
Pol II depletion for a long time significantly affected chromatin accessibility and Cohesin occupancy. **a** ChIP-Seq profiles for Cohesin (SMC1) and ATAC-Seq signals at the *Dhx9* locus 1 h and 6 h after Pol II degradation are shown. The orange background regions correspond to the 4C-Seq-enriched region in Fig. 5e. **b** Heatmap illustrating ATAC-Seq signals centered at all promoters, enhancers, and CTCF-binding insulators (± 5 kb) before (untreated) and after auxin treatment (1 h or 6 h). These regions were ranked by their level of chromatin accessibility (from high to low) for each category in the mESCs. **c** Same analysis as in **b** but focused on Cohesin ChIP-Seq binding

If Pol II is indeed nonessential in global 3D genome organization, how could transcription-mediated Cohesin-chromatin binding and transcription elongation-mediated chromatin structures be explained? We speculated that Pol II might indirectly regulate Cohesin. Taking advantage of the Pol II degron system, we degraded Pol II at different time points, especially at the 1 h time point (Fig. 1b). The single locus (Fig. 7a) and meta-gene analyses (Fig. 7b, c) of the ATAC-Seq signals and Cohesin (Smc1) ChIP-Seq data showed that these signals did not change 1 h after Pol II degradation but decreased after 6 h. The ATAC-Seq peaks bound with or without CTCF/Cohesin in both promoters and distal elements showed no obvious differences (Additional file 1: Fig. S7a), indicating general chromatin compaction after 6 h of Pol II depletion. These results implicate that Pol II degradation may indirectly change the 3D chromatin interactions. By inhibiting transcription for extended periods, many potential secondary effects may emerge (a decrease in the number of short half-life transcripts and noncoding RNAs, reduced chromatin accessibility, increased stress caused by transcription inhibition, etc.). Our data support a model in which transcription inhibition does not cause immediate changes in large-scale chromatin interactions but decreases the frequency of chromatin interactions at Pol II clusters, potentially by reducing chromatin accessibility and inhibiting Cohesin binding on chromatin.

## Discussion

The relationship between transcription and 3D genome organization is one of the most fundamental unresolved issues in modern molecular biology. Here, we provide the following evidence: (1) the specific transcription inhibition by the degradation of Pol I, Pol II, and Pol III results in few or no changes to large-scale 3D chromatin structures, as assayed by Hi-C (Fig. 2a–c, f); (2) select highly abundant Pol I-, Pol II-, and Pol III-binding sites, or active promoter-associated chromatin interactions were identified and seem to be affected to a relatively greater extent after degradation (Figs. 3a–c, f, g, and 6a–e); (3) high-resolution chromatin interaction maps were generated to reveal that transcription inhibition alters local, small loop domains (Fig. 4b, c); (4) the secondary effects and the immediate effects of transcription inhibition were potentially separated by the degradation of Pol II within 1 h, indicating that Pol II transcription is dispensable for large-scale chromatin organization (Additional file 1: Fig. S4b-d); and (5) Pol II dominant loops were identified in the absence of CTCF and Cohesin and were found to be largely unchanged after transcription inhibition (Fig. 5b; Additional file 1: Fig. S5a). These pieces of evidence collectively demonstrate that RNA polymerases play a relatively modest role in organizing local, small-scale genome organization. In addition, time course experiments showed that more prolonged polymerase depletion caused a decrease in Cohesin binding, but the short-term treatment did not have this effect (Fig. 7b, c), suggesting that RNA polymerases regulate the 3D chromatin landscape indirectly. RNA polymerases are core enzymes for transcription that are constitutively expressed in all cell types; therefore, 3D chromatin organization that is independent of transcription is likely the general case in mammalian cells. Furthermore, the Pol II degradation time course analyses also indicated that the small-scale effects of Pol II on chromatin structures could be dynamic and required for the maintenance of the proper, most efficient compartmentalization of chromatin substructures (such as placing enhancers nearer to target promoters).

Mounting evidence has shown a strong correlation between transcription and 3D genome structures [12, 44, 69], but this relationship was controversial prior to our study [17, 18]. Transcription inhibition experiments suggested the persistence of large-scale chromatin structures after transcription inhibition [17, 18], but they were based solely on aggregation analyses or based on a single resolution chromatin interaction map. Therefore, these studies could not exclude the possibility that transcription might play a predominant role in organizing specific types of chromatin structures or might have been identified through the higher resolution interaction analyses. Based on the literatures that we know, no previous studies thoroughly investigated the roles of transcription on 3D chromatin interactions to the extent that we did in this study. Our conclusion suggesting that transcription inhibition causes no or few effects on large-scale 3D chromatin structures is consistent with that of many previous studies and is essential for the field. On the other hand, many studies have shown that transcription inhibition affects 3D genome structures and Cohesin [15, 16], which seems to conflict with our conclusion. Our time course experiments clearly showed that prolonged depletion of Pol II reduces chromatin accessibility and inhibits Cohesin binding but that the shorter duration of Pol II degradation did not lead to these effects, suggesting that transcription regulates 3D genome structures indirectly. Our study perfectly explains the previously confusing results: transcription inhibition usually does not affect large-scale 3D genome structures, but the high doses, long-term treatment, or more sensitive biological systems may lead to chromatin structure changes by indirectly affecting Cohesin.

There are many genome architectural proteins (such as CTCF, YY1, Znf143, RNA polymerases, and RNA binding proteins and mediators) [24, 25, 62, 68, 70–74], and noncoding RNAs have been implicated in organizing 3D genome structures; that is, the 3D genome is likely organized via a combination of many factors. In this study, we showed that the Pol I-, Pol II-, and Pol III-binding and interaction hotspots and loops bound only by Pol II are largely unchanged after transcription inhibition. These results argue against a model of combinatory protein factors, but we cannot exclude the possibility that nuclear noncoding RNAs may organize 3D genome structures, which could be independent of active transcription. Additionally, Bonev et al., Cell 2017, showed that transcriptional activation at the endogenous loci does not lead to local changes in 3D genome structures [44], arguing that transcription alone is not sufficient to alter 3D genome topology.

We showed that transcription may indirectly affect 3D genome structures via Cohesin-chromatin binding, and there are many other possibilities. For example, a previous imaging study showed that Pol II transcription constrains the dynamics of chromatin in the nucleus [61]. After transcription inhibition, the highly transcribed regions move faster in the nucleus, interact frequently with specific regions, and interact less frequently with the other regions. Since transcription is vital for all genes, it is also likely that transcription induces the short half-life of chromatin structural proteins.

Previous studies showed that structural factors such as CTCF- and Cohesin-mediated chromatin interactions create frameworks for loop domains and constrain the high-frequency chromatin interactions within them [2, 3, 75, 76]. CTCF and Cohesin depletion destroys most chromatin structures in mammalian cells, but it has little effect on transcription [24, 26]. Here, we depleted the core subunits of Pol I, Pol II, and Pol III in mESCs and found few or no changes for either the large-scale or small-scale chromatin structures. These data suggest that transcription and 3D chromatin structures are largely uncoupled in the nucleus of mESCs. Previous signaling-induced and heat shock-following chromatin structure analyses suggest a pre-existing model for the 3D genome [77–79]. Our evidence also supports a pre-existing model, for which the mechanisms may be connected to genome sequences or epigenetic modifications.

## Conclusions

Our study provides the first comprehensive analyses of the roles of Pol I, Pol II, and Pol III proteins in 3D chromatin organization in mESCs. We demonstrate that RNA polymerases play a relatively modest role in organizing local, small-scale 3D chromatin structures, and we propose that transcription does not regulate the large-scale 3D genome directly but is able to regulate it indirectly. Our study explains the confusing findings on transcription inhibition on the 3D genome, which were the result of difficulties in separating the direct and indirect effects of transcription inhibition. Our study also implies that transcription and 3D chromatin organization are largely uncoupled in the nucleus. Since transcription is not essential for the 3D genome, further studies of genetics, epigenetic features of the 3D genome, and noncoding RNAs may reveal new insights into the 3D genome organization.

## Materials and methods

For more details, see Additional file 3.

### Mouse ES cell culture

The V6.5 mouse ES (mES) cell line, derived from the inner cell mass (ICM) of C57BL/6 × 129/sv crossed mice, was a gift from R. Young of the Whitehead Institute. These mES cells were cultured with irradiated mouse embryonic fibroblasts in DMEM-KO containing 15% FBS, leukemia inhibiting factor (LIF), l-glutamine, β-mercaptoethanol, nonessential amino acids, and penicillin/streptomycin at 37 °C with 5% CO_2_. These cells were harvested for downstream experiments after two passages of MEF feeders, as previously described [80]. These cells were tested and found to be free of mycoplasma contamination. For experiments, all degron mES cells were pretreated with 1 μg/ml doxycycline for 12 h. Pol II degron mES cells were treated with or without 500 μM indole-3-acetic acid (auxin/IAA) for 6 h, whereas Pol I and Pol III degron mES cells were treated for 24 h with the same concentration. To generate a time course Pol II degron depletion model, auxin was added at 1 h and 6 h. For wild-type mES cells treated with transcription inhibitors, actinomycin D and flavopiridol were added at 1 μM each and incubated for 1 h, DRB at 100 μM was added and incubated for 1 h, and etoposide was added at 10 μM and incubated for 1 h.

### Gene targeting

For transfection, plasmids were prepared using a HiPure Plasmid EF Mini Kit (Magen, P1112-02) and not linearized before transfection. For parental degron cell line construction, a Tir1-expressing cassette and Rosa26-targeting sgRNA were transfected into the V6.5 wild-type mESC line in equimolar amounts using FuGENE HD (Promega) following the manufacturer’s protocol. After 2 days, the cells were passaged and grown for 1 week in the presence of 5 μg/ml puromycin until single colonies could be collected. Clonal lines were assessed for their ability to express Tir1 after induction with 1 μg/ml tetracycline. To insert the mAID-EGFP cassette into the C-terminus of the Rpa1/Rpb1/Rpc1 subunits, a Tir1 stable-expressing clonal cell line was transfected with an mAID-EGFP targeting vector and sgRNAs. Neomycin was added to the medium at 100 μg/ml, and homozygous clonal lines were selected after genotyping. These clonal lines were assessed for their ability to undergo auxin-inducible degradation of each RNAP-mAID-eGFP and to show expression levels similar to that of RNA polymerases in wild-type mES cells. The clones degraded with the maximum efficiency were chosen for the following assays.

### BAT Hi-C

Hi-C was conducted as BAT Hi-C [42].

#### Bridge linker preparation and cell fixation

The bridge linker used for BAT Hi-C was synthesized from the forward strand, 5′-/5Phos/CGC GAT ATC/iBiodT/TA TCT GAC T-3′, and reverse strand, 5′-/5Phos/GTCAGATAAGATATCGCGT-3′. For linker preparation, we dissolved two reverse complementary single-strand linkers in 1 × TNE annealing buffer at a concentration of 100 μM, and a dTTP overhang was maintained at the 3′ ends of both strands, and then, an annealing mixture was prepared at an optimized F/R molecular ratio (normally 1:1), heated to boiling and cooled slowly to room temperature. Cells were dissociated and resuspended in freshly made 1% formaldehyde (methanol free) at a volume of 10 ml for every 6 million cells and incubated at room temperature for 10 min. Glycine was added to a final concentration of 125 mM to quench the formaldehyde, and then, the mixture incubated at room temperature for 5 min. The cells were pelleted at 2500 rpm for 5 min at 4 °C, and the supernatant was removed. Then, the pelleted cells were stored at − 80 °C or processed for use using the Hi-C protocol.

#### Chromatin digestion, dATP-tailing, and linker-mediated ligation

The cell pellet was resuspended in 550 μl of lysis buffer (10 mM Tris-HCl, pH 8.0; 10 mM NaCl; and 0.2% IGEPAL CA630 with proteinase inhibitor), incubated on ice for 20 min, and spun at 5000 rpm for 5 min at 4 °C. Then, the supernatant was discarded, and the nuclei were washed twice with 1× NEB CutSmart buffer. Then, the pellet was gently resuspended in 50 μl of 0.5% SDS and incubated for 10 min at 62 °C. After heating, 140 μl of H2O and 25 μl 10% Triton X-100 were added, mixed gently, and incubated for 15 min at 37 °C. Next, 25 μl of 10× NEB CutSmart buffer (NEB # B7204S) and 100 U of AluI (NEB Cat # R0137 L) were added to digest chromatin in less than 12 h at 37 °C and rotated at 700 rpm. AluI was inactivated by incubating the sample for 20 min at 62 °C. Nuclei were collected and washed twice with 1× NEB buffer 2. The nuclei were resuspended in 400 μl of Klenow (3′-5′ exo-) solution (40 μl f NEB buffer 2 (NEB # B7002S), 8 μl of 10 mM dATP (NEB # N0440S), 40 μl of 10% Triton X-100, 304 μl of H2O, and 8 μl of Klenow (3′-5′ exo-) (NEB Cat # M0212L)), and incubated at 37 °C for 1 h while rotating at 700 rpm. The nuclei were collected and washed twice with 1× T4 DNA ligase buffer and resuspended in 1200 μl of proximity ligation solution (120 μl of T4 DNA ligase buffer (NEB # B0202S), 120 μl of 10% Triton X-100, 940 μl of H_2_O, 6 μl of T4 DNA ligase (NEB # M0202), 12 μl of 10 mg/ml BSA, and 2 μl of bridge linker (200 ng/μl)), and rotated at 700 rpm at room temperature for 6 h. The nuclei were lysed gently with 500 μl of ice-cold NP-40 lysis buffer on ice for 5 min. The cell lysate was layered on top of 2.5 volumes of a sucrose cushion consisting of 24% (wt/vol) sucrose in NP-40 lysis buffer. This sample was centrifuged at 12,000 rpm for 10 min at 4 °C to isolate the nuclei pellet. The nuclear pellet was washed once with 1× PBS/1 mM EDTA. The nuclear pellet was resuspended gently with 0.5 ml of glycerol buffer followed by the addition of an equal volume of nuclei lysis buffer on ice for 2 min. The sample was centrifuged at 12,000 rpm for 2 min at 4 °C to isolate the chromatin pellet. The chromatin pellet was washed twice with PBS/1 mM EDTA.

#### Exonuclease digestion, DNA purification, and sonication

The supernatant was removed, and then, 70 μl of 10× lambda exonuclease buffer, 6 μl of lambda exonuclease (NEB # M0262S), 6 μl of exonuclease I (NEB # M0293S), and 618 μl of H2O were added. The mixture was rotated at 700 rpm at 37 °C for 1 h. The chromatin was pelleted at 12,000 rpm for 2 min at 4 °C. The chromatin pellet was washed twice with PBS/1 mM EDTA. To reverse the DNA cross-linking, the chromatin was resuspended in 1370 μl of digestion buffer (10 mM Tris-HCl, pH = 8; 25 mM EDTA; 1% SDS; and 1 mg/ml proteinase K (Life Technology # 25530-049)) and incubated for 30 min at 55 °C, and then, 130 μl of 5 M NaCl was added, and the mixture was incubated overnight at 65 °C. Genomic DNA was thoroughly extracted with an equal volume of phenol/chloroform/isoamyl alcohol and centrifuged at 12,000 rpm for 15 min at 4 °C. The aqueous top layer was poured into a new tube, and 1/10 vol of 3 M NaAc and 2 vol of 100% ethanol were added. The DNA was recovered by centrifugation at 12,000 rpm for 20 min at 4 °C. The pellet was rinsed with 75% ethanol, the ethanol was decanted, and the pellet was air-dried. The DNA pellet was dissolved in 200 μl of 10 mM Tris-HCl, pH 8, for 15 min at 37 °C. The DNA was sonicated by a Biorupter with the following settings: high energy, 30-s working time, 60-s intervals, and 2 cycles. The DNA on 2% agarose gel was measured and found to be of the expected length 0.2–2 kb.

#### Biotin pull-down assay

A total of 30 μl of 10 mg/ml Dynabeads M280 streptavidin (Thermo Fisher Scientific # 11205D) was added to a clean 1.5-ml microfuge tube and washed three times with 600 μl of 1× Tween wash buffer (5 mM Tris-HCl, pH 7.5; 0.5 mM EDTA; 1 M NaCl; and 0.05% Tween), one time with 2× BB (10 mM Tris-HCl, pH 7.5; 1 mM EDTA; and 2 M NaCl). The beads were separated using magnets, and the supernatant was discarded. The beads were resuspended in 200 μl of 2× BB, and then, the beads were transferred to a sample tube. The beads were incubated with a sheared DNA sample for 15 min at RT with rotation and separated with magnets, and then, the supernatant was discarded. The beads were washed five times with 2× SSC/0.5% SDS and three times with TE buffer, and then, they were transferred to a fresh tube after the last wash.

#### Tn5 tagmentation, PCR amplification, and high-throughput sequencing

The beads were washed in 100 μl of 1× TD buffer. The following master mix was prepared: 50 ng of proximity ligated DNA, 25 μl of tagmentation buffer, 5 μl of transposase enzyme (TDE1) (Illumina # FC-121-1030), and 20 μl of H_2_O adjusted to a total volume of 50 μl. The reaction was incubated at 55 °C for 10 min and then at 10 °C for 10 min in a PCR machine. The samples were placed on a magnet, and the supernatant was removed. The beads were washed twice with 2× SSC/0.5% SDS, twice with TE buffer, and transferred to a fresh tube after the last wash. The beads were resuspended with 30 μl of TB buffer. For PCR amplification with the following master mix: 10 μl of DNA library-coated beads, 10 μl of H_2_0, 15 μl of NPM mix, 5 μl of PPC PCR primer, 5 μl of Index Primer1 (i7), and 5 μl of Index Primer2 (i5) (Illumina # FC-121-1030), and the total volume was adjusted to 50 μl. The following PCR program was used: 72 °C for 3 min; 98 °C for 10 s, 63 °C for 30 s, 72 °C for 50 s for 10 cycles, and 72 °C for 5 min. The PCR product was run on a 2% agarose gel, and 300–500-bp DNA was purified with a Magen gel purification kit (Magen # D2111-03). The purified DNA was subjected to HiseqXten 150 × 150 pair-end sequencing.

### HiChIP

HiChIP was carried out based on a modified previously published protocol [51]. The cell harvest, lysis, endonuclease digestion, and bridge linker-mediated proximal ligation processes were the same as those described for the BAT Hi-C analysis. After lambda exonuclease and exonuclease I digestion for 1 h at 37 °C, the cells were properly sonicated in a Qsonica Q700 for the subsequent antibody enrichment as appropriate for ChIP-Seq. Enriched DNA was collected with a Magen purification kit (Cat # D2111-03), and qPCR was performed to evaluate the quality of the ChIP. Post-ChIP DNA was quantified to generate libraries with Qubit dsDNA HS Kit (Thermo Fisher # Q32851). Fifty nanograms of post-ChIP DNA was sufficient for library preparation (based on contact libraries generated for high quality, with chromatin that was not oversonicated and material that was robustly captured on streptavidin beads). After streptavidin enrichment, the library was prepared with a NEBNext Ultra II RNA Library Prep Kit for Illumina (NEB # E7770) following the recommended protocol. The PCR-amplified library product was run on a 2% agarose gel, and 300–500-bp DNA was purified with a Magen gel purification kit (Magen # D2111-03). The purified DNA was subjected to HiseqXten 150 × 150 pair-end sequencing.

### Open chromatin enrichment and network Hi-C (Ocean-C)

The Ocean-C method was modified based on a previously described protocol [53]. The cell harvest, lysis, endonuclease digestion, and bridge linker-mediated proximal ligation processes were the same as those described for the BAT Hi-C analysis. After lambda exonuclease and exonuclease I digestion for 1 h at 37 °C, the cells were sonicated by Biorupter to achieve an average DNA fragment size of 0.2–0.5 kb. The open chromatin purification and subsequent procedures were conducted as previously described [53]. After biotinylated DNA was pulled down with streptavidin beads, the library was completed and sequenced as described for the HiChIP library.

### ChIP-Seq

The ChIP procedure was modified based on a previously published protocol [80]. After cell fixation and chromatin fraction isolation, 40 U of micrococcal nuclease (MNase) was added to the chromatin fraction, incubated at 37 °C for 15 min while rotating at 700 rpm, and inactivated by adding 20 μl 0.5 M EDTA and 40 μl 0.5 M EGTA. The spun-down pellets were resuspended in 300 μl of sonication buffer. The solution was sonicated by a Biorupter with the following settings: high energy, 30-s working time, 60-s intervals, and 20 cycles. After centrifugation twice at 12,000 rpm for 10 min at 4 °C, the subsequent antibody enrichment and ChIP DNA collection procedures were conducted as previously described [80]. All the ChIP material or 20 ng of the input ChIP DNA was used to construct Illumina sequencing libraries using the published TELP method [81]. PCR-amplified libraries were gel extracted at 200–500 bp and eluted in 30 μl of water. The library quality and quantity were analyzed with Bioanalyzer and Qubit assays, and then, the library was sequenced using HiseqXten 150 × 150 pair-end sequencing.

### Hi-C, HiChIP, and Ocean-C data analyses

All Hi-C, HiChIP, and Ocean-C libraries were sequenced on an Illumina HiSeq 10X instrument (150-bp paired-end mode). First, the bridge linker sequences were trimmed using trimLinker tool in the ChIA-PET2 software [82], and paired-end reads containing at least one instance of the bridge linker in either end were adapted for further processing. All the resulting data were mapped and filtered using HiC-Pro (version 2.11) [83]. Briefly, the read pairs were independently aligned to the mm10 reference genome using the bowtie2 algorithm with the “-very-sensitive” option. To rescue the chimeric fragments spanning the ligation junction, the ligation site was detected, and the 5′ end fraction of the reads was aligned back to the reference genome. Unmapped, multiple mapped, and singleton reads were discarded. Uniquely aligned reads were then assigned to AluI restriction enzyme cut sites. In this step, pairs read from the dangling-end, self-circle ligation and PCR artifacts were filtered out, and only the valid read pairs involving two different restriction fragments were used to build the contact matrices. In this study, we called features (compartments and TADs) for the +/− auxin condition separately and used the union set of features in two conditions. We used “global” when referring to genome-wide; “large-scale” when referring to Compartments, TADs, or large loop domains (> 250 kb); and “small-scale” when referring to the gene-associated chromatin structures.

### Meta-feature analysis (TADs, loops, and Hi-C interactions among the RNAP clusters)

All the analyses described in this section were performed using the normalized O/E matrices generated using JUICER software. To study the distribution of Hi-C interactions around common structural features, namely, TAD domains and chromatin loops, we performed a two-dimensional (2D) meta-feature analysis by pilling up individual submatrices into an average matrix. In general, this method is similar to that of the meta-gene analysis that is commonly performed for acquiring ChIP-Seq data. For the meta-TAD analysis, we first created a union set of TAD coordinates identified in both the untreated and treated samples and then extracted 25-kb resolution Hi-C O/E maps for all the TADs and their neighboring regions, chosen to be of the same length as the TAD, after rescaling each TAD to a 90 × 90 submatrix. For display and visual consistency with the contact probability, we set the background levels of all interaction matrices to 0–1 and chose a proper color palette in ggplot2. Differences between the untreated and treated Hi-C data sets were computed by subtracting the normalized O/E signal. To understand whether high-density RNAP-binding hotspots reshaped the genome to establish a local, small-scale structural change, we performed paired-end spatial chromatin analysis (PE-SCAn), an algorithm combining ChIP-Seq data with Hi-C data [84]. At 25-kb resolution, all submatrices of the paired anchors of the RNAP clusters (strongest peak as determined by ROSE [59]) and 500 kb up- and downstream were extracted and averaged into a single meta-matrix. Briefly, one of the paired Hi-C reads was aligned to the RNAP peaks. Only reads that mapped within 50 kb up- or downstream of the RNAP peaks were selected for further analysis. This method reduced the set of the corresponding reads to those also aligned to RNAP hubs, resulting in a set of two distances (dx, dy) for all the Hi-C di-tags that were found within 500 kb of these regions for every intrachromosomal pair. From the distribution of dx and dy, a frequency matrix was calculated with a bin size of 25 kb, and the randomized data set was calculated by aligning the Hi-C data to haphazardly permuted peaks. The PE-SCAn analysis of mRNA and tRNA locus after the depletions of Pol II and Pol III is similar to that of RNAP-binding hotspots described above (related to Fig. 3a–c, g, h). To calculate the average enrichment of contacts around active promoters engage in long-range interactions, we first selected the promoters to overlap with loop anchors in RNA polymerases I/II/III Hi-C data sets and constructed intrachromosomal pairs of intervals between promoters and promoters. Then, we classified these pairs into two groups: intra-TAD (200 kb to 2 Mb) and long-range (2 to 10 Mb). To maximize resolution, we used the pooled H3K27ac HiChIP and Ocean-C data sets from two replicates to explore the interaction strengths at different pairs of promoters in this part. We then extracted the observed and the expected contacts in a 400 × 400 kb window and processed them as described above (related to Fig. 6a; Additional file 1: Fig. S6a). Aggregate H3K27ac HiChIP and Ocean-C contact maps around pairs of active gene promoters in the mESCs were separated into three equal slices based on the expression (each *N* = 833) (related to Fig. 6b; Additional file 1: Fig. S6b). Active gene promoters were also further subdivided into CTCF versus no CTCF based on the presence of a CTCF binding site within ± 5 kb of that promoter (related to Fig. 6c; Additional file 1: Fig. S6c). For the definition of active gene promoters and pairs of promoters bound/not bound by CTCF, see the average insulation analysis section in Additional file 3.

### ChIP-Seq data analyses

Pooled ChIP libraries were prepared and sequenced at a sequencing depth of ~ 90–100 million reads per sample. Raw fastq reads were trimmed by trim_galore and mapped to the mouse reference genome mm10 using bowtie2 (version 2.3.2) [85] with the default parameters “best –k 1 –m 1 and –l 18.” Unmapped reads, low-quality mapped reads, and PCR duplicates were discarded. Only uniquely mapped data were retained for the downstream analysis. In the next step, we carried out peak calling individually for each replicate against the input control using MACS2 (version 2.1.0) with the “-c” option and a *p* value threshold of 10^−5^ to ensure high confidence [86]. The peaks that overlapped a peak from the other replicate of the same RNAP sample by at least 1 bp were retained. In these cases, the new peak equaled the combined coordinates of all the overlapping peaks, considering both the number of biological replicates and the treatment. For H3K27ac broad peaks, we additionally used the “-broad” option. Finally, all peaks that matched the blacklist of artifactual regions in the mm10 database were filtered out. The ChIP reads at each genomic position were extended by 250 bp, normalized and converted to bigwig format using bamCoverage from the deepTools2 toolkit (version 3.1.3) [87]. For subsequent meta-gene analysis and browser visualization, replicates for each biological condition were merged into one bigwig track file. Coverage profiles surrounding the summit of the peaks (5 kb +/−) for use in the creation of heatmaps were extracted and calculated using computeMatrix. For comparison purposes, the larger library size is downsampled to match the smaller one. In the peak coverage analysis, to correct for differences in sequencing depth between the Pol and GFP-Pol ChIP-Seq, we downsampled reads from the Pol ChIP-Seq to obtain the equal number of reads between these two data sets. This implementation gives each Pol sample the same read depth and avoids normalization bias (related to Figs. 1d and 6c).

## Supplementary information

**Additional file 1: Fig. S1.** Characterization of the RNAP degron cell lines and uncropped images for western blot. **Fig. S2.** Quality controls for the Pol I, Pol II, and Pol III degradation Hi-C data sets. **Fig. S3.** Loop strength analyses of high-resolution chromatin interactions after Pol II depletion. **Fig. S4.** Quality controls for the Pol II degradation time course Hi-C data sets. **Fig. S5.** Examples of super-enhancer regions mildly enhancing chromatin interactions across loop domains after Pol II depletion. **Fig. S6.** Pol II depletion has relatively modest effect on promoter-associated chromatin interactions. **Fig. S7.** long-term depletion of Pol II leads to general disruption of chromatin accessibility.

**Additional file 2: Table S1.** RNA-Seq RPKM values for the Pol II untreated and degron cells. **Table S2.** Summary statistics of the Hi-C, HiChIP, and Ocean-C data. **Table S3.** Hi-C identified TADs (contact domains). **Table S4.** HiCCUPS identified loop domains. **Table S5.** Pol II PLAC-Seq high confidence interactions identified using the Origami pipeline. **Table S6.** RNAP ChIP-Seq peaks. **Table S7.** PCR primer sequences used in this study. **Table S8.** List of data sets used in this study.

**Additional file 3.** More method details.

**Additional file 4.** Review history.

## Data Availability

All next-generation sequencing data sets generated in this study have been deposited in NCBI Gene Expression Omnibus (GEO) database with accession GSE145874 [88]. All the other data generated in this study can be found in the manuscript and its supplementary files, including GSM747534, GSM747535 & GSM747536 [89], GSM1526287 [80], GSM766454 & GSM766455 [90], GSM3027975, GSM3027985, GSM3027986, GSM2587379 & GSM2587380 [22], GSM2295906 & GSM2295907 [56], GSM2644945, GSM2644946, GSM2644947 & GSM2644948 [24], GSM2203837, GSM2203838, GSM2434084 & GSE82185 [18], GSE98119 [22], GSM1625858 & GSM2156964 [91], GSM1665566 [92], and GSM2396701 & GSM2396700 [93] in GEO database. The cell lines have been authenticated and are available upon request.

## References

[1] Yu M, Ren B (2017). The three-dimensional organization of mammalian genomes. Annu Rev Cell Dev Biol.

[2] Dekker J, Mirny L (2016). The 3D genome as moderator of chromosomal communication. Cell.

[3] Rowley MJ, Corces VG (2018). Organizational principles of 3D genome architecture. Nat Rev Genet.

[4] Schoenfelder S, Fraser P (2019). Long-range enhancer-promoter contacts in gene expression control. Nat Rev Genet.

[5] Robson MI, Ringel AR, Mundlos S (2019). Regulatory landscaping: how enhancer-promoter communication is sculpted in 3D. Mol Cell.

[6] Zheng H, Xie W (2019). The role of 3D genome organization in development and cell differentiation. Nat Rev Mol Cell Biol.

[7] Szabo Q, Bantignies F, Cavalli G, Szabo Q, Bantignies F, Cavalli G (2019). Principles of genome folding into topologically associating domains. Sci Adv.

[8] Stadhouders R, Filion GJ, Graf T (2019). Transcription factors and 3D genome conformation in cell-fate decisions. Nature.

[9] van Steensel B, Furlong EEM (2019). The role of transcription in shaping the spatial organization of the genome. Nat Rev Mol Cell Biol.

[10] Li X, Fu XD (2019). Chromatin-associated RNAs as facilitators of functional genomic interactions. Nat Rev Genet.

[11] Hnisz D, Day DS, Young RA (2016). Insulated neighborhoods: structural and functional units of mammalian gene control. Cell.

[12] Hug CB, Grimaldi AG, Kruse K, Vaquerizas JM (2017). Chromatin architecture emerges during zygotic genome activation independent of transcription. Cell.

[13] Rowley MJ, Nichols MH, Lyu X, Ando-Kuri M, Rivera ISM, Hermetz K, Wang P, Ruan Y, Corces VG (2017). Evolutionarily conserved principles predict 3D chromatin organization. Mol Cell.

[14] Rowley MJ, Lyu X, Rana V, Ando-Kuri M, Karns R, Bosco G, Corces VG (2019). Condensin II counteracts Cohesin and RNA polymerase II in the establishment of 3D chromatin organization. Cell Rep.

[15] Busslinger GA, Stocsits RR, van der Lelij P, Axelsson E, Tedeschi A, Galjart N, Peters JM (2017). Cohesin is positioned in mammalian genomes by transcription, CTCF and Wapl. Nature.

[16] Heinz S, Texari L, Hayes MGB, Urbanowski M, Chang MW, Givarkes N, Rialdi A, White KM, Albrecht RA, Pache L (2018). Transcription elongation can affect genome 3D structure. Cell.

[17] Ke Y, Xu Y, Chen X, Feng S, Liu Z, Sun Y, Yao X, Li F, Zhu W, Gao L (2017). 3D chromatin structures of mature gametes and structural reprogramming during mammalian embryogenesis. Cell.

[18] Du Z, Zheng H, Huang B, Ma R, Wu J, Zhang X, He J, Xiang Y, Wang Q, Li Y (2017). Allelic reprogramming of 3D chromatin architecture during early mammalian development. Nature.

[19] Jung YH, Sauria MEG, Lyu X, Cheema MS, Ausio J, Taylor J, Corces VG (2017). Chromatin states in mouse sperm correlate with embryonic and adult regulatory landscapes. Cell Rep.

[20] Battulin N, Fishman VS, Mazur AM, Pomaznoy M, Khabarova AA, Afonnikov DA, Prokhortchouk EB, Serov OL (2015). Comparison of the three-dimensional organization of sperm and fibroblast genomes using the Hi-C approach. Genome Biol.

[21] Flyamer IM, Gassler J, Imakaev M, Brandao HB, Ulianov SV, Abdennur N, Razin SV, Mirny LA, Tachibana-Konwalski K (2017). Single-nucleus Hi-C reveals unique chromatin reorganization at oocyte-to-zygote transition. Nature.

[22] Vian L, Pekowska A, Rao SSP, Kieffer-Kwon KR, Jung S, Baranello L, Huang SC, El Khattabi L, Dose M, Pruett N (2018). The energetics and physiological impact of Cohesin extrusion. Cell.

[23] El Khattabi L, Zhao H, Kalchschmidt J, Young N, Jung S, Van Blerkom P, Kieffer-Kwon P, Kieffer-Kwon KR, Park S, Wang X (2019). A pliable mediator acts as a functional rather than an architectural bridge between promoters and enhancers. Cell.

[24] Nora EP, Goloborodko A, Valton AL, Gibcus JH, Uebersohn A, Abdennur N, Dekker J, Mirny LA, Bruneau BG (2017). Targeted degradation of CTCF decouples local insulation of chromosome domains from genomic compartmentalization. Cell.

[25] Zuin J, Dixon JR, van der Reijden MI, Ye Z, Kolovos P, Brouwer RW, van de Corput MP, van de Werken HJ, Knoch TA, van IWF, et al: Cohesin and CTCF differentially affect chromatin architecture and gene expression in human cells. Proc Natl Acad Sci U S A 2014, 111:996–1001. 10.1073/pnas.1317788111 PMC3903193 24335803

[26] Rao SSP, Huang SC, Glenn St Hilaire B, Engreitz JM, Perez EM, Kieffer-Kwon KR, Sanborn AL, Johnstone SE, Bascom GD, Bochkov ID (2017). Cohesin loss eliminates all loop domains. Cell.

[27] Adelman K, Lis JT (2012). Promoter-proximal pausing of RNA polymerase II: emerging roles in metazoans. Nat Rev Genet.

[28] Engel C, Neyer S, Cramer P (2018). Distinct mechanisms of transcription initiation by RNA polymerases I and II. Annu Rev Biophys.

[29] Hannan KM, Sanij E, Rothblum LI, Hannan RD, Pearson RB (1829). Dysregulation of RNA polymerase I transcription during disease. Biochim Biophys Acta.

[30] Harlen KM, Churchman LS (2017). The code and beyond: transcription regulation by the RNA polymerase II carboxy-terminal domain. Nat Rev Mol Cell Biol.

[31] Sharifi S, Bierhoff H (2018). Regulation of RNA polymerase I transcription in development, disease, and aging. Annu Rev Biochem.

[32] Turowski TW, Tollervey D (2016). Transcription by RNA polymerase III: insights into mechanism and regulation. Biochem Soc Trans.

[33] Zaborowska J, Egloff S, Murphy S (2016). The pol II CTD: new twists in the tail. Nat Struct Mol Biol.

[34] Roeder RG, Rutter WJ (1969). Multiple forms of DNA-dependent RNA polymerase in eukaryotic organisms. Nature.

[35] Nguyen, Giannoni F, Dubois M-F, Seo S-J, Vigneron M, Kédinger C, Bensaude O: <1996_NAR Pol2 inhibited by α amanitin.pdf>. 1996. 10.1093/nar/24.15.2924 PMC146057 8760875

[36] Bensaude O (2011). Inhibiting eukaryotic transcription: which compound to choose? How to evaluate its activity?. Transcription.

[37] Natsume T, Kiyomitsu T, Saga Y, Kanemaki MT (2016). Rapid protein depletion in human cells by auxin-inducible degron tagging with short homology donors. Cell Rep.

[38] Nishimura K, Fukagawa T, Takisawa H, Kakimoto T, Kanemaki M (2009). An auxin-based degron system for the rapid depletion of proteins in nonplant cells. Nat Methods.

[39] Ghavi-Helm Y, Klein FA, Pakozdi T, Ciglar L, Noordermeer D, Huber W, Furlong EE (2014). Enhancer loops appear stable during development and are associated with paused polymerase. Nature.

[40] Lee K, Chris CS, Hsiung HP, Raj A, Blobel GA: Dynamic enhancer–gene body contacts during transcription elongation. Genes & Development 2015, 29:1992–97. 10.1101/gad.255265.114 PMC4604340 26443845

[41] Phillips-Cremins JE, Sauria ME, Sanyal A, Gerasimova TI, Lajoie BR, Bell JS, Ong CT, Hookway TA, Guo C, Sun Y (2013). Architectural protein subclasses shape 3D organization of genomes during lineage commitment. Cell.

[42] Huang J, Jiang Y, Zheng H, Ji X (2020). BAT Hi-C maps global chromatin interactions in an efficient and economical way. Methods.

[43] Dixon JR, Selvaraj S, Yue F, Kim A, Li Y, Shen Y, Hu M, Liu JS, Ren B (2012). Topological domains in mammalian genomes identified by analysis of chromatin interactions. Nature.

[44] Bonev B, Mendelson Cohen N, Szabo Q, Fritsch L, Papadopoulos GL, Lubling Y, Xu X, Lv X, Hugnot JP, Tanay A, Cavalli G (2017). Multiscale 3D genome rewiring during mouse neural development. Cell.

[45] Naumova N, Imakaev M, Fudenberg G, Zhan Y, Lajoie BR, Mirny LA, Dekker J (2013). Organization of the mitotic chromosome. Science.

[46] Ballabeni A, Park IH, Zhao R, Wang W, Lerou PH, Daley GQ, Kirschner MW (2011). Cell cycle adaptations of embryonic stem cells. Proc Natl Acad Sci U S A.

[47] Whyte WA, Orlando DA, Hnisz D, Abdennur N, Lin CY, Kagey MH, Rahl PB, Lee TI, Young RA. Master transcription factors and mediator establish super-enhancers at key cell identity genes. Cell. 2013;153:307-19. 10.1016/j.cell.2013.03.035 PMC3653129 23582322

[48] Dhillon N, Raab J, Guzzo J, Szyjka SJ, Gangadharan S, Aparicio OM, Andrews B, Kamakaka RT (2009). DNA polymerase epsilon, acetylases and remodellers cooperate to form a specialized chromatin structure at a tRNA insulator. EMBO J.

[49] Moqtaderi Z, Wang J, Raha D, White RJ, Snyder M, Weng Z, Struhl K (2010). Genomic binding profiles of functionally distinct RNA polymerase III transcription complexes in human cells. Nat Struct Mol Biol.

[50] Raab JR, Chiu J, Zhu J, Katzman S, Kurukuti S, Wade PA, Haussler D, Kamakaka RT (2012). Human tRNA genes function as chromatin insulators. EMBO J.

[51] Mumbach MR, Rubin AJ, Flynn RA, Dai C, Khavari PA, Greenleaf WJ, Chang HY (2016). HiChIP: efficient and sensitive analysis of protein-directed genome architecture. Nat Methods.

[52] Fullwood MJ, Liu MH, Pan YF, Liu J, Xu H, Mohamed YB, Orlov YL, Velkov S, Ho A, Mei PH (2009). An oestrogen-receptor-alpha-bound human chromatin interactome. Nature.

[53] Li T, Jia L, Cao Y, Chen Q, Li C (2018). OCEAN-C: mapping hubs of open chromatin interactions across the genome reveals gene regulatory networks. Genome Biol.

[54] Rao SS, Huntley MH, Durand NC, Stamenova EK, Bochkov ID, Robinson JT, Sanborn AL, Machol I, Omer AD, Lander ES, Aiden EL (2014). A 3D map of the human genome at kilobase resolution reveals principles of chromatin looping. Cell.

[55] Lareau CA, Aryee MJ (2018). Hichipper: a preprocessing pipeline for calling DNA loops from HiChIP data. Nat Methods.

[56] Fang R, Yu M, Li G, Chee S, Liu T, Schmitt AD, Ren B (2016). Mapping of long-range chromatin interactions by proximity ligation-assisted ChIP-seq. Cell Res.

[57] Handoko L, Xu H, Li G, Ngan CY, Chew E, Schnapp M, Lee CW, Ye C, Ping JL, Mulawadi F (2011). CTCF-mediated functional chromatin interactome in pluripotent cells. Nat Genet.

[58] Tang Z, Luo OJ, Li X, Zheng M, Zhu JJ, Szalaj P, Trzaskoma P, Magalska A, Wlodarczyk J, Ruszczycki B (2015). CTCF-mediated human 3D genome architecture reveals chromatin topology for transcription. Cell.

[59] Whyte WA, Orlando DA, Hnisz D, Abraham BJ, Lin CY, Kagey MH, Rahl PB, Lee TI, Young RA (2013). Master transcription factors and mediator establish super-enhancers at key cell identity genes. Cell.

[60] Loven J, Hoke HA, Lin CY, Lau A, Orlando DA, Vakoc CR, Bradner JE, Lee TI, Young RA (2013). Selective inhibition of tumor oncogenes by disruption of super-enhancers. Cell.

[61] Nagashima R, Hibino K, Ashwin SS, Babokhov M, Fujishiro S, Imai R, Nozaki T, Tamura S, Tani T, Kimura H (2019). Single nucleosome imaging reveals loose genome chromatin networks via active RNA polymerase II. J Cell Biol.

[62] Li G, Ruan X, Auerbach RK, Sandhu KS, Zheng M, Wang P, Poh HM, Goh Y, Lim J, Zhang J (2012). Extensive promoter-centered chromatin interactions provide a topological basis for transcription regulation. Cell.

[63] Schoenfelder S, Furlan-Magaril M, Mifsud B, Tavares-Cadete F, Sugar R, Javierre BM, Nagano T, Katsman Y, Sakthidevi M, Wingett SW (2015). The pluripotent regulatory circuitry connecting promoters to their long-range interacting elements. Genome Res.

[64] Hsieh T-HS, Cattoglio C, Slobodyanyuk E, Hansen AS, Rando OJ, Tjian R, Darzacq X. Resolving the 3D landscape of transcription-linked mammalian chromatin folding. Mol Cell. 2020;78:539-553. 10.1016/j.molcel.2020.03.002 PMC7703524 32213323

[65] Boxer LD, Renthal W, Greben AW, Whitwam T, Silberfeld A, Stroud H, Li E, Yang MG, Kinde B, Griffith EC (2020). MeCP2 represses the rate of transcriptional initiation of highly methylated long genes. Mol Cell.

[66] Gerber A, Ito K, Chu CS, Roeder RG: Gene-specific control of tRNA expression by RNA polymerase II. Mol Cell 2020;78:1-14. 10.1016/j.molcel.2020.03.023 PMC7273519 32298650

[67] Justice M, Carico ZM, Stefan HC, Dowen JM (2020). A WIZ/Cohesin/CTCF complex anchors DNA loops to define gene expression and cell identity. Cell Rep.

[68] Weintraub AS, Li CH, Zamudio AV, Sigova AA, Hannett NM, Day DS, Abraham BJ, Cohen MA, Nabet B, Buckley DL (2018). YY1 is a structural regulator of enhancer-promoter loops. Cell.

[69] Schmitt AD, Hu M, Jung I, Xu Z, Qiu Y, Tan CL, Li Y, Lin S, Lin Y, Barr CL, Ren B (2016). A compendium of chromatin contact maps reveals spatially active regions in the human genome. Cell Rep.

[70] Ye BY, Shen WL, Wang D, Li P, Zhang Z, Shi ML, Zhang Y, Zhang FX, Zhao ZH (2016). ZNF143 is involved in CTCF-mediated chromatin interactions by cooperation with cohesin and other partners. Mol Biol.

[71] Kagey MH, Newman JJ, Bilodeau S, Zhan Y, Orlando DA, van Berkum NL, Ebmeier CC, Goossens J, Rahl PB, Levine SS (2010). Mediator and cohesin connect gene expression and chromatin architecture. Nature.

[72] Beagan JA, Duong MT, Titus KR, Zhou L, Cao Z, Ma J, Lachanski CV, Gillis DR, Phillips-Cremins JE (2017). YY1 and CTCF orchestrate a 3D chromatin looping switch during early neural lineage commitment. Genome Res.

[73] Guo Y, Xu Q, Canzio D, Shou J, Li J, Gorkin DU, Jung I, Wu H, Zhai Y, Tang Y (2015). CRISPR inversion of CTCF sites alters genome topology and enhancer/promoter function. Cell.

[74] Fan H, Lv P, Huo X, Wu J, Wang Q, Cheng L, Liu Y, Tang QQ, Zhang L, Zhang F (2018). The nuclear matrix protein HNRNPU maintains 3D genome architecture globally in mouse hepatocytes. Genome Res.

[75] Bonev B, Cavalli G (2016). Organization and function of the 3D genome. Nat Rev Genet.

[76] Gomez-Diaz E, Corces VG (2014). Architectural proteins: regulators of 3D genome organization in cell fate. Trends Cell Biol.

[77] Li L, Lyu X, Hou C, Takenaka N, Nguyen HQ, Ong CT, Cubenas-Potts C, Hu M, Lei EP, Bosco G (2015). Widespread rearrangement of 3D chromatin organization underlies polycomb-mediated stress-induced silencing. Mol Cell.

[78] Ray J, Munn PR, Vihervaara A, Lewis JJ, Ozer A, Danko CG, Lis JT (2019). Chromatin conformation remains stable upon extensive transcriptional changes driven by heat shock. Proc Natl Acad Sci U S A.

[79] Jin F, Li Y, Dixon JR, Selvaraj S, Ye Z, Lee AY, Yen CA, Schmitt AD, Espinoza CA, Ren B (2013). A high-resolution map of the three-dimensional chromatin interactome in human cells. Nature.

[80] Ji X, Dadon DB, Abraham BJ, Lee TI, Jaenisch R, Bradner JE, Young RA (2015). Chromatin proteomic profiling reveals novel proteins associated with histone-marked genomic regions. Proc Natl Acad Sci.

[81] Peng X, Wu J, Brunmeir R, Kim SY, Zhang Q, Ding C, Han W, Xie W, Xu F (2015). TELP, a sensitive and versatile library construction method for next-generation sequencing. Nucleic Acids Res.

[82] Li G, Chen Y, Snyder MP, Zhang MQ. ChIA-PET2: a versatile and flexible pipeline for ChIA-PET data analysis. Nucleic Acids Res. 2017;45:e4. 10.1093/nar/gkw809 PMC5224499 27625391

[83] Servant N, Varoquaux N, Lajoie BR, Viara E, Chen CJ, Vert JP, Heard E, Dekker J, Barillot E (2015). HiC-Pro: an optimized and flexible pipeline for Hi-C data processing. Genome Biol.

[84] de Wit E, Bouwman BA, Zhu Y, Klous P, Splinter E, Verstegen MJ, Krijger PH, Festuccia N, Nora EP, Welling M, et al. The pluripotent genome in three dimensions is shaped around pluripotency factors. Nature. 2013;501:227–31. 10.1038/nature12420 23883933

[85] Langmead B, Salzberg SL (2012). Fast gapped-read alignment with Bowtie 2. Nat Methods.

[86] Zhang Y, Liu T, Meyer CA, Eeckhoute J, Johnson DS, Bernstein BE, Nusbaum C, Myers RM, Brown M, Li W, Liu XS (2008). Model-based analysis of ChIP-Seq (MACS). Genome Biol.

[87] Ramirez F, Ryan DP, Gruning B, Bhardwaj V, Kilpert F, Richter AS, Heyne S, Dundar F, Manke T (2016). deepTools2: a next generation web server for deep-sequencing data analysis. Nucleic Acids Res.

[88] Yongpeng Jiang, Jie Huang, Kehuan Lun, Boyuan Li, Haonan Zheng, Yuanjun Li, Rong Zhou, Wenjia Duan, Chenlu Wang, Yuanqing Feng, Hong Yao, Cheng Li, Xiong Ji. Genome-wide analyses of chromatin interactions after the loss of Pol I, Pol II and Pol III. All the sequencing datasets including Hi-C, HiChIP, Ocean-C, RNA-seq, ChIP-seq, ATAC-seq, and 4C-Seq data, have been deposited in GEO. 2020. https://www.ncbi.nlm.nih.gov/geo/query/acc.cgi?acc=GSE145874. 10.1186/s13059-020-02067-3 PMC7331254 32616013

[89] Stadler MB, Murr R, Burger L, Ivanek R, Lienert F, Scholer A, van Nimwegen E, Wirbelauer C, Oakeley EJ, Gaidatzis D (2011). DNA-binding factors shape the mouse methylome at distal regulatory regions. Nature.

[90] Dowen JM, Bilodeau S, Orlando DA, Hubner MR, Abraham BJ, Spector DL, Young RA (2013). Multiple structural maintenance of chromosome complexes at transcriptional regulatory elements. Stem Cell Reports.

[91] Wu J, Huang B, Chen H, Yin Q, Liu Y, Xiang Y, Zhang B, Liu B, Wang Q, Xia W (2016). The landscape of accessible chromatin in mammalian preimplantation embryos. Nature.

[92] Sigova AA, Abraham BJ, Ji X, Molinie B, Hannett NM, Guo YE, Jangi M, Giallourakis CC, Sharp PA, Young YA. Transcription factor trapping by RNAin gene regulatory elements. Science. 2015;350:978-81. 10.1126/science.aad3346 PMC4720525 26516199

[93] Li X, Zhou B, Chen L, Guo LT, Li H, Fu XD: GRID-seq reveals the global rNA–chromatin interactome. Nat Biotechnol. 2017;35:940-50. 10.1038/nbt.3968 PMC5953555 28922346

