# Supplementary material for: Genome-wide analyses of chromatin interactions after the loss of Pol I, Pol II, and Pol III
Source: Genome Biol. 2020 Jul 2;21:158. doi: 10.1186/s13059-020-02067-3 (PMC7331254; doi:10.1186/s13059-020-02067-3)
Supplement: Supplementary file 1 — Additional file 1: Fig. S1. Characterization of the RNAP degron cell lines and uncropped images for western blot. Fig. S2. Quality controls for the Pol I, Pol II, and Pol III degradation Hi-C data sets. Fig. S3. Loop strength analyses of high-resolution chromatin interactions after Pol II depletion. Fig. S4. Quality controls for the Pol II degradation time course Hi-C data sets. Fig. S5. Examples of super-enhancer regions mildly enhancing chromatin interactions across loop domains after Pol II depletion. Fig. S6. Pol II depletion has relatively modest effect on promoter-associated chromatin interactions. Fig. S7. long-term depletion of Pol II leads to general disruption of chromatin accessibility. [file 13059_2020_2067_MOESM1_ESM.pdf]

Figure S1

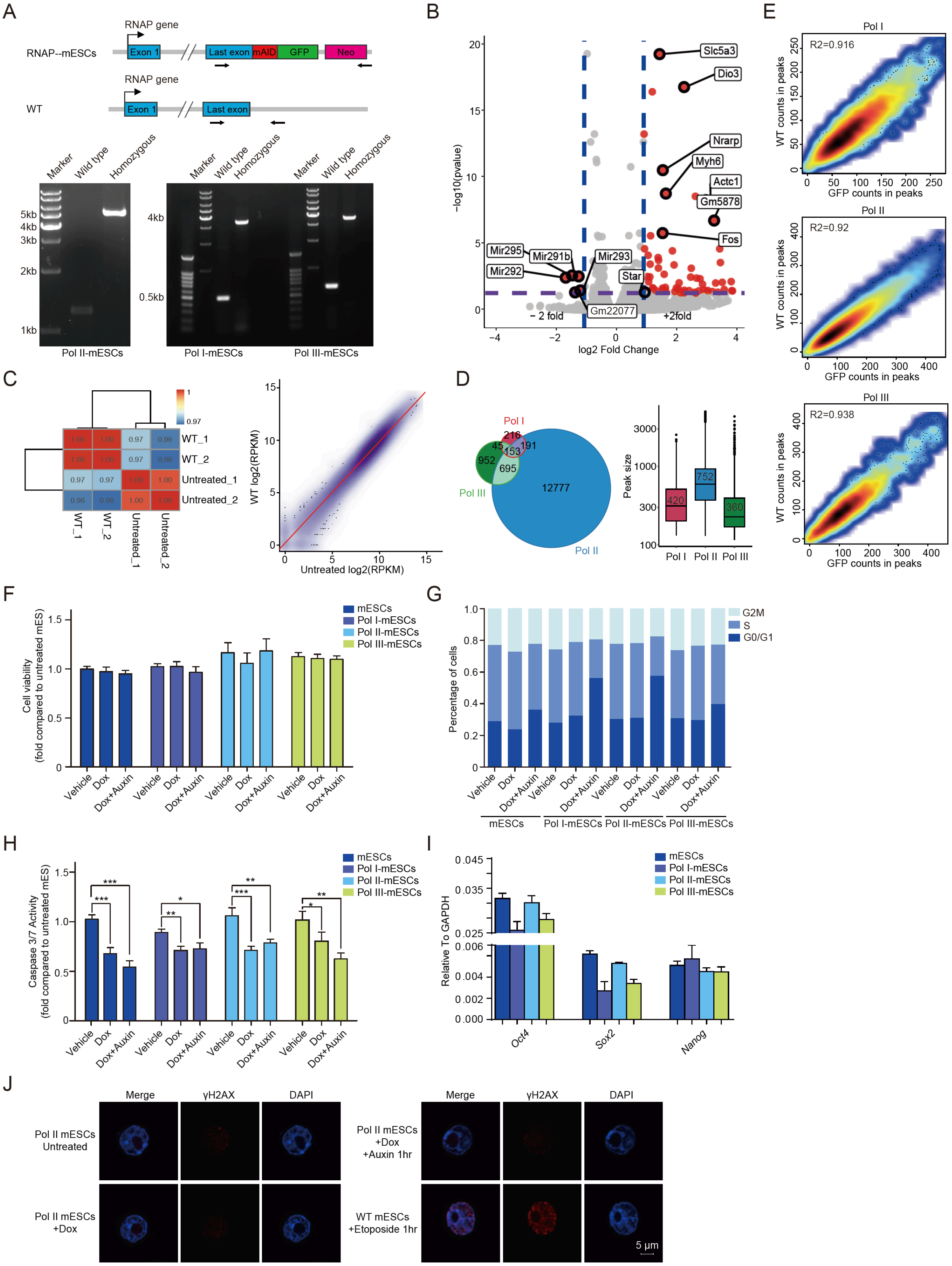

A
RNAP gene
RNAP--mESCs
Exon 1
Last exon
mAID
GFP
Neo
WT
Marker
Wild type
Homozygous
5kb
4kb
3kb
2kb
1kb
0.5kb
Pol II-mESCs
Pol I-mESCs
Pol III-mESCs
B
Slc5a3
Dio3
Nrarp
Myh6
Actc1
Gm5878
Fos
Mir295
Mir291b
Mir293
Mir292
Star
Gm22077
− 2 fold
+2fold
−log10(pvalue)
log2 Fold Change
C
WT_1
WT_2
Untreated_1
Untreated_2
WT log2(RPKM)
Untreated log2(RPKM)
D
Pol I
Pol II
Pol III
216
45
191
153
952
695
12777
Peak size
420
752
360
E
Pol I
R2=0.916
Pol II
R2=0.92
Pol III
R2=0.938
WT counts in peaks
GFP counts in peaks
F
mESCs
Pol I-mESCs
Pol II-mESCs
Pol III-mESCs
Cell viability
(fold compared to untreated mES)
Vehicle
Dox
Dox+Auxin
G
G2M
S
G0/G1
Percentage of cells
mESCs
Pol I-mESCs
Pol II-mESCs
Pol III-mESCs
H
Caspase 3/7 Activity
(fold compared to untreated mES)
I
Relative To GAPDH
Oct4
Sox2
Nanog
J
Merge
γH2AX
DAPI
Pol II mESCs
Untreated
Pol II mESCs
+Dox
Pol II mESCs
+Dox
+Auxin 1hr
WT mESCs
+Etoposide 1hr
5 μm

Fig. S1. Characterization of the RNAP degron cell lines.

a. Design of CRISPR knock-in for RNAP-mAID-GFP fusions in mESCs. Primer for RNAP-mAID-GFP genotyping indicated by arrowhead (top). Genotyping for Pol I-mAID-GFP, Pol II-mAID-GFP, and Pol III-mAID-GFP mESCs is shown in the bottom panel.

b. Volcano plot displaying poly-A RNA-Seq signals for untreated and Pol II depleted (auxin treated for 6 hr) mESCs.

c. Heatmap depicting the Pearson correlation coefficients of the RNA-seq samples, and scatter plots showing independent replicates of RNA-Seq read counts for 39,017 transcripts in wild-type and untreated Pol II mES cells.

d. Venn diagram showing the existence of common and specific peaks of Pol I, Pol II and Pol III, and a box plot showing the comparison of their peak size distributions.

e. Smooth scatter plot showing the ChIP-Seq signals of the wide-type and untreated cells that had a high correlation coefficient (>0.9). The Pearson correlation value for each pairwise comparison is shown in the top left corner.

f-h. Cell viability (**f**), cell cycle (**g**), and caspase 3/7 (**h**) analyses for wild-type, Pol I, Pol II, and Pol III mAID-GFP fusion mESCs after treatment with a vehicle, doxycycline, or doxycycline and auxin.

i. RT-qPCR analyses of *Oct4*, *Sox2*, and *Nanog* in wild-type, Pol I (RPA1), Pol II (RPB1), and Pol III (RPC1) mAID-GFP fusion mESCs.

j. Immunofluorescence staining for γH2AX (red) and with DAPI (blue) after 1 hr of individual drug treatment. Images were obtained using a 100 × oil objective.

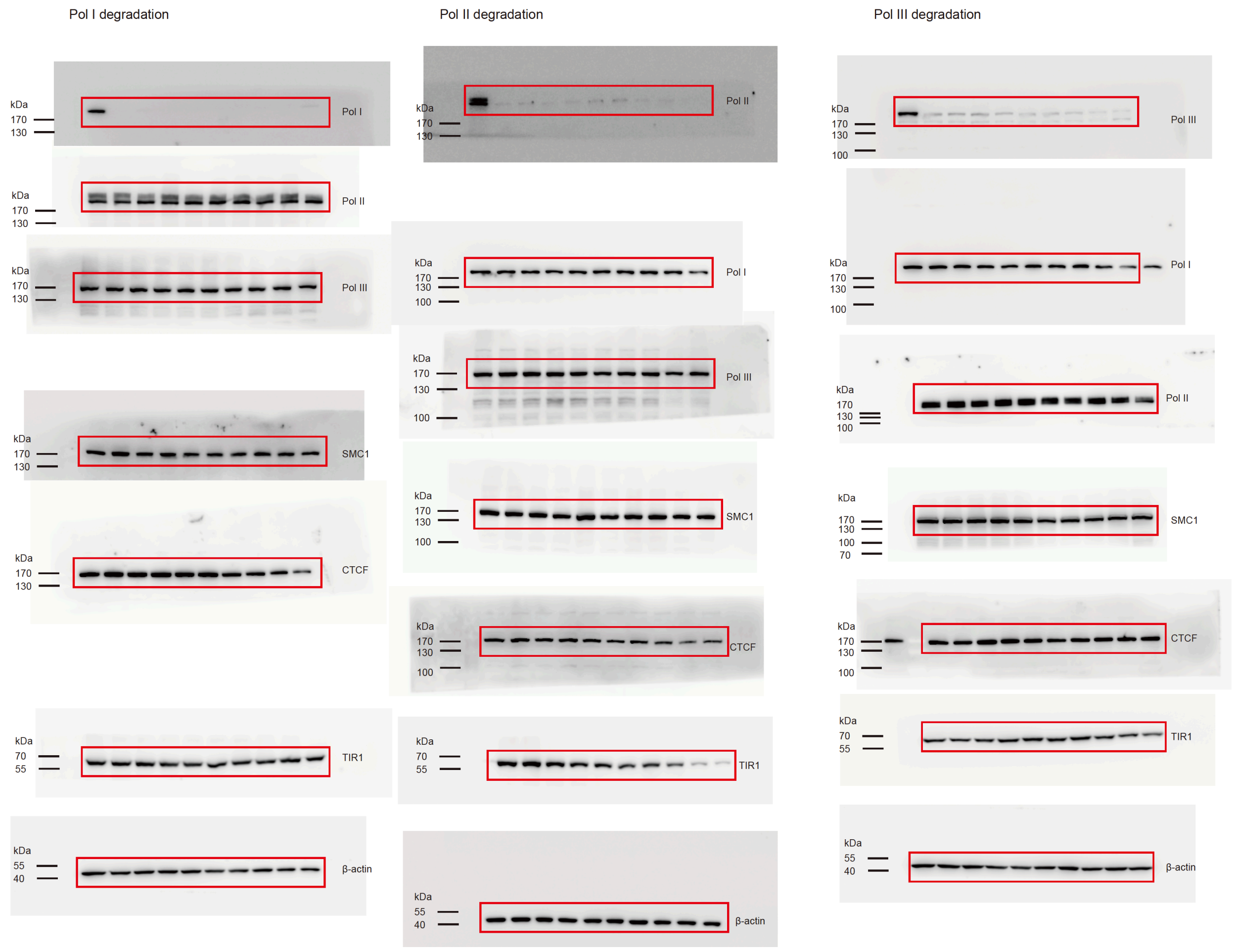

Fig. S1. Uncropped images for western blot data related to Figure 1b.

Fig S2

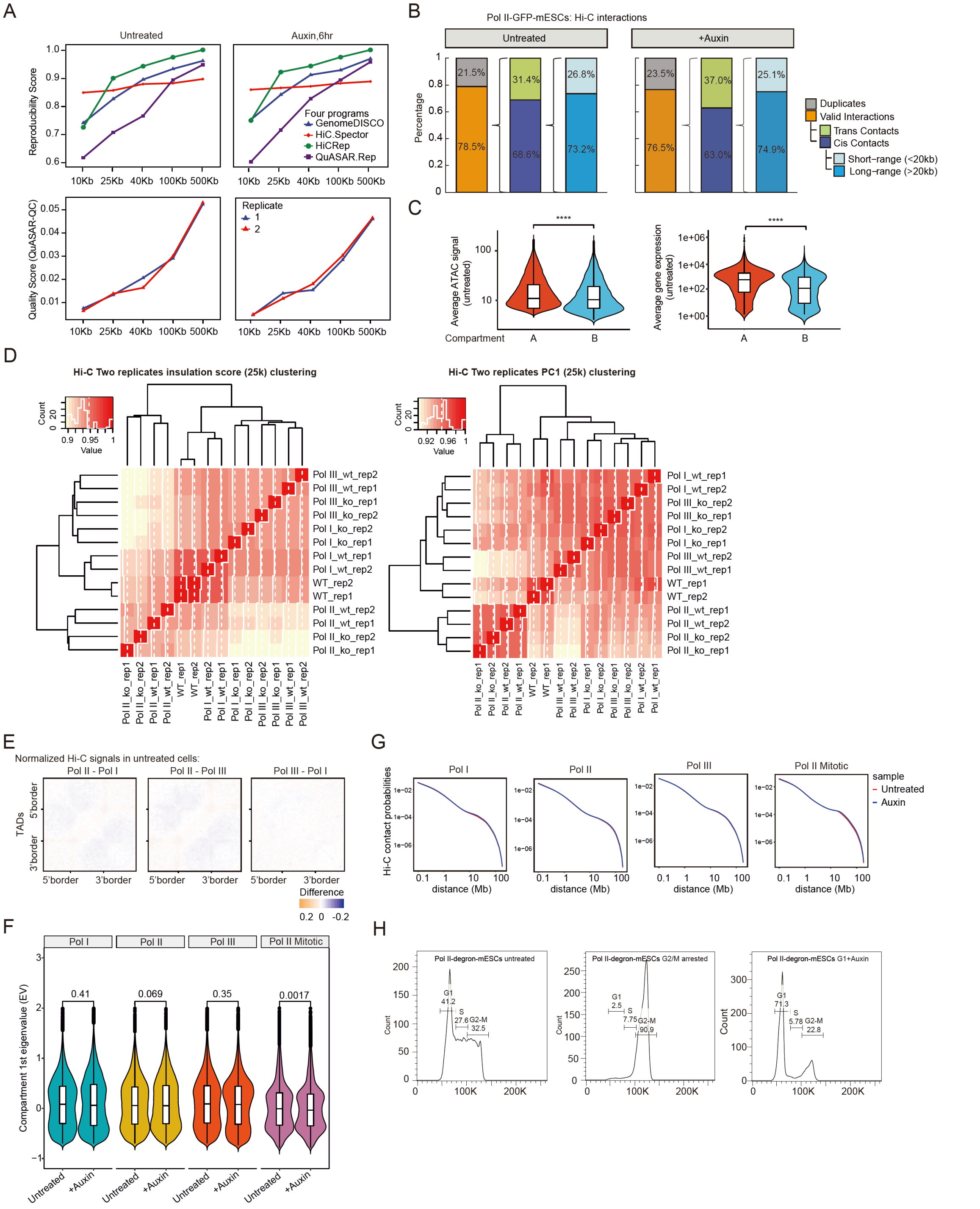

Fig. S2. Quality controls for the Pol I, Pol II, and Pol III degradation Hi-C data sets.
a. Reproducibility analysis of BAT Hi-C replicates. Reproducibility scores were calculated by four independent algorithms: including GenomeDISCO, QUASAR, and HiC.Spector

and HiC-Rep. Charts shows a plot of the reproducibility score (y-axis) at different resolutions (x-axis). All methods reported at least 60% of the reproducibility rate from a 10 kb to 500 kb resolutions. The quality score was measured by QUASAR.

b. The alignment statistics of the BAT Hi-C assay for the untreated (total reads=277 million) and Pol II-degraded (total reads=300 million) samples. Typically, the BAT Hi-C library contains over 75% of unique pairs with an optimal number of PCR cycles. Approximately 70 % of the valid pairs are cis contacts that consist of 26.8% short-range interactions (<20 kb) and 73.2% long-range interactions (>20 kb), while 30% of the pairs are consist of interchromosomal contacts.

c. Compartment A identified in this study had higher ATAC-Seq signals (left panel) and gene expression levels (right panel) compared with those of compartment B, indicating that our compartment identification was accurate. Significance was determined using a Wilcoxon test (****p < 0.0001).

d. Left: Hierarchical clustering of the compartment A/B scores (PC1 values) of the samples; Right: hierarchical clustering of the insulation scores of the samples. “wt” refers to the untreated condition, and “ko” refers to the auxin-treated condition.

e. Average observed/expected Hi-C interactions within TADs of Pol II-mAID mESCs untreated subtracted Pol I-mAID-mESCs (left) or Pol III-mAID-mESCs (middle) untreated ones. The average observed/expected Hi-C interactions within TADs of Pol III-mESCs untreated subtracted Pol I-mESCs untreated signals are shown in the right panel.

f. Violin plots showing the quantification of the A/B compartmental contact frequencies (presented as the 1st eigenvalues) under various RNAP degradation conditions in the BAT Hi-C data sets. Significance was calculated using a two-sided Wilcoxon test.

g. Overall scaling of the normalized Hi-C contact frequency as a function of genomic distance was not affected by RNAP degradation.

h. Cell cycle analyses for the mitotic exit experiment with Pol II degron cells.

Fig S3
A

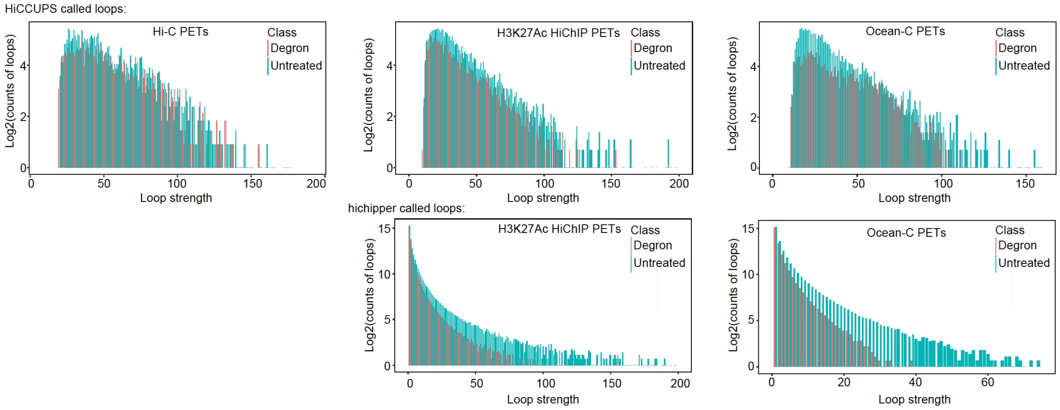

Fig. S3. Loop strength analyses of high-resolution chromatin interactions after Pol II depletion.

a. Loop strength distribution analyses of data from Hi-C (left), and H3K27ac HiChIP (middle) and Ocean-C (right) analyses. The x-axis denotes the loop strength, and the y-axis denotes the log counts of the loops with different strengths. Top: the loops were called by HiCCUPS; bottom: the loops were called by hichipper. Red bars indicate degron conditions, and blue bars indicate untreated conditions.

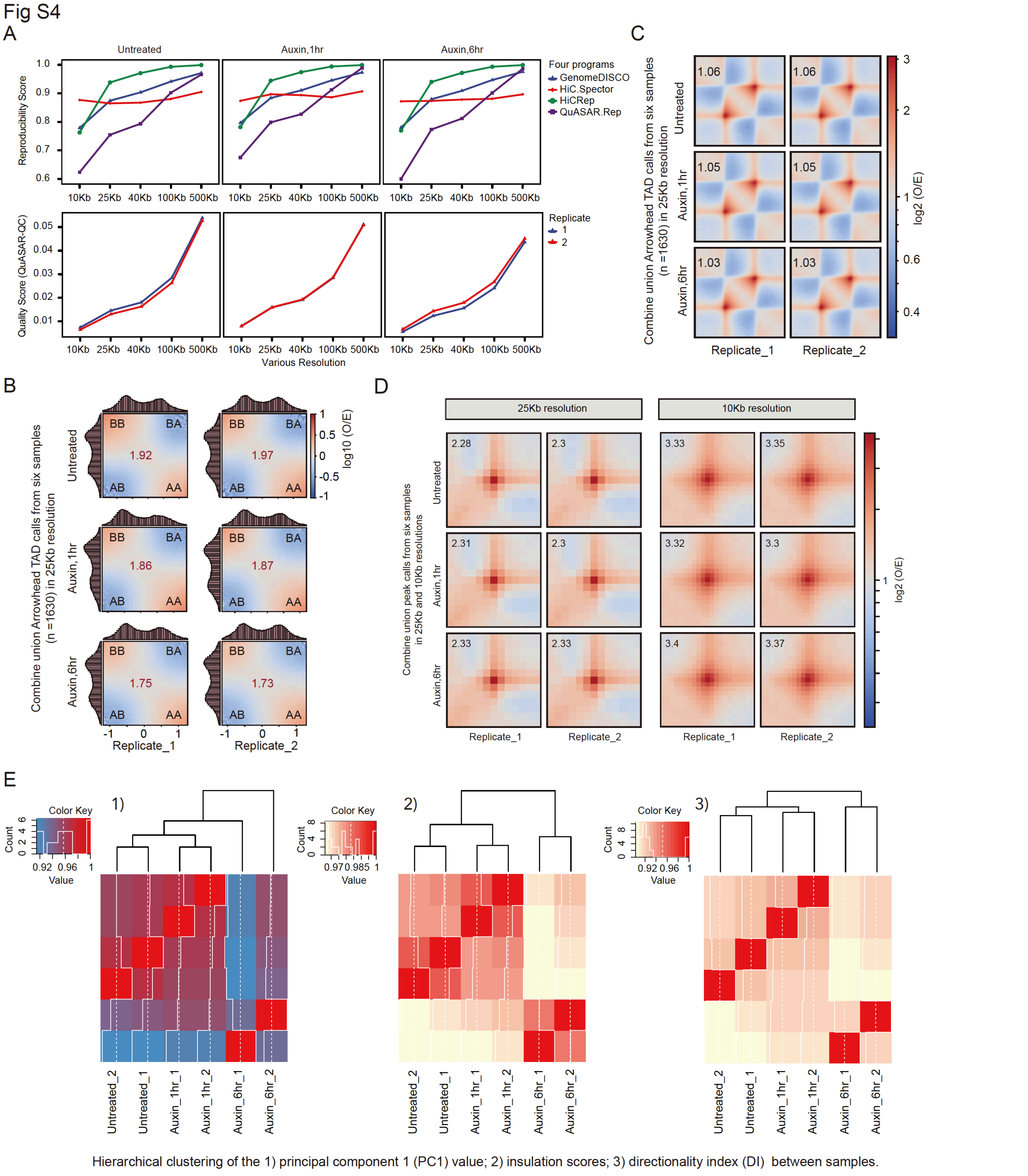

Fig. S4. Quality controls for the Pol II degradation time course Hi-C data sets.

a. Analysis is the same as that depicted in Fig. S2A, but with the time course from the Pol II degron Hi-C data sets.

b. Saddle plots representing compartmentalization strength according to the time course

from the Pol II degron Hi-C data sets.

c. Heatmap of the average observed/expected Hi-C interactions according to the time course from the Pol II degron Hi-C data sets.

d. Aggregated peak analysis (APA) of the union loop calls based on merged and two biological replicates from untreated and Pol II-degraded cells. Bin sizes were 10 kb and 25 kb. Numbers in the top left corner of each heatmap indicate average loop strength: log2(obs/exp). Color shows log-scale of the enrichment of interactions.

e. Hierarchical clustering of the 1) compartment A/B scores (PC1 values), 2) insulation scores, and 3) directionality index (DI) of the samples.

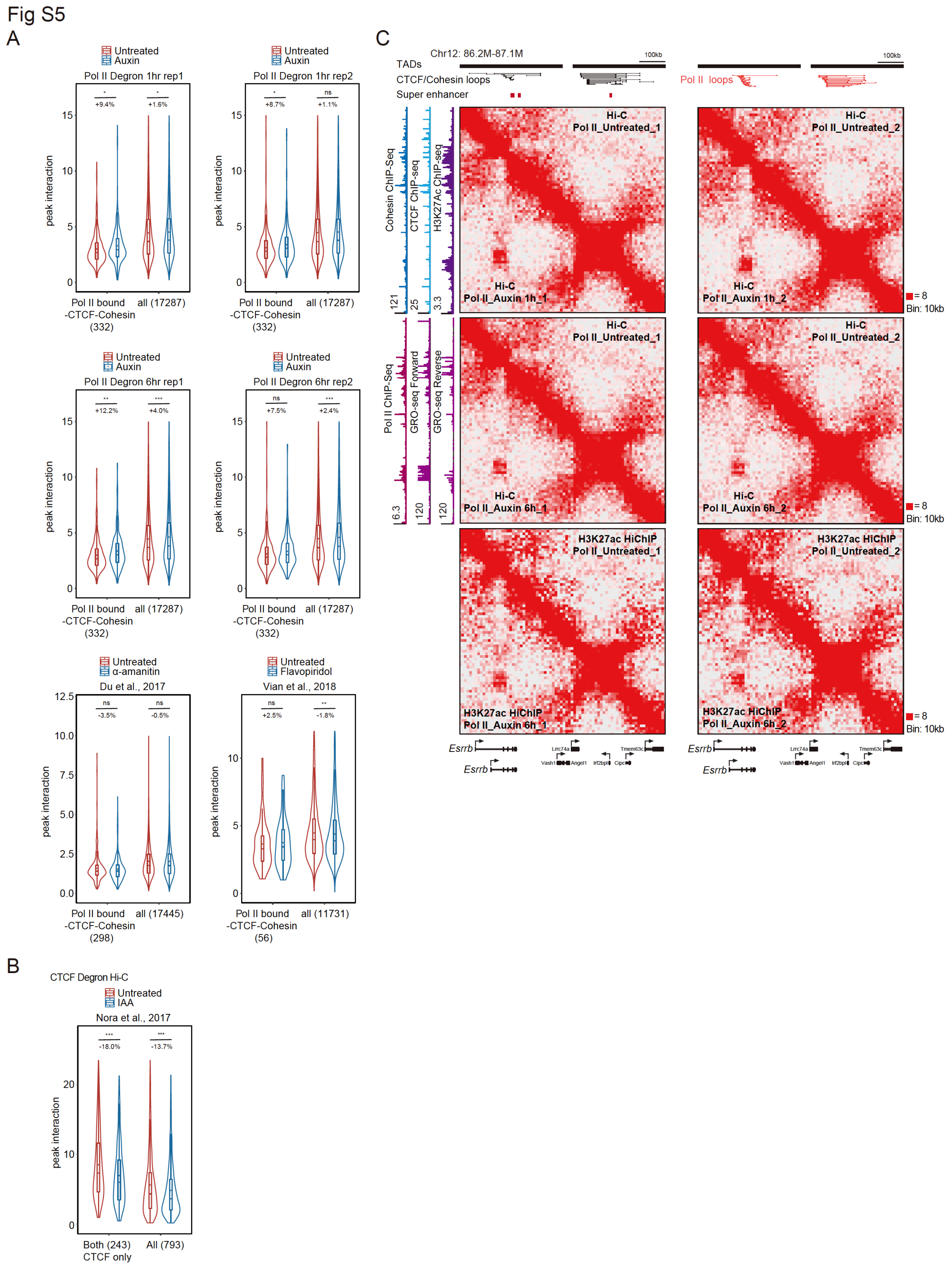

Fig. S5. Examples of super-enhancer regions mildly enhancing chromatin interactions across loop domains after Pol II depletion.

a. Violin plots of loop peak interactions from the Hi-C data under untreated and auxin (or

inhibitors) conditions. Loops of Pol II bound but without CTCF or Cohesin were selected and plotted with all loops included. The number of loops is indicated in parentheses. The dot inside the box plot denotes the mean interaction frequency. The percentage of change was calculated as the difference in the mean value divided by the untreated mean value. Significance was calculated by Student's t-test (***: <0.001, **: <0.01, *:0.05).

b. Violin plots of the loop peak interaction based on the CTCF Hi-C data of the untreated and degron conditions. Loops with CTCF bound at both anchors were selected out and plotted with all loops included. **b** is illustrated similar to **a**.

c. Hi-C (upper) and H3K27ac HiChIP (bottom) contact maps for the *Esrrb*: 86.2-87.1 Mb region of chromosome 2 at 10 kb resolution in the untreated and auxin-treated Pol II degron cell lines is shown. The Cohesin, CTCF, H3K27Ac ChIP-Seq signals, and GRO-Seq signals are displayed on the left. The black bars indicate as TAD structures. The black lines indicate CTCF/Cohesin loops, and the red lines indicate Pol II-associated loops.

Fig S6

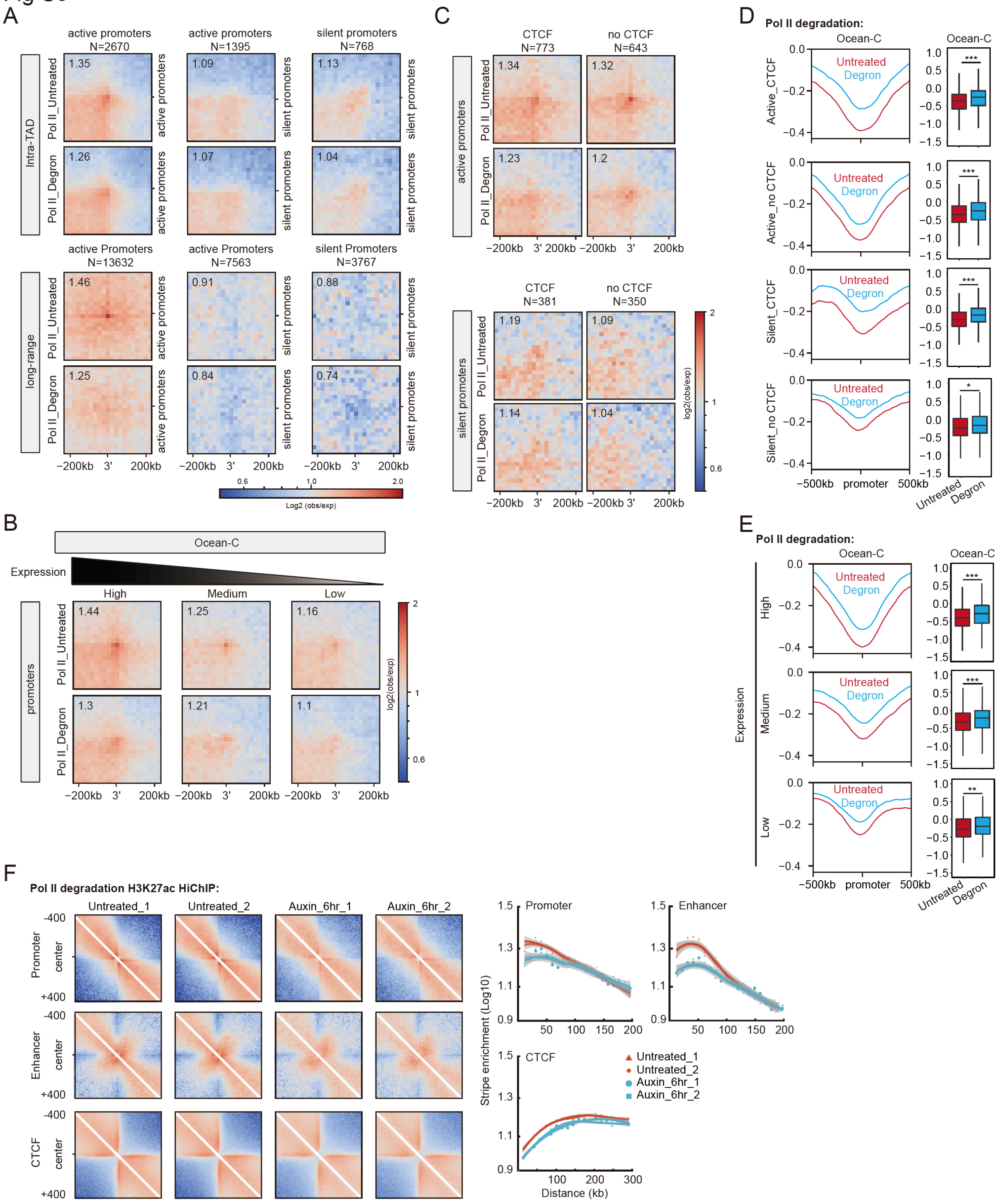

Fig. S6. Pol II depletion has relatively modest effect on promoter-associated chromatin interactions.

a. Aggregate Ocean-C contact maps around pairs of either active or silent gene promoters in the mESCs grouped by Intra-TAD pairs (between 200 Kb and 2 Mb) and cis long-range interactions (from 2 Kb to 10 Mb).

b. Aggregate Ocean-C contact maps around pairs of active gene promoters in the mESCs separated into three equal slices (each N=833) based on the gene expression level.

c. Aggregate Ocean-C contact maps around pairs of either active or silent gene promoters in the mESCs were classified based on the presence of CTCF-binding sites within ± 5 kb.

d. Average insulation score using the Ocean-C data set of different categories of promoters under untreated and Pol II-degraded conditions (6 hr) (left), and the insulation score at promoters was quantified as illustrated in the box plot shown in the right panel. Significance was determined using a Wilcoxon test (*$p < 0.05$, **$p < 0.01$, ***$p < 0.001$).

e. Average insulation score using the Ocean-C data set on promoters under untreated and Pol II-degraded conditions (6 hr) (left), and the insulation score for the promoters was quantified as illustrated in the box plot in the right panel. Significance was determined using a Wilcoxon test (*$p < 0.05$, **$p < 0.01$, ***$p < 0.001$).

f. Pile-up analysis of the target-centered chromatin structure. Maps are plotted at 10-kb resolution for long-range chromatin interactions (left panel). Signal decay curve of the gene stripe. The X-axis shows the normalized contact of the target stripe, and the y-axis shows the distance to the target center up to 200 kb (right panel).

Fig S7
A

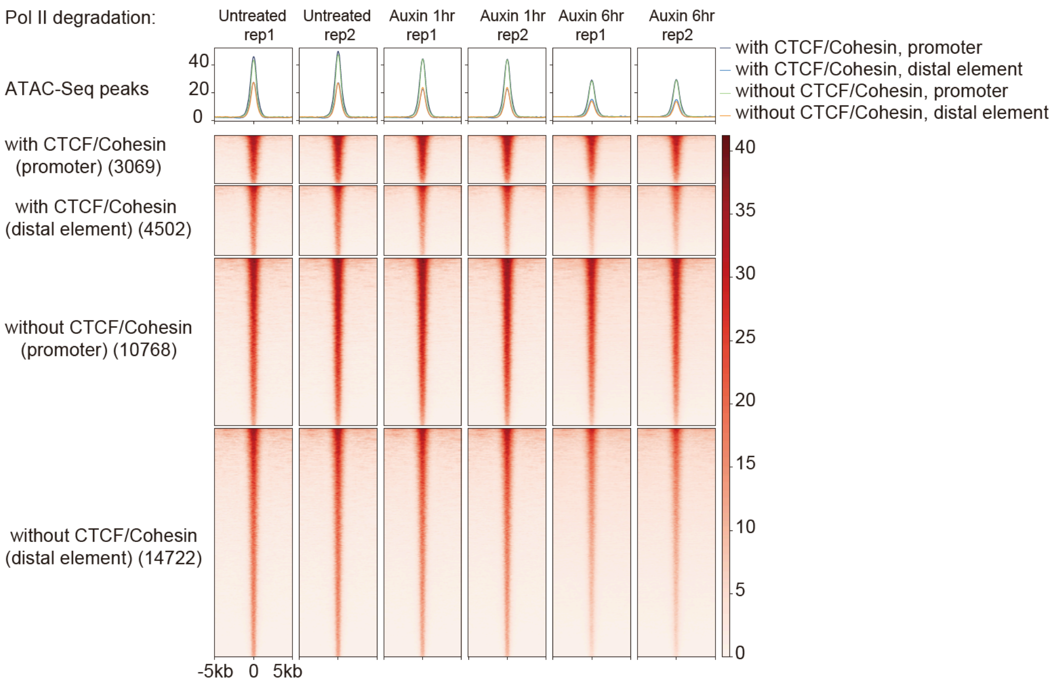

Fig. S7. long-term depletion of Pol II leads to general disruption of chromatin accessibility.

a. Signal profile and heatmap of ATAC-Seq peaks at the promoter or nonpromoter regions with and without CTCF/Cohesin binding. ATAC-Seq peaks were called separately under the three conditions and merged together.
