# Supplementary material for: Genome-wide analyses of chromatin interactions after the loss of Pol I, Pol II, and Pol III
Source: Genome Biol. 2020 Jul 2;21:158. doi: 10.1186/s13059-020-02067-3 (PMC7331254; doi:10.1186/s13059-020-02067-3)
Supplement: Supplementary file 3 — Additional file 3. More method details. [file 13059_2020_2067_MOESM3_ESM.pdf]

METHOD DETAILS

Key resources table

REAGENT or RESOURCE	SOURCE	IDENTIFIER
Antibodies		
Anti-POLR1A antibody	Santa Cruz	Cat # Sc-48385
Anti- Lamin b1 antibody	Santa Cruz	Cat # sc-374015
Anti- POLR2A antibody	Abcam	Cat # ab817
Anti-POLR3A antibody	Abcam	Cat # ab96328
Anti-H3K27ac antibody	Abcam	Cat # ab4729
Anti-GFP antibody	Abcam	Cat # ab290
Anti-CTCF antibody	Merck Millipore	Cat # 07-729
Anti-γH2AX antibody	Merck Millipore	Cat # 05-636
Anti-SMC1 antibody	Bethyl	Cat # A300-055A
Anti-HA tag antibody	Abcam	Cat # ab9110
Anti-β-Actin antibody	Sigma-Aldrich	Cat # A2228
Anti-Rabbit IgG Secondary Antibody	GE Healthcare	Cat # NA931V
Anti-Mouse IgG Secondary Antibody	GE Healthcare	Cat # NXA931V
Alexa Fluor 568 Donkey anti-Mouse IgG	Thermo Fisher	Cat # A10037
Alexa Fluor 594 Goat anti-Rabbit IgG	Thermo Fisher	Cat # A-11012
Chemicals		
Normal Mouse IgG	Merck Millipore	Cat # 12-370
Normal Rabbit IgG	Merck Millipore	Cat # 12-370
cOmplete Tablets EDTA-free, EASYpack	Roche	Cat # 04693132001
Propidium iodide	Sigma	Cat # P4170
DAPI (4',6-Diamidino-2-Phenylindole, Dilactate)	Thermo Fisher	Cat # D3571
Nocodazole	Sigma-Aldrich	Cat # M1404
Thymidine	Sigma-Aldrich	Cat # T1895
Actinomycin D	Sigma-Aldrich	Cat # 129935
5,6-Dichlorobenzimidazole 1-β-D-ribofuranoside (DRB)	Sigma-Aldrich	Cat # D1916
Flavopiridol	Selleck	S2679
Etoposide	Selleck	S1225
Doxycycline	Sigma-Aldrich	Cat # D9891
Indole-3-acetic acid sodium salt	Sigma-Aldrich	Cat # Cat # 1I5148

Puromycin dihydrochloride	Sigma-Aldrich	Cat # P8833
Hygromycin B	Thermo Fisher	Cat # 10687010
GENETICIN, G418	Thermo Fisher	Cat # 10131035
Critical Commercial Assays		
Dynabeads™ M-280 Streptavidin	Thermo Fisher	Cat # 11205D
Dynabeads™ Protein G	Thermo Fisher	Cat # 10004D
Pierce™ BCA protein assay kit	Thermo Fisher	Cat # 23227
Caspase-Glo® 3/7 Assay System	Promega	Cat # G8091
CellTiter-Glo® Luminescent Cell Viability Assay	Promega	Cat # G7570
FuGENE® HD Transfection Reagent	Promega	Cat # E2311
Illumina Nextera DNA Sample Preparation Kit	Illumina	Cat # FC-121-1030
NEBNext Ultra II DNA Library Prep Kit for Illumina	NEB	Cat # E7645S
KOD FX polymerase	TOYOBO	Cat # KFX-101
Fastpfu polymerase	TransGen Biotech	Cat # AP221-01
EasyTaq DNA Polymerase	TransGen Biotech	Cat # AP111-01
KAPA HIFI hotstart PCR Kit	Kapa Biosystems	Cat # KK2502
SuperScript™ III Reverse Transcriptase	Thermo Fisher	Cat # 18080085
2×RealStar Green Mixture	GenStar	Cat # A301-10
Megen Gel extraction kit	Megen	Cat # D2111-03
HiPure Plasmid EF Mini Kit	Magen	Cat # P1112-02
Qubit dsDNA HS kit	Thermo Fisher	Cat # Q32851

Plasmid Construction

The 66 amino-acids version of AID tag, mAID, developed in mammalian cells by Toyoaki Natsume was used here [1]. The Tir1 expression vector for degron parental cell line was constructed based on pGEM-T Easy vector (Promega A1360) by Gibson assembly with the following templates: Oryza Sativa Tir1 cDNA with TetO-3G promoter as well as the N-acteyl-transferase (PAC/PuroR) were PCR amplified from pMK243 (Natsume et al., 2016, Addgene # 72834), and 3x HA tag was added into C-terminal of Tir1 CDNA; homology arms to the Rosa26 locus were PCR amplified from mESCs V6.5 genomic DNA (400 bp each). The RNAPs-mAID-

EGFP targeting vector was constructed based on pGEM-T Easy vector (Promega A1360) by Gibson assembly with the following templates: the mAID-mClover (GFP) tag, as well as NeoR, were PCR amplified from pMK289 (Natsume et al., 2016, Addgene # 72827); homology arms to the last exon of Rpa1/Rpb1/Rpc1 were PCR amplified from mESCs V6.5 genomic DNA (400 bp each). We also constructed a version of the plasmid conferring resistance to Blasticidin (pMK291, Addgene # 72829). sgRNAs were constructed by annealing pairs of oligos in PX330 (Addgene # 42230), as described in [2]. The Rosa26-targeting sgRNA was cloned into pX330 by annealing caccGAAGATGGGCGGGA GTCTTC and aaacGAAGACTCCCGC CCATCTTC; Rpb1-targeting sgRNAs were cloned in PX330 by annealing oligos caccgATGAGGAGAACTGAGCGAAC and aaacGTTCGCTCAGTTCTCCTCATc for the first sgRNA and caccgTGAGGAGAA CTGAGCGAACA and aaacTGTTCGCTCAGTTCTCCTCAc for the second sgRNA. Similarly, Rpa1/Rpc1-targeting sgRNAs were created by these oligos: caccgAGAC AATGCTGCTATCTTAG and aaacCTAAGATAGCAGCATTGTCTc for the first sgRNA, caccgCAGACAATGCTGCTA TCTTA and aaacTAAGATAGCAGCATTGT CTGc for the second sgRNA for Rpa1 respectively; caccgCCCTCTTGTTACATAG TTTG and aaacCAAACTATGTAACAAGAGGGc for the first sgRNA, caccGTTAC ATAGTTTGTGGCAAG and aaacCTTGCCACAAACTATGTAAC for the second sgRNA for Rpc1 respectively.

Western blots

mESCs were dissociated, pelleted and resuspended in 2.5 mM $MgCl_2$, 0.25 M sucrose, 0.1% NP-40, 1 mM DTT, 25 mM HEPES pH 7.9, 700 mM NaCl, 1Xprotease inhibitor cocktail and swell for 10 minutes on ice, centrifuged at 14,000 rpm at 4 °C for 10 minutes. Protein concentration from supernatants was measured using the Pierce™ BCA protein assay kit (Thermo # 23227). Samples were mixed with loading buffer, run on 10% polyacrylamide SDS gel. Transfer onto PVDF membranes was performed with 300 mA 2 hours. Membranes were blocked with 5% skim milk in PBST for 1 hour at room temperature and then incubate with

primary antibody diluted in 5% Bovine serum albumin of PBST following manufacturer's recommendation overnight at 4 °C. The next day, the membrane was washed three times 5 minutes in PBS-0.1% Tween-20 at room temperature, incubated with secondary antibodies (1: 10,000) in 5% bovine serum albumin in PBS buffer supplementing with 0.1% Tween-20 1 hour at room temperature, washed 3 times and analyzed on G.E AI 600 RGB imaging system. Panels were mounted using imageJ preserving linearity.

Cell-Cycle analysis by propidium iodide staining

mESCs were trypsinized, resuspended in culture medium and pelleted in 2,500 g for 5 minutes, washed once with PBS buffer, and resuspended cell pellet in ice-cold PBS at 2 million cells/ml. Then 9× volume of 70% ethanol was added drop-wise while mixing and store cells overnight at -20 °C. Cells were collected at 500 g for 10 minutes at 4 °C, washed once with PBS, and the cell pellet was resuspended in 300 µl PBS supplementing with 0.1% Triton X-100, 20 µg/ml propidium iodide and 0.2 mg/ml RNase A. After incubation at 37 °C for 30 minutes, cells were identified directly with flow cytometry.

Mouse embryonic stem cell synchronization

Mouse embryonic stem cell synchronization was conducted as (Ballabeni et al., 2011) described. mES cells were treated with 1.25 mM Thymidine for 14 hours, followed by treatment with 50 ng/ml Nocodazole for 7 hours. Mitotic cells were collected at this time. G1 phase cells were collected 1 hour after release. Particularly, for Pol II degradation at the same time, Pol II degron cell line has already been treated with or without 500 µM indole-3-acetic acid (IAA) for 5 hours when synchronized into mitotic phase, and then release for 1 hour to get G1 phase synchronized mES cell with or without Pol II degradation respectively.

Caspase 3/7 activity and cell viability assays

Caspase 3/7 activity and cell viability assays for characterization of degron cell lines were carried out according to manufacturers' instruction, Promega G8091, and Promega G7570, respectively.

Immunofluorescence

mESCs were passaged on glass-coverslips, fixed with 4% paraformaldehyde in 1×PBS for 20 minutes at room temperature. 0.1% Triton-X100 was used for cell permeabilization following blocking with 5% BSA diluted in 1× PBS for 1 hour at 4 °C. Primary antibody (anti-Lamin b1, Santa cruz sc-374015, 1:100; anti-γH2A.X, Millipore 05-636, 1:500) incubation was conducted in 4% BSA overnight at 4 °C, followed by three times wash in 1× PBS for 5 minutes. Primary antibodies were recognized by secondary antibody (Alexa Fluor 568 Donkey anti-Mouse IgG Thermo Fisher A10037 1:500 dilution, Alexa Fluor 594 Goat anti-Rabbit IgG Thermo Fisher A11012 1:500 dilution) in the dark for 1 hour, three times wash in 1XPBS for 5 minutes, 1 μg/ml DAPI (Thermo Fisher # D3571) staining for 10 minutes at RT in the dark. Glass slides were mounted onto slides with VECTASHIELD® Antifade Mounting Medium (Vector Lab # H-1000). Coverslips were sealed with transparent nail polish and stored at 4 °C in the dark. Images were acquired at the Nikon A1RSi+ confocal microscope with 100× oil objective using NIS-Elements software and a Hammamatsu ORCA-ER CCD camera (Fenghuang Microscopy Facility, PKU), and then post-processed using Volocity, software available at https://www.winsite.com/authors/perkinelmer/1/ 3D-SIM images were acquired on an N-SIM imaging system (Nikon) equipped with a 100X/1.49 NA oil-immersion objective (Nikon) and four laser beams (405, 488,561 and 640). Images stacks with 0.12 μm intervals were acquired and computationally reconstructed to generate super-resolution optical serial sections. Images were post-processed using Imaris v9.3 Oxford Inc. (Fenghuang Microscopy Facility, PKU), software available at https://imaris.oxinst.com/.

4C-seq

We modified 4C-seq protocol based on 3C-HTGTS version [3]. We performed this assay with intact cell nuclei (in situ). Cells were cultured, harvested, and crosslinked as Hi-C described. Crosslinked mES cells lysis with 550 μl lysis buffer (10 mM Tris-HCl, pH 8.0, 10 mM NaCl, 0.2% IGEPAL CA630 with proteinase inhibitor) and incubate on ice for 20 minutes. Spin at 5000 rpm for 5 minutes at 4 °C to discard the supernatant. Washed Nuclei twice with 1× NEB Cutsmart Buffer. Then gently resuspend pellet with 50 μl 0.5% SDS and incubate for 10 minutes at 62 °C. After heating, add 140 μl H2O and 25 μl 10% Triton X-100, mix gently, incubate for 15 minutes at 37 °C. Add 25 μl 10× NEB Cutsmart Buffer (NEB # B7204S) and 100 U NlaIII (NEB Cat # R0125S) and digest chromatin less than 12 hours at 37 °C at 700 rpm. Inactivate NlaIII by incubating the sample for 20 minutes at 62 °C. Nuclei were collected, washed nuclei twice with 1× T4 DNA ligase buffer. Resuspended in 1200 μl of Proximity ligation solution (120 μl T4 DNA ligase buffer (NEB # B0202S), 120 μl 10% TritonX-100, 942 μl H2O, 6 μl T4 DNA ligase (NEB # M0202), 12 μl 10 mg/ml BSA), and Rotate at 700 rpm at room temperature for 6 hours. Then reverse crosslink and DNA purification were performed as Hi-C described. DNA was dissolved with 200 μl 10 mM Tris-HCl, pH 8.0 and sonicated by Biorupter with the following program: High energy, 30 seconds working time, 60 seconds interval, 2 cycles. Check DNA on 2% agarose gel. It should be 0.2-2 kb. The library construction starts with 40 μg sonicated DNA in each sample. Primer extension was performed firstly as the following program:

	1× (μl)	8× (μl)
5x Fast pfu buffer	10	80
dNTPs (2.5mM each)	1	8
Bio-primer (1μM)	1	8
Fast pfu polymerase	0.5	4
sonicated DNA	25	200
H2O	12.5	100

Set up the PCR program: 95 °C for 2 minutes; [95 °C for 30 seconds, 58 °C for 30 seconds, 72 °C for 1.5 minutes] (80 cycles); 72 °C for 2 minutes; 10 °C forever;

Pool the eluted DNA into one 1.5 ml tube, add 400 μl 5 M NaCl (1 M final) and 8 μl 0.5 M EDTA (5 mM final), mix with 20 μl washed C1 beads on the rotary mixer at

RT for at least 2 hours; Capture the beads on the magnet and wash with 300 μl 1xB&W buffer three times; Wash with 300 μl Tris-HCl (10 mM, pH 7.4) once and resuspend the beads in 30 μl Tris-HCl (10 mM, pH 7.4). And then perform bridge linker on beads ligation with the following system: 5 μl 10× ligation buffer, 2 μl 50 μM Adapter, 2.5 μl T4 DNA ligase, 15 μl 50% PEG8000, set up the ligation on the PCR machine, 25 °C for 1 hour, 22 °C for 2 hours, and then 16 °C overnight. Add 50 μl 2x B&W buffer and 100 μl 1× B&W buffer to collect the ligation products, then capture the beads on the magnet and wash with 200 μl 1× B&W buffer twice and wash with 200 μl Tris-HCl (10 mM, pH 7.4) once and resuspend the beads in 50 μl Tris-HCl (10 mM, pH 7.4). Then Nested PCR was performed as the following program:

	1× (μl)	2× (μl)
5x Fast pfu buffer	10	20
dNTPs (2.5mM each)	2	4
I7-Blue (10μM)	2	4
I5-Red (10μM)	2	4
Easytaq polymerase	0.5	1
sonicated DNA	25	50
H2O	8.5	17

Set up the PCR program: 95 °C for 2 minutes; [95 °C for 30 seconds, 58 °C for 30 seconds, 72 °C for 1 minutes] (15 cycles); 72 °C for 2 minutes; 10 °C forever; PCR product was purified with Megen gel purification kit (Megen # D2111-03) and eluted with 30 μl Tris-HCl (10 mM, pH 7.4). At last, the Tagged PCR was performed to add sequencing adaptor as the following program:

	1× (μl)	2× (μl)
5x Fast pfu buffer	10	20
dNTPs (2.5 mM each)	2	4
P7-I7 (10 μM)	2	4
P5-I5 (10 μM)	2	4
Fast pfu polymerase	0.5	1
sonicated DNA	17.5	35
H2O	16	32

Set up the PCR program: 95 °C for 2 minutes; [95 °C for 30 seconds, 58 °C for 30 seconds, 72 °C for 1 minute] (15 cycles); 72 °C for 2 minutes; 10 °C forever; PCR product was run on 2% agarose gel and recovery 500-700 bp product, eluted with

30 µl Tris-HCl (10 mM, pH 7.4) twice.

Library quality and quantity were analyzed with Bioanalyzer and Qubit assays and then sequenced on HiseqXten 150×150 pair-end sequencing (Related primers see additional file 2: Table S7).

-/+ Auxin GFP ChIP-Seq

The barcoded untreated/+auxin nuclei were mixed for multiplexed Pol II-GFP ChIP-seq with GFP antibody to avoid the variations for the library preparation and sequencing. The protocol was modified based on RELACS (restriction enzyme-based labeling of chromatin in situ) method published previously [4]. Briefly, nuclei were extracted using sonication (Biorupter) under these sonicator parameters: high power, 4 cycles/burst, for 30 seconds of treatment and 30 seconds interval. Nuclei were pelleted and resuspended in 50 µl of 0.5% SDS and incubated at room temperature for 10 min. SDS was quenched by adding 25 µl of 10% Triton X-100 and 145 µl of water. 25 µl of restriction enzyme buffer (CutSmart, NEB B7204S) and 2.5 µl of 100× protease inhibitor cocktail were added prior to enzyme incubation. 100 U AluI (NEB # R0137L) was added and digested for 12 hr at 37 °C. After dATP-tailing as Hi-C described, parallel adaptor ligation was performed with annealing adaptor (TRUSEQ-UNI-ADAPTER: "AATGATACGGCGACCACCGAGATCTACA CTCTTTCCCTACACGACGCTCTTCCGATCT"; Parallel reverse adaptor: "CAAG CAGAAGACGGCATACGAGATNNNNNNGTGACTGGAGTTCAGACGTGTGCTC TTCCGATC") at 37 °C for 6 hr. The barcoded untreated/+auxin intact nuclei were mixed for subsequent multiplexed Pol II-GFP ChIP-seq with GFP antibody. After de-crosslinking, libraries were amplified with parallel primer (forward: AATGATACGGCGACCACCGAGATCTACACTCTTTCCCTACACGACGCTCTTC CGATCT; reverse: GTGACTGGAGTTCAGACGTGTGCTCTTCCGATC) and run on 2% agarose gel and recovery 300-500 bp product, eluted with 30 µl Tris-HCl (10 mM, pH 7.4) twice. Library quality and quantity were analyzed with Bioanalyzer and Qubit assays and then sequenced on HiseqXten 150×150 pair-end

sequencing.

RNA-seq and RT-qPCR

Total RNA was isolated using TRIzol following the manufacturer's recommendations. Sequencing libraries were generated by Novogene corporation. The libraries were sequenced on an Illumina HiseqXten platform, and 150 bp paired-end reads were generated. For RT-qPCR assays, reverse transcription was performed using SuperScript™ III Reverse Transcriptase (Thermo # 18080085) with oligo-(dT)-18 primers, according to manufacturers' instructions. Quantitative real-time PCR was performed using 2× RealStar Green Mixture (GeneStar # A301-10) and Bio-Rad CFX Connect™ Real-Time PCR Detection System. For RT-qPCR primer sequences, please see additional file 2: Table S7.

ATAC-Seq

ATAC-seq was performed according to the protocol from [5]. 50,000 viable cells were used for library preparation using Nextera™ DNA Sample Prep Kit. PCR amplified libraries were extracted with Megen gel purification kit (Megen # D2111-03) without size selection. Library quality and quantity were analyzed with Bioanalyzer and Qubit assays and then sequenced on Illumina HiSeqXten using 150 bp paired-end mode.

Hi-C, HiChIP, and Ocean-C Library Preparation and Data Processing

Hi-C Data Normalization

Because different Hi-C experiments had various sequencing depths, we randomly sampled equal numbers of long-range (>20 kb) intra-chromosomal read pairs (n=75 million) from each sample for most downstream analyses involving comparison. To examine global interaction patterns, we divided the reference genome into contiguous, and equally sized bins range from 5 kb to 100 kb, then assigned the resampled read pairs to these bins and generated contact maps. At

last, the raw Hi-C contact matrices were normalized using the iterative correction method with *iced* script to correct for systematic biases such as GC content of trimmed ligation junction and the distance between restriction fragments. The procedure is based on the assumption that all loci should have equal visibility since we are detecting the entire genome in an unbiased manner. Having evaluated previously established matrices for Hi-C map resolution and the independent biological replicates for reproducibility [6], we speculated that the resolution of our Hi-C contact maps is between 10 kb and 25 kb, therefore, contact domain interactions and chromatin loops were called at the following resolutions: 10 kb and 25 kb. The ratio of cis- and trans-unique pair-end reads, which is a critical feature for the signal-to-noise ratio, was 3.58±1.39 for BAT-Hi-C (average ratio for 22 BAT Hi-C libraries generated in this study).

HiChIP and Ocean-C Data Normalization

For comparison of HiChIP/Ocean-C data under untreated and auxin(/inhibitors) conditions, we randomly subsampled equal numbers of long-range (>20 kb) intra-chromosomal read pairs (HiChIP: 49,907,498; Ocean-C: 52,837,002), and the sampled data were used to all the downstream analysis. Furthermore, the sampled allValidPairs file was converted to .hic file using hicpro2juicebox.sh (supplied by HiC-Pro) for calling loops with HiCCUPS.

Observed/Expected (O/E) matrix generation

For each chromosome, we calculated the average contact probability for all loci at a certain distance as expected interaction value. Then we transform the ice normalized Hi-C matrix into an observed/expected matrix by dividing each normalized observed by its corresponding expected value at that distance.

A/B compartment identification

The most common method for A/B compartment identification is the principal

component (PC) approach previously described [6, 7]. For each chromosome, the Hi-C O/E matrix at given resolution was first converted into a correlation matrix, where each entry (i, j) represents the Pearson correlation between row i and j of the original matrix. Then the signs of the dominant eigenvector were used to call compartments. In most cases, the first component of a PCA (PC1 eigenvector) on each of these matrices was used as a quantitative measure of compartmentalization and published mESC Hi-C compartment pattern was used as a reference to assign negative and positive PC1 to the correct categories. If necessary, the sign of the PC1 (which was randomly assigned) was inverted so that positive PC1 values corresponded to A (active)-compartment regions, and negative values fell into B. To quantify the strength of compartmentalization, we rearranged the rows and the columns of Hi-C O/E matrix for each chromosome in the order of increasing eigenvector value, and computed a strength ratio using the equation (AA + BB)/(2*AB), where AA refers to the average enrichment of loci whose two bins both lie in the top 20% of eigenvector scores, BB refers to bottom 20%, and AB to that of loci whose two bins have strong A and B compartment signal [8]. Finally, we aggregated the rows and the columns of the resulting matrix into 50 equally sized bins, thus obtaining a compartmentalization saddle plot. Furthermore, to ensure the robustness of this identification, compartment scores were calculated with eigenvector decomposition method (Related to Figures 2a-c,2f; Additional file 1: Fig. S4b).

Insulation score and TAD boundary calling

Normalized Hi-C contact matrices at 25 kb resolution were used to define TADs; the insulation score was calculated by averaging contacts in a quadratic sliding window along the diagonal of the matrix, then divided by the chromosomal average and log2-transformed as described before[6]. Directionality index-based domain boundaries were called from the vector of insulation scores following a hidden Markov model. Subsequently, we transformed valid pairs from HiC-Pro to juicer-

ready .hic files followed by annotating contact domains in our untreated and treated maps using arrowhead with default parameters [9]. Domains were called for Pol I, Pol II and Pol III, then merged from both untreated and treated samples, respectively (see additional file 2: Table S3). Aggregate meta-TAD plots in Figure 2 and additional file 1: Fig. S4, and contact probability differences were made based on individual RNAP Hi-C interaction files and merged domain boundary coordinates. (Related to Figures 2a-c, 2f; additional file 1: Fig. S4c).

Reproducibility of Hi-C data

Generally, Hi-C reproducibility between biological replicates was determined by flattening the Hi-C matrices to vectors and calculating the Pearson correlation coefficient between the vectors. However, it may produce misleading results due to distance dependence. To determine the degree of similarity between Hi-C replicates, we computed the insulation scores and compartment PC1 eigenvector values in 25 kb bins (see above), then calculated their Pearson correlation between each replicate of a different sample, which showed relatively high levels of reproducibility. Based on the Manhattan distance matrix, hierarchical clustering by complete linkage was conducted using an R heatmap2 function (Related to additional file 1: Fig. S2d, S4e).

Local stripe analysis

In a typical Hi-C assay, stripes are visible at the level of hundreds of kb, and their anchors usually are 10-30 kb wide. To make it comparable with H3K27ac HiChIP, we took normalized interaction matrices at the resolution of 10 kb to identify local stripes. As previously described, stripes correspond to lines extending from the diagonal in contact maps are thought to exhibit clear patterns whereby consecutive pixels displayed signal higher relative to the surrounding area. We first extracted the chromatin interaction maps of each “horizontal stripe” and “vertical stripe” that colocalize with the target sites of interest (e.g., Promoter, Enhancer, CTCF

Insulator or Super-enhancers) at 10 kb width to obtain 400 kb snippets from the Hi-C and H3K27ac HiChIP data, then calculated the interaction decaying behaviors of these stripes with distance. For the definition of target sites, see Definition of regulatory regions section. Stripe calls were performed with the balanced contact matrices that generated from .cool files using the COOLER package (https://github.com/mirnylab/cooler). All corresponding local stripes were piled-up together and plotted in python with custom functions. (Related to Figure 5c; additional file 1: Fig. S6f).

Representative regions quantification and comparison

Representative example graphs were selected to show the changed distribution of Hi-C interactions in the given regions. sub-TAD boundaries were called using the insulation score, the blue line defined the qualitative sub-TAD region, while yellow lines labeled boundaries called by the algorithm. Moreover, the domain boundary that we specified was demarcated with a blue arrow. The quantification of Hi-C contacts was measured by KR-normalized interaction matrix of untreated and degron cells, red-dot represented the mean contact frequency of the qualitative region in two groups. P values were determined using Wilcoxon test. The sources of the datasets used in those figures are listed in additional file 2: Table S8 (Related to Figures 3a-c, 3f).

Chromatin loops identification

We called chromatin loops in both untreated and treated samples by HICCUPS (Juicer 1.9.9) with default parameters. Briefly, for each entry in the Hi-C matrix, HICCUPS calculates several enrichment values over different local neighborhoods. Each enrichment value is associated with an FDR value for assessing statistical significance. We obtained 2483 loops in our untreated maps and 1941 loops in treated ones, which are comparable to the number of loops identified in other studies. To create a union loop set, we floored the coordinates of anchors to 10 kb

resolution and merged loops called from both untreated and treated samples together, generating 3768 loops in total (loops used in Figure 4). In another Hi-C experiment, we obtained a sum of 18801 for two replicates of untreated, 1 hour, and 6 hours of auxin treated Hi-C data together and floored the anchor coordinates to 10kb resolution, and finally generated 17287 loops. In the Hi-C dataset from Du et al., 2017, we extracted the interaction frequency of loop coordinates from our mES Hi-C data and plotted the interaction changes. The total 18801 loops were converted to mm9 and then floored to 10kb resolution and resulted in 17445 loops in total. In the datasets from Vian et al., 2018 and Nora et al., 2017, loops are called with HiCCUPS by their own Hi-C data matrix (Related to Figure 5b; additional file 1: Fig. S5a) [8, 10]. Then these loops were further identified by overlapping with ChIP-Seq peaks at loop anchors. The “Both-Pol II only” loops were defined as Pol II bound both anchors, but Cohesin unbound. The “Pol II bound-CTCF-Cohesin” loops were defined as Pol II bound at either anchor, but CTCF and Cohesin unbound at either anchor (Related to Figure 5b; additional file 1: Fig. S5a).

We applied the same methods described above to HiChIP and Ocean-C datasets. For HiChIP, we obtained 9600 in total that is merged from 6749 in untreated and 4349 in treated; for Ocean-C, we obtained 8077 loops in total, which are merged from 6216 loops in untreated and 2922 loops in treated (loops used in Figure 4).

To improve consistency and accuracy in our study, and to make the conclusion more reliable, results of HiChIP and Ocean-C from HICCUPS were compared to those identified by a second loop-calling pipeline – hichipper [11]. These two strategies incorporate different assumptions in identifying significant interactions and depend on the selection of thresholds. For HiChIP, 5998967 and 5895954 loops were called in untreated and treated maps by hichipper, respectively. For Ocean-C, 5062167 and 4474635 loops were called in untreated and treated maps by hichipper, respectively (Related to additional file 1: Fig. S3).

Differential Loop Analyses

The VCsqrt normalized observed over expected (O/E) interactions were extracted by Juicer dump from .hic files and used for analyses in this section. For each loop, the interaction of two anchors, also known as peak, are plotted for comparison in two conditions, as shown in scatter plots and box plots in Figure 4a. Normalized contact frequency represents log (Untreated interaction*Degron interaction*100), in which 100 is only multiplied by for a reasonable scale of displaying. Change in interaction frequency denotes the log2 transformation of (Degron interaction counts) / (Untreated interaction counts).

We next investigated the changes of the overall structure by calculating the mean interaction inside the whole loop domain, i.e., the mean value of every pixel p(x,y) with left anchor ≤ x ≤ right anchor and left anchor ≤ y ≤ right anchor in a contact heatmap. Mean interactions of two conditions are ordered according to their anchor distance and fitted with loess regression, as in Figure 4b.

To account for the difference of mean interactions of especially loops with length under 250 kb, we classified loops with high and low transcription signals into Top and Bottom group, respectively. For the definition of these two groups, see GRO-seq Data Analyses section. Distributions of lengths and mean interaction differences in two conditions of each loop are compared between top and bottom groups (Related to Figure 4c). All analyses in this section are done by custom R scripts.

RNA-seq Data Analysis

All RNA-seq samples with two replicates were sequenced on Illumina 10X using paired-ended 150 bp mode. The resulting FASTQ reads were mapped to the mm10 reference genome using hisat2 (version 2.1.0) [12]. First, we created hisat2 splice sites file from the GENCODE known transcripts, then used salmon to generate the counts and htseq-count to quantify reads at gene level defined by GENCODE. Differential expression testing was performed using DESeq2

bioconductor package [13]. A threshold of Benjamini-Hochberg corrected p-value< 0.05, and log2(FC) > 1 was chosen to determine significantly changed gene expression after treatment. Given the high reproducibility, transcript-based read counts of two replicates were pooled, RPKM normalized, and log2 transformed as described above for visualization. Very low expressed genes (~10%) were filtered out to reduce noises in fold change calculation for comparison between untreated and treated cells (Related to additional file 1: Fig. S1b).

ATAC-seq Data Analyses

ATAC-seq reads were aligned using Bowtie2 to mm10 and filtered using the same criteria as for ChIP-seq. In addition, reads mapping to the mitochondrial genome (ChrM) was removed. To restrict the analysis to regions spanning only one nucleosome, we required an insert size no larger than 140 bp, as we observed that this improved sensitivity to call peaks and reduced noise. De novo motif search was conducted using findMotifsGenome.pl from in the HOMER package with the parameters "-size 200 -mask -p 10" [14]. Coverage tracks were computed with reads per kilobase of transcript per million mapped reads (RPKM) using bamCoverage. Average ATAC-Seq signals within +/- 100 kb of loops (same regions used when defining transcription groups) with high and low transcription are calculated by multiBigwigSummary with the argument --outRawCounts (related to Figure 6C). ATAC-Seq peaks are called separately in each condition, and each replicate, then the peaks of two replicates in one condition are filtered with IDR (Irreproducible Discovery Rate) threshold of 0.05. The peaks of untreated, auxin 1h and 6h are merged together to reduce bias in any condition. Overlap with promoters or CTCF/Cohesin co-bound sites are defined as peaks that have at least 1 bp overlap of ±2.5 kb of TSS or CTCF/Cohesin co-bound sites, the rest were defined as distal elements or without CTCF/Cohesin binding.

Chromatin accessibility and Gene expression in A/B compartments

As known in previous studies, the A compartment is gene-rich, and its chromatin is more open, while the B regions are gene-poor and, its chromatin is more condensed. Thus, we examined for each chromosome separately whether A/B compartmentalization has such a relationship with chromatin accessibility and gene expression in our data. ATAC-seq, and RNA-seq signal was calculated as RPKM for the entire genome. For each compartment 25 kb bin, the average ATAC-seq and RNA-seq signals were computed and assigned to A or B group as is shown in violin plots. Both median value and reads coverage of chromatin accessibility and gene expression in our untreated sample were significantly higher in A compartment compared to B (p-value < 2.2e-16, Wilcoxon rank-sum test) (Related to additional file 1: Fig. S2c).

Hi-C and other multi-omics data correlation analysis

We next reasoned that RNAP related interaction changes -- as part of the RNA transcriptional machinery should also be enriched in regions of active transcription or open chromatin. We, therefore, aggregated ChIP-seq, ATAC-seq, and GRO-seq signals (bigwig-formatted) in RNA polymerase binding clusters. For consistency with the other data sets, mESC GRID-seq dataset, which downloaded from (http://fugenome.ucsd.edu/gridseq/), was converted to mm10 genome assembly and then re-binned into 1 bp windows. Pearson Correlation of signal-per-bin across the genome between Hi-C interaction changes and other multi-omics data signals were performed and plotted with heatmap.2 function in R using the ward.D2 method and Euclidian distance. Data parsing was carried out using the R packages *data.table* and *reshape2* (Related to Figure 3e).

Average insulation analysis

For insulation score analyses (related to Figure 2a-c, 2e), we downloaded the TAD file form Bonev., et al., 2017 [15], and classified these TADs boundaries into CTCF bound and CTCF-unbound TAD boundaries based on whether there is a CTCF binding site within in 12.5 kb. To analyses the promoters that have a more reliable

insulation score, we selected the promoters to overlap with loop anchors in RNA polymerases I/II/III Hi-C datasets and used the HiChIP and Ocean-C dataset to explore the insulation changes in different kinds of promoters. We used the active and silent genes defined by Whyte et al., 2012 [16] and further classified loop anchors into active and silent based on which kinds of promoters that they overlap, and only loops which only overlap with silent promoters were defined as silent loops. If multi promoters in one loop anchor, we only selected the highest expression gene for active promoter analyses and the lowest expression gene for silent promoter analyses and further classified them into CTCF bound and CTCF unbound based whether there is a CTCF binding site within 5 kb. We also selected the promoters overlapping with loop anchors and keep the highest expression genes if multiple promoters in one loop anchor. Then we select the promoters without CTCF binding sites within 5kb and classify them into three equal parts for the insulation score plot (related to Figure 6d-e; additional file 1: Fig. S6d-e). For visualization, we draw the average insulation score profile with deeptools2 toolkit (version 3.1.3) [17]. And the insulation score in promoters was quantified with multiBigwigSummary form deeptools2 toolkit (version 3.3.0) [17] and illustrated with a box plot.

Data visualization

Hi-C and HiChIP heatmaps were Knight-Ruiz (KR) normalized and visualized by Juicebox [18], in which the x- and y-axes represent loci in genomic order, and each pixel is the number of observed interactions between them. Hi-C meta-plots were created using custom in-house R scripts. Scores were set from zero to one equaling the lowest and highest values across plots in a panel set. Beside 2D chromatin interaction data, density tracks of 1D genomic data such as RNA-Seq for specific region were generated using custom software based on BEDTools bamCoverage program to count the number of reads in 1 bp windows normalized to library size to obtain densities in units of RPKM. For ATAC-seq or ChIP-seq

tracks, density tracks were generated differently by using a normalizing scale factor to calculate RPGC (Reads per genome coverage) across the genome, and lastly, snapshots were made with the UCSC genome browser. TAD files were download from Dixon et al., 2012, CTCF/Cohesion loop file were downloaded from Dowen et al., 2014, Pol II loop file was downloaded from Fang et al., 2016 (Related to Figures 4e, 5d; additional file 1: Fig. S5c).

4C-seq Data Analysis

The paired-end fastq files were demultiplexing with fastq-multx (version 1.3.1), the enzyme site was trimmed with trimlinker from ChIA-PET2 software [19], and the paired-end reads with at least one enzyme site and bait sequences were selected. The selected reads were mapped to the mm10 with Bowtie2 using the parameter "--very-sensitive-local -L 30 --score-min G,20,8". The mapping reads were filtered the relegation reads, self-ligation reads, dumped reads, and duplicated reads. Final bam files were converted into wig files with pyicos (version 2.0.7)[20], and then the wig files were performed quantile normalization with DANPOS2 (version 2.2.2) [21]. For visualization more conveniently, we converted the wig files into bigwig files with wigToBigWig (Related to Figure 5e).

GRO-seq Data Analyses

GRO-seq data analyses were performed, as previously reported. GRO-seq data were downloaded from the GEO datasets, and the single-end fastq file was cut adaptors and filter low quality reads with cutadapt (version 1.15). Filtered reads were mapped to mm10 with Bowtie2 (default parameters). The mapping reads were removed duplicated, and final bam files were converted to bigwig files. Active transcription regions are first defined by R package groHMM (version 1.16.0) with LtProbB = -400 and UTS = 5, obtaining 33454 in total. To minimize the false positive-effect, we next intersected those regions with known active genes(n=7232) identified by (Whyte et al., 2012) [16] and kept 3884 actively transcribed regions.

We then calculated the mean GRO-Seq signals inside each region using multiBigwigSummary with the argument --outRawCounts, and assigned it to a loop if it locates inside 100 kb up- and downstream of the loop. The active GRO-Seq signal of a loop is calculated by summing the mean signals of active regions within and dividing it by the length of the loop plus 200 kb. Loops were then sorted by their active GRO-Seq signals, and the top and bottom 200 are classified into group Top and Bottom (Related to Figure 4c).

PLAC-Seq Analysis

PLAC-Seq were download from the GEO datasets (additional file 2: Table S8), mapping to the mm10 with HiC-Pro (version 2.9.0), and the filtering reads were assigned to the MboI fragments. Processed PLAC-seq reads were then used as input into the Origami pipeline (version 1.1) to identify high confident bin to bin interaction pairs with parameter '--iterations=1000 --min-dist=5000 --max-dist=1000000 --no-interchromosomal --join-nearby-peaks=1000'. First, Origami estimates a probability score for each putative interaction by modeling the relationship between the number of PETs observed to support each interaction, linear genomic distance between interaction anchors, and read depth at the interaction anchors. Then, it uses a semi-Bayesian two-component mixture model to estimate the probability that a putative interaction corresponds to one of two groups: structured chromatin contacts (Group 1) or non-structured chromatin contacts and technical artifacts (Group 0). In the observed/expected analysis, the probability score is determined by iteratively estimating the group identity of each putative interaction, which reflects the probability that the interaction results come from Group 1, then a hypergeometric p-value is calculated based on the number of overlapping features in the data as compared to the number of overlapping features in the background dataset (follows the binomial distribution) with 10000 permutations. High-confidence interactions are then identified as the subset of putative interactions that are likely to represent structured chromatin contacts by

requiring a final origami score of at least 0.9 and a minimum PET count of 5. The distribution of the distance between two ends of PLAC-seq high confident loops was plotted as histogram between 0 kb and 300 kb inclusive (Related to Figure 4e).

PLAC-seq high-confidence interactions (N=44216) were classified according to the presence of at the anchors of each element as defined earlier. To avoid conflict, we adapted a hierarchy where anchors were considered first as promoters, then enhancers, then insulators. For subset analysis, the anchor of each loop was classified by overlapping with different regulatory features (such as an enhancer, promoter, terminator, and gene body) within 1 bp.

Loop Strength Distribution Analysis

To observe the genome-wide loop strength distribution, we defined the PETs linking the loop as loop strength. For loops called by hichipper or HiCCUPS (as described above), we calculated the numbers of loops with different strengths and plotted as histograms (Related to additional file 1: Fig. S3a).

Definition of regulatory regions

Promoters, terminator (TTS), enhancers, and super-enhancers are defined by the previous study.

Promoters

Promoters were defined as ± 1 kilobases from the transcription start site.

Terminators

Terminator were defined as ± 1 kilobases from the transcription termination site.

Insulators

Insulators were defined as the overlapping region of insulator neighborhood (available at: http://younglab.wi.mit.edu/insulatedneighborhoods.htm) and the CTCF chip-seq peak according to the (Weintraub et al., 2018) [22].

Super-enhancers

Oct4/Sox2/Nanog/Med1 super-enhancers and constituents were downloaded from (Whyte et al., 2013)[23].

Enhancers

The enhancers were downloaded from Whyte et al., 2013[23], and the coordinates were converted to mm10 with CrossMap (Version 0.2.8)[24].

Processed PLAC-seq reads were then used as input into the Origami pipeline to identify high confident bin to bin interaction pairs. The distribution of the distance between two ends of PLAC-seq high confident loops was plotted as histogram between 0 kb and 300 kb inclusive.

PLAC-seq high-confidence interactions were classified according to the presence of at the anchors of each element as defined earlier. To avoid conflict, we adapted a hierarchy where anchors were considered first as promoters, then enhancers, then insulators. For subset analysis, the anchor of each loop was classified by overlapping with different regulatory features (such as an enhancer, promoter, terminator, and gene body) within 1 bp.

Data sources

All the sequencing datasets generated in this study, including Hi-C, HiChIP, Ocean-C, RNA-seq, ChIP-seq, ATAC-seq, and 4C data, have been deposited in GEO with accession GSE145874.

STATISTICAL ANALYSES

At least two biological replicates were used for high-throughput experiments, the other experiments were repeated at least three times, and one representative plot, including error bars and N numbers, is shown in figures. All statistical tests were executed in R (http://www.r-project.org/). For the boxplots presented in the figures, centerline shows the median of each dataset, while the top and bottom of the box

represent the 75th and 25th percentiles, and 1.5 times the interquartile range (whiskers), one-sided t-tests were performed to compare the values between two groups for the data in the panels, with $p < 0.001$ considered to be statistically significant. Significant differences at center points between interaction meta-plots were performed using a Wilcoxon signed-rank test, as described in the figure legends.

Software packages used:

- cutadapt v1.15 (https://cutadapt.readthedocs.io/en/stable/)
- trim_galore v0.5.0 (https://www.bioinformatics.babraham.ac.uk/projects/trim_galore/)
- FastQC v0.11.8 (https://www.bioinformatics.babraham.ac.uk/projects/fastqc/)
- ChIA-PET2 v0.9.3 (trimLinker program: https://github.com/GuipengLi/ChIA-PET2)
- Bowtie2 v2.3. 5 (http://bowtie-bio.sourceforge.net/bowtie2/index.shtml)
- samtools v1.9 (https://github.com/samtools/samtools)
- BEDTools v2.26.0 (https://bedtools.readthedocs.io/)
- UCSC Genome Browser (https://genome.ucsc.edu/index.html)
- HiC-Pro v2.9.0 (https://github.com/nservant/HiC-Pro)
- HiCPlotter v0.7.1 (https://github.com/kcakdemir/HiCPlotter)
- Juicer and JuicerBox v1.9.9 on Linux (https://github.com/theaidenlab/juicebox/wiki/Download)
- deeptools v3.3.0 (https://deeptools.readthedocs.io/en/develop/)
- Bioconductor v3.6 (DESeq2, edgeR, groHMM and HiTC packages: https://www.bioconductor.org/)
- R v3.5.0 (RColorBrewer, ggplot2 packages: https://cran.r-project.org/)
- HICUP v0.7.0 (http://www.bioinformatics.babraham.ac.uk/projects/hicup/)
- Python v2.7 (pysam (>=0.8.3), bx-python (>=0.5.0), numpy (>=1.8.2), and scipy (>=0.15.1) libraries: https://www.python.org/)

- hiclib library (https://bitbucket.org/mirnylab/hiclib)
- MACS v1.4.2 and v2.0.10 (https://github.com/taoliu/MACS/)
- HTseq v0.11 (https://htseq.readthedocs.io/)
- Perl v5.18.2 (http://www.perl.org)
- UCSC utilities (http://hgdownload.soe.ucsc.edu/admin/exe/)
- fastq-multx v1.3.1 (https://github.com/brwnj/fastq-multx)
- pyicos v2.0.7 (https://bitbucket.org/regulatorygenomicsupf/pyicoteo/src/pyicoteo/#markdown-header-pyicos)
- DANPOS2 v2.2.2 (https://sites.google.com/site/danposdoc/)
- CrossMap v0.2.8 (http://crossmap.sourceforge.net/)

Reference

1. Natsume T, Kiyomitsu T, Saga Y, Kanemaki MT: Rapid Protein Depletion in Human Cells by Auxin-Inducible Degron Tagging with Short Homology Donors. *Cell Rep* 2016, 15:210-218.
2. Cong L, Ran FA, Cox D, Lin SL, Barretto R, Habib N, Hsu PD, Wu XB, Jiang WY, Marraffini LA, Zhang F: Multiplex Genome Engineering Using CRISPR/Cas Systems. *Science* 2013, 339:819-823.
3. Jain S, Ba Z, Zhang Y, Dai HQ, Alt FW: CTCF-Binding Elements Mediate Accessibility of RAG Substrates During Chromatin Scanning. *Cell* 2018, 174:102-116 e114.
4. Arrigoni L, Al-Hasani H, Ramirez F, Panzeri I, Ryan DP, Santacruz D, Kress N, Pospisilik JA, Bonisch U, Manke T: RELACS nuclei barcoding enables high-throughput ChIP-seq. *Commun Biol* 2018, 1:214.
5. Corces MR, Trevino AE, Hamilton EG, Greenside PG, Sinnott-Armstrong NA, Vesuna S, Satpathy AT, Rubin AJ, Montine KS, Wu B, et al: An improved ATAC-seq protocol reduces background and enables interrogation of frozen tissues. *Nat Methods* 2017, 14:959-962.
6. Rao SSP, Huntley MH, Durand NC, Stamenova EK, Bochkov ID, Robinson

JT, Sanborn AL, Machol I, Omer AD, Lander ES, Aiden EL: A 3D Map of the Human Genome at Kilobase Resolution Reveals Principles of Chromatin Looping. *Cell* 2014, 159:1665-1680.

7. Lajoie BR, Dekker J, Kaplan N: The Hitchhiker's guide to Hi-C analysis: practical guidelines. *Methods* 2015, 72:65-75.

8. Nora EP, Goloborodko A, Valton AL, Gibcus JH, Uebersohn A, Abdennur N, Dekker J, Mirny LA, Bruneau BG: Targeted Degradation of CTCF Decouples Local Insulation of Chromosome Domains from Genomic Compartmentalization. *Cell* 2017, 169:930-944 e922.

9. Durand NC, Shamim MS, Machol I, Rao SS, Huntley MH, Lander ES, Aiden EL: Juicer Provides a One-Click System for Analyzing Loop-Resolution Hi-C Experiments. *Cell Syst* 2016, 3:95-98.

10. Vian L, Pekowska A, Rao SSP, Kieffer-Kwon KR, Jung S, Baranello L, Huang SC, El Khattabi L, Dose M, Pruett N, et al: The Energetics and Physiological Impact of Cohesin Extrusion. *Cell* 2018, 173:1165-1178 e1120.

11. Lareau CA, Aryee MJ: hichipper: a preprocessing pipeline for calling DNA loops from HiChIP data. *Nat Methods* 2018, 15:155-156.

12. Pertea M, Kim D, Pertea GM, Leek JT, Salzberg SL: Transcript-level expression analysis of RNA-seq experiments with HISAT, StringTie and Ballgown. *Nat Protoc* 2016, 11:1650-1667.

13. Love MI, Huber W, Anders S: Moderated estimation of fold change and dispersion for RNA-seq data with DESeq2. *Genome Biol* 2014, 15:550.

14. Heinz S, Benner C, Spann N, Bertolino E, Lin YC, Laslo P, Cheng JX, Murre C, Singh H, Glass CK: Simple combinations of lineage-determining transcription factors prime cis-regulatory elements required for macrophage and B cell identities. *Mol Cell* 2010, 38:576-589.

15. Bonev B, Mendelson Cohen N, Szabo Q, Fritsch L, Papadopoulos GL, Lubling Y, Xu X, Lv X, Hugnot JP, Tanay A, Cavalli G: Multiscale 3D Genome Rewiring during Mouse Neural Development. *Cell* 2017, 171:557-572 e524.

16. Whyte WA, Bilodeau S, Orlando DA, Hoke HA, Frampton GM, Foster CT,

Cowley SM, Young RA: Enhancer decommissioning by LSD1 during embryonic stem cell differentiation. *Nature* 2012, 482:221-225.

17. Ramirez F, Ryan DP, Gruning B, Bhardwaj V, Kilpert F, Richter AS, Heyne S, Dundar F, Manke T: deepTools2: a next generation web server for deep-sequencing data analysis. *Nucleic Acids Res* 2016, 44:W160-165.
18. Durand NC, Robinson JT, Shamim MS, Machol I, Mesirov JP, Lander ES, Aiden EL: Juicebox Provides a Visualization System for Hi-C Contact Maps with Unlimited Zoom. *Cell Syst* 2016, 3:99-101.
19. Li GP, Chen Y, Snyder MP, Zhang MQ: ChIA-PET2: a versatile and flexible pipeline for ChIA-PET data analysis. *Nucleic Acids Research* 2017, 45.
20. Althammer S, Gonzalez-Vallinas J, Ballare C, Beato M, Eyras E: Pyicos: a versatile toolkit for the analysis of high-throughput sequencing data. *Bioinformatics* 2011, 27:3333-3340.
21. Chen K, Xi Y, Pan X, Li Z, Kaestner K, Tyler J, Dent S, He X, Li W: DANPOS: dynamic analysis of nucleosome position and occupancy by sequencing. *Genome Res* 2013, 23:341-351.
22. Weintraub AS, Li CH, Zamudio AV, Sigova AA, Hannett NM, Day DS, Abraham BJ, Cohen MA, Nabet B, Buckley DL, et al: YY1 Is a Structural Regulator of Enhancer-Promoter Loops. *Cell* 2018, 171:1573-1588 e1528.
23. Whyte WA, Orlando DA, Hnisz D, Abraham BJ, Lin CY, Kagey MH, Rahl PB, Lee TI, Young RA: Master transcription factors and mediator establish super-enhancers at key cell identity genes. *Cell* 2013, 153:307-319.
24. Zhao H, Sun Z, Wang J, Huang H, Kocher JP, Wang L: CrossMap: a versatile tool for coordinate conversion between genome assemblies. *Bioinformatics* 2014, 30:1006-1007.
