# Supplementary material for: Genome-wide analyses of chromatin interactions after the loss of Pol I, Pol II, and Pol III
Source: Genome Biol. 2020 Jul 2;21:158. doi: 10.1186/s13059-020-02067-3 (PMC7331254; doi:10.1186/s13059-020-02067-3)
Supplement: Supplementary file 4 — Additional file 4. Review history. [file 13059_2020_2067_MOESM4_ESM.docx]

Review History

**First round of review**

**Reviewer 1**

**Are you able to assess all statistics in the manuscript, including the appropriateness of statistical tests used?**

Yes, and I have assessed the statistics in my report.

**Comments to author:**

The suggestion of a scale dependent effect (or lack of effect) to chromatin structure upon depletion of RNA polymerases is interesting. Though this is not not a groundbreaking discovery, this does help to shed some light on potentially conflicting results published previously. I find the time-dependent effect of continued Pol II degradation, i.e. comparing 1 hr to 6 hr, especially interesting as this hints that the small-scale effects of Pol II could be dynamic (maybe via torsional stresses/unwinding) and required for maintenance of the proper, most efficient compartmentalisation of chromatin subtypes (e.g. putting enhancers nearer to target promoters).

Overall, the comprehension of this manuscript is somewhat hampered by the quality of the English, so this needs significant attention if there is a re-submission. Although I may point out some particular parts of the manuscript that are unclear, this is not to be taken as an exhaustive list; there are numerous errors and the whole document needs going through carefully. I also suggest that this long manuscript could be made clearer and more concise overall (for example, many details of the degron system and BAT Hi-C would be better confined to Methods).

The manuscript, as presented, has a potentially large problem concerning the use of only the untreated cells as a control for some analyses, rather than also using wild type cells. This is because the PolII binding, as illustrated by the CHIP-seq stack plots in Figure 1D, appears to be substantially reduced by the GFP tag alone; as is obvious by the lack of a vertical black line at zero (I assume that there is not a problem with the ChIP-seq scaling/normalisation). Analyses that contend no difference between treated and untreated ought to also check to make sure that the apparent Pol vs Pol-GPF binding differences have no effect, i.e. by comparing to the wild type. It is possible to have no detectable difference between lower (+GFP) and zero Pol binding but some difference between full/normal binding and low/zero binding. Some analyses are presented to support the notion that "mAID-GFP fusion supports the essential functions of RNA polymerases."; this is fairly well founded. However, these are separate measures from those that monitor chromatin 3D structure, which ought to be addressed directly. This problem could be easy to address if the authors have data for the wild type and can add-in analysis of the wild type to show that the situation with and without GFP tags is the same in the larger-scale (compartment and TAD) analyses.

Specific smaller issues:

I feel the statement "The relationship between transcription and the 3D genome organization is one of the most important questions in molecular biology" is too general and overly strong; we don't really know how important it is, though this perhaps makes it an interesting point of investigation.

Overall the authors should take care with the use of the word "global". It is fine to talk about global Cohesin binding when referring to genome-wide binding. However, the word "global" is sometimes mixed-up with "large-scale"; for the most part I think the authors mean large-scale (>25 kb) not global. For example this study suggests the PolII affects chromatin structure, to some degree, at a small-scale across the whole genome (i.e. globally), but not at a large-scale: globality and scale are separate concepts.

The phrase "identified Pol II bound but CTCF and Cohesin unbound loops" should be changed to something like "identified loops with bound Pol II but without cohesin or CTCF"

Overall the authors should make it clearer (at an early stage of the manuscript) what they mean by direct and indirect influence of RNA-pols upon chromatin structure.

I feel the authors should be more precise than saying RNA-pol loss has a "mild" effect: What kind of effect at what scale? In general the use of the words "mild" and "mildly" should be substituted with more precise language throughout the whole manuscript.

The phrase "transcription activities correlate with more DNA interactions in development and diseases" is imprecise; I assume _more_ transcription correlates with _more_ DNA interactions.

"CTCF loop domains usually enrich active transcription"; I assume this should be something like "CTCF loop domains are usually _enriched_ with active transcription", i.e. there is not a specific causal relation.

In discussions of Drosophila it ought to be made clear that the Drosophila genome has a mich higher gene density than the mouse genome, which likely affects chromosome 3D organisation.

In the Background section it is stated that transcription does affect cohesin binding at CTCF but that it does not affect TADs. I feel this section could be more nuanced and refer to scale dependences; it is possible to change cohesin positioning in mostly small, gene-scale, inter-TAD gaps but not have a notable effect at a large scale that would be seen with Hi-C binned at 25 kb.

It is unclear what the authors mean when they refer to "transcription-associated proteins". Are they referring to chromatin remodellers and/or transcription factors?

The word phenotype is not correctly used in the phrase "The unchanged phenotype after transcription inhibition"; phenotype refers to an organism's observable characteristics.

In "It is immature to conclude", the authors probably mean "premature", not "immature".

"RNA polymerases synthesize RNA, forming DNA, RNA, and protein ternary complexes in the nucleus" should be re-written; this reads as if there are separate DNA, RNA and protein complexes, not complexes of DNA, RNA and protein combined.

Though it is perhaps obvious, I would nonetheless state that the largest subunits of the RNA-pols are essential.

The authors should consider moving away from saying that the RNA-pols have a marginal role, rather that they have a local, small-scale role.

In "CTCF unbound loops, and they are largely resistant to transcription inhibition"; I would say they are largely _unchanged_ after inhibition.

The lack of a small-scale effect upon degrading Po lI and Pol II (unlike Pol II) deserves some mechanistic speculation.

In "long-time depletion of Pol II" should be "longer-term depletion of Pol II (>=6hr)".

it is not clear what the authors mean by "accurately" in "RNA polymerases could be degraded rapidly and

accurately after the addition of auxin".

In Figure 2 A,B,C,F how do I tell whether the A/B compartmental contact frequencies are significantly different or not?

Stacked ChIP-seq plots (Fig 1D, 6B, 6C), would benefit from an overall co-localisation or peak size statistic to aid comparison.

The heading "Pol I, Pol II and Pol III loss mildly alter local chromatin interactions" should be rephrased.

The sentence "Our RNA polymerases degron system can degrade Pol II within 1hr (Fig. 1b), which has the unique advance to separate the impact of transcription from the presence of RNA polymerases" requires further explanation and rephrasing; if PolII is degraded one would think transcription will stop because the protein is removed, so why is transcription thereby separated from Pol II presence? Do the authors mean to distinguish the pool of unbound Pol II from the bound Pol II involved in ongoing transcription?

**Reviewer 2**

**Are you able to assess all statistics in the manuscript, including the appropriateness of statistical tests used?**

Yes, and I have assessed the statistics in my report.

**Comments to author:**

How chromatin architecture affects gene expression and whether changes in the spatial organization of the human genome can influence cell fate are important questions, which have not been fully address yet. Specifically, what, if any, is the role of transcription in the topological organization of the genome and whether changes in regulatory loops are cause or consequence of transcriptional activity remains unclear.

In this manuscript the authors use the auxin degron system to deplete Pol I, Pol II and Pol III and show that this has very little effect on global chromatin architecture, while selected regions are more strongly affected. One of the more interesting and novel findings of the paper in my opinion is that Pol II is not required for the reestablishment of chromatin structure after mitosis and long-term depletion of Pol II leads to disruption of chromatin accessibility and cohesin binding, potentially leading to an indirect effect on 3D genome architecture.

In general, I believe that these findings are relatively novel and will be of interest to the community. They highlight the fact that genome architecture is more robust than previously anticipated and leave open the possibility that changes in conformation that have been observed for example in development or differentiation happen before, not after gene activation. However, some very important points need to be addressed before the manuscript is suitable for publication in Genome Biology. In addition, several important citations regarding the role of transcription in 3D genome architecture should be included and discussed properly. For example, the authors cite Hug et al., Cell 2017 (citation 12 in the manuscript) for the prediction that transcription influences genome architecture, but do not mention or discuss the experiments inhibiting transcription, which largely show very little effect on 3D topology. Additionally, Bonev et al., Cell 2017 showed that transcriptional activation at the endogenous loci also does not lead to local changes in 3D genome architecture, arguing that transcription alone is not sufficient to alter topology.

Major:

1. Location of the degron tag and appropriate controls

A major potential issue in the manuscript is the location of the degron tag at the C-terminus (specifically talking about Pol II here). CTD is the protein domain involved in transcriptional initiation, interactions with Mediator and many regulatory process converge on it, therefore the authors need to show convincingly that tagging the protein there does not majorly interfere with its function. While the majority of the controls shown make use of + vs - auxin conditions, the authors need to compare the tagged version (- auxin) with untagged conditions. To avoid creating new datasets a comparison between Pol II and Pol I/ III tagged (no auxin conditions) would also be acceptable. It is essential to know the magnitude of changes in gene expression (RNA-seq), Pol II binding and 3D topology among these conditions, before deciding whether the -auxin samples are an appropriate control.

2. Figure 2 cross-comparisons and CTCF vs transcription.

It is unclear from the text or the figure legends if features (compartments, boundaries, TADs) were called separately in + / - auxin condition or the same set of features, called in the control (-auxin) condition, were used. The second approach would be more appropriate here in my opinion. Furthermore, the representation of TAD boundaries as function of distance plot is not really informative, the authors should plot the insulation score on boundaries identified in the control conditions instead. Finally and most importantly, despite stressing (accurately) in the introduction that the effect of CTCF and transcription is rarely disentangled, the authors then do not do any comparisons between CTCF vs non-CTCF based TADs and insulation. To conclusively show that there is no transcription-associated effect, the authors should: 1) examine insulation score at CTCF-bound vs CTCF-less promoters (active vs repressed) independent of TAD boundaries; 2) rank CTCF-less gene promoters based on gene expression and Pol II binding in control conditions and examine the insulation factor and 3) look at compartment domains vs loop domains as defined by Rowley et al. 2017 and Rao et al., 2014. They should also use TAD boundaries defined with high-resolution Hi-C where possible, for example Bonev et al., Cell 2017.

3. Figure 3: promoter loop comparison

There is plenty of evidence that active promoters engage in long-range interactions (Schoenfelder et al., 2015; Bonev et al., 2017) but these interactions frequently appear in pairs and not in clusters (Olivares-Chauvet et al., Nature 2016). The authors should look at aggregate contact strength enrichment (PE-scan/APA, etc) between pairs of promoters of high/lowly expressed genes (based on GRO-seq for example) ; bound/not bound by CTCF and across multiple distances (intra vs interTAD; <2MB vs >2MB). Further to these, the analysis for the H3K27ac HiChIP and the Ocean-C should also quantify interactions strength at pairs of promoters, not just based on loop identification (based on Figure5A even loops classified as no/no still have weak CTCF binding). Analogously, for Pol I and Pol III, they should focus on genes known to be regulated by these polymerases (ribosomal, tRNAs, etc) in addition to the global comparison.

4. Figure 6: effect on cohesin/CTCF vs global compaction

After 6hr the authors report a decrease in accessibility and cohesin binding across promoters, enhancers and CTCF sites. Is that due to a general chromatin compaction or a specific effect at CTCF/cohesin sites? One can potentially address that by comparing the accessibility at CTCF/cohesin bound vs not bound ATAC-peaks (promoters vs distal elements separately) or using Hi-C data (cis-decay profile in A- vs B- TADs).

5. Effect on gene loops

Several studies based on micro-C (Hsieh et al., biorxiv) or Hi-C (Boxer et al., Molecular Cell 2020) suggest that particular types of loops between promoters and the gene body of highly transcribed genes are associated with transcription and that inhibition of transcription leads to their disruption (Hsieh et al., biorxiv). The authors should also examine these type of loops upon Pol II degradation with their high-resolution HiChiP, as this could be important and novel conceptual finding.

Minor

-	It is very surprising to see more than 3000 peaks for Pol I, can this be cross-validated (for example comparing it with published untagged Pol I ChIP). Analogous for Pol III.

-	The Hi-C datasets seem to have an abnormally high trans/cis ratio, is that typical for BAT Hi-C?

-	Clustering at figure S2D should be done using individual replicates to increase robustness

-	TADs defined by arrowhead tend to be mostly loop domains and are fewer than described in other papers from the same cell type. The authors should verify those finding using other approaches (insulation score, etc) or publicaly available TAD coordinates (Bonev et al., Cell 2017)

-	The data in Figure S2F is important but it is unclear which degron is referred to. Data for all of the +/auxin combinations should be shown here. Also S2F is not observed/expected ratios as in the text.

-	Figure 4D: please add statistics: is this enrichment higher/lower than expected based on the frequency and length of the genomic feature?

-	Contact enrichment (obs/exp) should be also quantified at promoter-promoter and promoter-enhancer loops, not just mean distance

-	p14/ lines 48-51: unclear what is meant with this sentence

**Authors Response**

**Point-by-point responses to the reviewers’ comments:**

*The response summary of the major comments and our point by point responses are followed:*

1) Both reviewers 1 and 2 suggested the wild type controls of Pol II binding, 3D chromatin structures, and gene expression for our degron cells.

*Response: we show that the RNA polymerases chromatin-binding strength (NEW Fig. 1d, S1d-e), 3D chromatin structures (NEW Fig. S2d) and mRNA transcriptome (NEW Fig. S1c) for wild type and tagged RNA polymerases mES cells are highly correlated, indicating that we could use tagged cells (untreated) as controls for our downstream analyses.*

2) Reviewer 2 suggested the comparison of insulation scores between CTCF and non-CTCF TAD.

*Response: we compared the insulation scores for CTCF and non-CTCF TAD defined with high-resolution Hi-C from Bonev et al., Cell 2017 as suggested by reviewer 2. We show that there are no obvious changes in both the CTCF bound and unbound boundaries between untreated and auxin conditions for Pol I, Pol II or Pol III degron cells (NEW Fig. 2a-c, middle panel), while the insulation score shows dramatic decreases in the publicly available CTCF degradation Hi-C data sets (NEW Fig. 2e).*

3) Reviewer 2 suggested some data comparisons of the promoter-related insulation score, promoter pairs, and stripe signals.

*Response: we identified active_CTCF-bound, active_no CTCF, silent_CTCF-bound, and silent_no CTCF promoters, and used the promoters that are localized to loop anchors, which have more reliable insulation scores for the analyses. We then subgrouped the no CTCF promoters into high/medium/low groups based on gene expression with GRO-Seq data. We observed relatively no or few changes for the insulation scores of these promoters in Pol II degradation H3K27ac HiChIP and Ocean-C data sets (NEW Fig. 6d-e, S6d-e).*

*Our PE-SCAn plots between pairs of promoters across multiple distances (intra-TAD<2 MB, long-range>2 MB) (NEW Fig. 6a, S6a), a different expression level (high/medium/low) (NEW Fig. 6b, S6b), with CTCF binding and without CTCF binding (NEW Fig. 6c, S6c), collectively show that the distance and expression level modestly affect the active transcription-mediated promoter-promoter interactions in a CTCF-independent manner.*

*We show that the 45s rRNA locus does not show obvious changes after Pol I depletion, while it appears to have relatively modest effects on the chromatin interactions in mRNA and tRNA loci after the depletions of Pol II and Pol III (NEW Fig. 3f-h), respectively.*

*We performed stripe analyses with our H3K27ac HiChIP data sets at enhancer, promoter, and CTCF insulator regions and show that there are few or no differences of these stripe signals after Pol II degradation (NEW Fig. S6f).*

4) Reviewer 1 suggests substantial copy editing and more concise writing.

*Response: we carefully revised the entire manuscript to make it more understandable and concise.*

Reviewer #1:

The suggestion of a scale dependent effect (or lack of effect) to chromatin structure upon depletion of RNA polymerases is interesting. Though this is not a groundbreaking discovery, this does help to shed some light on potentially conflicting results published previously. I find the time-dependent effect of continued Pol II degradation, i.e. comparing 1 hr to 6 hr, especially interesting as this hints that the small-scale effects of Pol II could be dynamic (maybe via torsional stresses/unwinding) and required for maintenance of the proper, most efficient compartmentalization of chromatin subtypes (e.g. putting enhancers nearer to target promoters).

*Response: we appreciate that the reviewer finds that our time course chromatin structure analyses after the depletion of RNA polymerases are interesting. The reviewer’s comments for our time course results are inspiring.*

*For revision, we added “Furthermore, the Pol II degradation time course analyses also indicated that the small-scale effects of Pol II on chromatin structures could be dynamic and required for the maintenance of the proper, most efficient compartmentalization of chromatin substructures (such as, placing enhancers nearer to target promoters).” to the revised manuscript P16/line2.*

Overall, the comprehension of this manuscript is somewhat hampered by the quality of the English, so this needs significant attention if there is a re-submission. Although I may point out some particular parts of the manuscript that are unclear, this is not to be taken as an exhaustive list; there are numerous errors and the whole document needs going through carefully. I also suggest that this long manuscript could be made clearer and more concise overall (for example, many details of the degron system and BAT Hi-C would be better confined to Methods).

*Response: thanks so much for the reviewer’s help and hard work to make our manuscript clearer and more concise. We moved the details of the degron system and BAT Hi-C to the method section and carefully revised the entire manuscript to make it more understandable and concise.*

The manuscript, as presented, has a potentially large problem concerning the use of only the untreated cells as a control for some analyses, rather than also using wild type cells. This is because the Pol II binding, as illustrated by the CHIP-seq stack plots in Figure 1D, appears to be substantially reduced by the GFP tag alone; as is obvious by the lack of a vertical black line at zero (I assume that there is not a problem with the ChIP-seq scaling/normalization). Analyses that contend no difference between treated and untreated ought to also check to make sure that the apparent Pol vs Pol-GPF binding differences have no effect, i.e. by comparing to the wild type. It is possible to have no detectable difference between lower (+GFP) and zero Pol binding but some difference between full/normal binding and low/zero binding. Some analyses are presented to support the notion that "mAID-GFP fusion supports the essential functions of RNA polymerases."; this is fairly well founded. However, these are separate measures from those that monitor chromatin 3D structure, which ought to be addressed directly. This problem could be easy to address if the authors have data for the wild type and can add-in analysis of the wild type to show that the situation with and without GFP tags is the same in the larger-scale (compartment and TAD) analyses.

*Response: The heat map in the original Figure 1d cannot really reflect the binding differences between Pol and GFP-Pol, because they were generated with the different antibodies, different library preparation procedures, and sequencing depth. Especially, the Pol, and GFP-Pol ChIP were plotted under vast different sequencing depth in the original Figure 1d (the GFP-Pol sequencing around 7.6% the reads of the Pol ChIP). The Pol ChIP-Seq data was generated with endogenous antibodies with a regular ChIP-Seq library preparation method and sequencing around 316 million reads. The GFP-Pol ChIP-Seq was carried out with a modified method of the RELACS (restriction enzyme-based labeling of chromatin in situ) published previously to unbiasedly compare the Pol II binding between untreated and auxin treated conditions (Arrigoni et al., Communications Biology, 2018). The GFP-Pol ChIP-Seq experiment starts with the fragmentation of the chromatin with restrict enzyme AluI, ligate the fragmented DNA to different adapters to barcode untreated and auxin treated cells, then mix these cells to perform GFP chromatin immunoprecipitation, extract DNA from the ChIP preparations, amplify and sequencing the ChIP DNA for around 24 million reads. So, the Pol binding and GFP-Pol binding cannot be directly compared in the original Figure 1d. To directly compare the Pol binding with the GFP-Pol binding, we subsampled the Pol and GFP-Pol ChIP-Seq data sets into equal amounts and stacked these signals at the peaks of the Pol binding. The heat map illustrates in the revised Figure 1d shows that the Pol binding and GFP-Pol binding strength appears to be quite similar, suggesting that the GFP tagging does not affect Pol II binding. The scatter plot correlation analyses indicated that the ChIP-Seq signals for Pol and GFP-Pol are also highly correlated, as shown in Figure S1e in the revised manuscript (R2>0.91). Similarly, in the paper published by Nora et al., in 2017 where CTCF was investigated with the same degron system, they also showed there are no obvious CTCF chromatin binding differences between tagged/untagged CTCF cells (Figure S2a).*

*For revision, we added the detailed method for “-/+ auxin GFP ChIP-Seq” to the method section in the revised manuscript.*

*The reviewer suggested the comparison for chromatin structures between wild type and tagged cells. Insulation score and PC1 values (principal component) are critical parameters for the evaluation of TADs and compartments with Hi-C data set. We then performed clustering analyses for insulation score and PC1 values for the Hi-C data sets generated with wild type or tagged Pol I or Pol II or Pol III cells. The results in the revised Figure S2d show that they are highly correlated (Pearson's correlation coefficient>0.9), indicating that the 3D chromatin structures of our tagged cells are largely the same with the wild type cells. Furthermore, we also compared the global mRNA expression by RNA-Seq between wild type cells and tagged Pol II cells, as suggested by reviewer 2. The scatter plot and clustering analyses in the revised Figure S1c indicated that their transcriptome is also quite similar. Together, our revised results suggest that the tagged mESCs are mostly the same as the wild type mESCs in RNA polymerases chromatin-binding, mRNA transcriptome, and 3D chromatin structures.*

*For revision, we added the sentence “The RNA polymerases chromatin-binding affinity, mRNA transcriptome and 3D chromatin structures in the wild-type and tagged RNA polymerases cells are highly correlated (Figs. 1d, S1c, S1e, S2d), indicating that we could use tagged cells (untreated) as controls for our downstream analyses.” to the revised manuscript P6/line1.*

Specific smaller issues:

I feel the statement "The relationship between transcription and the 3D genome organization is one of the most important questions in molecular biology" is too general and overly strong; we don't really know how important it is, though this perhaps makes it an interesting point of investigation.

*For revision, we replaced this statement by “The relationship between transcription and the 3D chromatin structure is hotly debated and is an interesting topic to investigate.” in the revised manuscript P2/line1.*

Overall the authors should take care with the use of the word "global". It is fine to talk about global Cohesin binding when referring to genome-wide binding. However, the word "global" is sometimes mixed-up with "large-scale"; for the most part I think the authors mean large-scale (>25 kb) not global. For example this study suggests the Pol II affects chromatin structure, to some degree, at a small-scale across the whole genome (i.e. globally), but not at a large-scale: globality and scale are separate concepts.

*Response: We agree with the reviewer’s comments on “global” and “large-scale”. In the revised manuscript, we used “global” when referring to genome-wide; “large-scale” when referring to Compartments, TADs, or large loop domains (>250kb), “small-scale” when referring to the gene-associated chromatin structures. We also added these sentences in the revised manuscript method section.*

The phrase "identified Pol II bound but CTCF and Cohesin unbound loops" should be changed to something like "identified loops with bound Pol II but without cohesin or CTCF"

*For revision, we replaced the phrase by “loops with bound Pol II but without Cohesin or CTCF” in the revised manuscript.*

Overall the authors should make it clearer (at an early stage of the manuscript) what they mean by direct and indirect influence of RNA-pols upon chromatin structure.

*For revision, we added “RNA polymerases may involve in 3D chromatin organization directly by interacting with structural proteins or noncoding RNAs or indirectly through the downstream effects of transcription.” to the revised manuscript P4/line13 to make it more precise about the direct and indirect influence of RNA polymerases upon 3D chromatin structures.*

I feel the authors should be more precise than saying RNA-pol loss has a "mild" effect: What kind of effect at what scale? In general the use of the words "mild" and "mildly" should be substituted with more precise language throughout the whole manuscript.

*For revision, we substituted the words "mild" and "mildly" with “local, small-scale” or added more precise phrases with “mild” throughout the whole manuscript.*

The phrase "transcription activities correlate with more DNA interactions in development and diseases" is imprecise; I assume _more_ transcription correlates with _more_ DNA interactions.

*For revision, we replaced the statement by “Mounting evidence has shown that a high level of transcription activity is correlated with a higher frequency of DNA interactions in both development and diseases” in the revised manuscript P3/line2.*

"CTCF loop domains usually enrich active transcription"; I assume this should be something like "CTCF loop domains are usually _enriched_ with active transcription", i.e. there is not a specific causal relation.

*For revision, we replaced the statement by “CTCF loop domains are usually enriched with active transcription” in the revised manuscript P3/line7.*

In discussions of Drosophila it ought to be made clear that the Drosophila genome has a much higher gene density than the mouse genome, which likely affects chromosome 3D organization.

*For revision, we added this sentence, “As the D. melanogaster genome has a much higher gene density than the mammalian genome.” in the revised manuscript P3/line15.*

In the Background section, it is stated that transcription does affect cohesin binding at CTCF but that it does not affect TADs. I feel this section could be more nuanced and refer to scale dependences; it is possible to change cohesin positioning in mostly small, gene-scale, inter-TAD gaps but not have a notable effect at a large scale that would be seen with Hi-C binned at 25 kb.

*For revision, we added this sentence “It is also possible that transcription changes Cohesin occupancy on a mostly small, gene-scale, which may not have a notable effect on the large-scale chromatin structures that can be detected with the Hi-C method used on a large-scale.” in the revised manuscript P3/line28.*

It is unclear what the authors mean when they refer to "transcription-associated proteins". Are they referring to chromatin remodellers and/or transcription factors?

*Response: The "transcription-associated proteins" refer to proteins involved in the transcription process, such as transcription factors, cofactors, and chromatin regulators.*

*For revision, we replaced "transcription-associated proteins" with “Proteins involve in the transcription process” at P3/line 27 in the revised manuscript.*

The word phenotype is not correctly used in the phrase "The unchanged phenotype after transcription inhibition"; phenotype refers to an organism's observable characteristics.

*For revision, we replaced the word “phenotype” with “pattern” in the revised manuscript P4/line1.*

In "It is immature to conclude", the authors probably mean "premature", not "immature".

*For revision, we replaced "immature" with “premature” in the revised manuscript P4/line6.*

"RNA polymerases synthesize RNA, forming DNA, RNA, and protein ternary complexes in the nucleus" should be re-written; this reads as if there are separate DNA, RNA and protein complexes, not complexes of DNA, RNA and protein combined.

*For revision, we deleted this sentence for the sake of concise.*

Though it is perhaps obvious, I would nonetheless state that the largest subunits of the RNA-pols are essential.

*For revision, we replaced the sentence by “we specifically degraded the largest essential subunits of Pol I, Pol II, and Pol III in murine embryonic stem cells” in the revised manuscript P4/line19.*

The authors should consider moving away from saying that the RNA-pols have a marginal role, rather that they have a local, small-scale role.

*For revision, we removed the claim “RNA polymerases have a marginal role” and changed it into “they have a local, small-scale role” in the revised manuscript.*

In "CTCF unbound loops, and they are largely resistant to transcription inhibition"; I would say they are largely _unchanged_ after inhibition.

*For revision, we replaced the phrase "largely resistant to transcription inhibition" with “largely unchanged after transcription inhibition” in the revised manuscript.*

The lack of a small-scale effect upon degrading Po I and Pol III (unlike Pol II) deserves some mechanistic speculation.

*Response: We observed small-scale effects upon the degradation of Pol II, and also observed small-scale effects upon the selected regions after the degradation of Pol I or Pol III (Figure 3a, 3c, 3d, 3h). We now make it clear in the revised manuscript. We also added some mechanistic speculations for the small-scale effects in the discussion part, as suggested by the reviewer in the revised manuscript P16/line 2.*

In "long-time depletion of Pol II" should be "longer-term depletion of Pol II (>=6hr)".

*For revision, we have replaced "long-time depletion of Pol II" with "the longer-term depletion of Pol II (6 hr)" in the revised manuscript P5/line 1.*

it is not clear what the authors mean by "accurately" in "RNA polymerases could be degraded rapidly and accurately after the addition of auxin".

*Response: Transcription inhibitors are wildly used, but they have difficulties in distinguishing different RNA polymerases. Here we add GFP-mAID tag into the C-terminal of Pol I, Pol II, and Pol III largest subunit, respectively, which could specifically degrade the tagged RNA polymerases with the auxin-induced degradation methods.*

*For revision, we replaced the sentence with "rapid degradation can be achieved for a specific RNA polymerase after the addition of auxin " in the revised manuscript P5/line24.*

In Figure 2 A,B,C,F how do I tell whether the A/B compartmental contact frequencies are significantly different or not?

*Response: We compared the A/B compartmental contact frequencies by presenting a violin plot to show the 1st eigenvalues (EV), which are calculated using the cis-interactions of untreated/+auxin Pol-GFP cells, as it is difficult to appreciate the changes using previous saddle plots visually. This figure has added as Figure S2h. It indicates that there is no significant difference in the eigenvalues, suggesting no substantial change of the A-, B-type compartmentalization after RNA polymerases depletion. In the violin plots, the horizontal bands represent the median, the error bars show 1.5+/- IQR values, and the outer shapes describe the density of the data point. The figures represent data generated from the pooled Hi-C datasets with two biological replicates. P-value: Wilcoxon rank-sum test. We have also kept saddle plots to quantify better the compartment strength (Figure 2a-c, 2f).*

Stacked ChIP-seq plots (Fig 1D, 6B, 6C), would benefit from an overall co-localisation or peak size statistic to aid comparison.

*For revision, we added a Venn diagram showing the existence of common and specific peaks among Pol I, Pol II, and Pol III, and each of them has different peak size distribution. These two figures were added in Figure S1d in the revised manuscript. Regarding Figure 6b, 6c, we performed meta-analyses of ATAC-Seq (6b) and Coheisn ChIP-Seq signals (6c) at the promoter, enhancer, and insulator regions. We now labeled them clear in the revised manuscript, and the definitions of these regions are in the method sections of the revised manuscript.*

The heading "Pol I, Pol II and Pol III loss mildly alter local chromatin interactions" should be rephrased.

*For revision, we replaced this sentence with " Pol I, Pol II and Pol III loss altered local, small-scale chromatin interactions" in the revised manuscript.*

The sentence "Our RNA polymerases degron system can degrade Pol II within 1hr (Fig. 1b), which has the unique advance to separate the impact of transcription from the presence of RNA polymerases" requires further explanation and rephrasing; if Pol II is degraded one would think transcription will stop because the protein is removed, so why is transcription thereby separated from Pol II presence? Do the authors mean to distinguish the pool of unbound Pol II from the bound Pol II involved in ongoing transcription?

*Response: “to separate the impact of transcription from the presence of RNA polymerases” We mean that we can distinguish between the RNA polymerase proteins and the transcription states, as our Degron system depletes Pol II (Rpb1) proteins within 1hr and may have limited impacts on the transcription states, including other transcription apparatuses and active transcription-associated chromatin environments.*

*For revision, we replaced this sentence with “Our RNA polymerases degron system degraded Pol II within 1 hr (Fig. 1b), which provided the unique ability to separate the impact of transcription states (including other transcription apparatuses and active transcription-associated chromatin environments) from the presence of RNA polymerase proteins per se” in the revised manuscript P11/line27.*

Reviewer #2:

How chromatin architecture affects gene expression and whether changes in the spatial organization of the human genome can influence cell fate are important questions, which have not been fully address yet. Specifically, what, if any, is the role of transcription in the topological organization of the genome and whether changes in regulatory loops are cause or consequence of transcriptional activity remains unclear.

In this manuscript the authors use the auxin degron system to deplete Pol I, Pol II and Pol III and show that this has very little effect on global chromatin architecture, while selected regions are more strongly affected. One of the more interesting and novel findings of the paper in my opinion is that Pol II is not required for the reestablishment of chromatin structure after mitosis and long-term depletion of Pol II leads to disruption of chromatin accessibility and cohesin binding, potentially leading to an indirect effect on 3D genome architecture.

*Response: We agree with the reviewer that the relationship between transcription and the 3D topological organization of the genome has not been fully address yet, even though it has been investigated in numerous studies with various biological systems. We appreciate that the reviewer stated that our mitotic exit and time course Pol II degradation results are interesting and novel.*

In general, I believe that these findings are relatively novel and will be of interest to the community. They highlight the fact that genome architecture is more robust than previously anticipated and leave open the possibility that changes in conformation that have been observed for example in development or differentiation happen before, not after gene activation. However, some very important points need to be addressed before the manuscript is suitable for publication in Genome Biology.

In addition, several important citations regarding the role of transcription in 3D genome architecture should be included and discussed properly. For example, the authors cite Hug et al., Cell 2017 (citation 12 in the manuscript) for the prediction that transcription influences genome architecture, but do not mention or discuss the experiments inhibiting transcription, which largely show very little effect on 3D topology. Additionally, Bonev et al., Cell 2017 showed that transcriptional activation at the endogenous loci also does not lead to local changes in 3D genome architecture, arguing that transcription alone is not sufficient to alter topology.

*Response: We agree with the reviewer that the comments in the Hug et al., Cell 2017 and Bonev et al., Cell 2017 are very important.*

*For revision, we now add, “inhibiting D. melanogaster transcription significantly alters chromatin interactions both within and between domains, but has very little effect on the 3D topology of TADs” in the revised manuscript P3/line16. We add, “Additionally, Bonev et al., Cell 2017 showed that transcriptional activation at the endogenous loci does not lead to local changes in 3D genome structures, arguing that transcription alone is not sufficient to alter genome topology.” in the revised manuscript P17/line7.*

Major:

1. Location of the degron tag and appropriate controls

A major potential issue in the manuscript is the location of the degron tag at the C-terminus (specifically talking about Pol II here). CTD is the protein domain involved in transcriptional initiation, interactions with Mediator and many regulatory process converge on it, therefore the authors need to show convincingly that tagging the protein there does not majorly interfere with its function. While the majority of the controls shown make use of + vs - auxin conditions, the authors need to compare the tagged version (- auxin) with untagged conditions. To avoid creating new datasets a comparison between Pol II and Pol I/ III tagged (no auxin conditions) would also be acceptable. It is essential to know the magnitude of changes in gene expression (RNA-seq), Pol II binding and 3D topology among these conditions, before deciding whether the -auxin samples are an appropriate control.

*Response: We agree with the reviewer that we need to evaluate the gene expression (RNA-Seq), Pol II binding, and 3D topology between tagged and untagged conditions before using the tagged (untreated) samples as our controls.*

*We compared gene expression derived from RNA-seq-based analyses between wild type and tagged Pol II mES cells. The pairwise scatter plot and hierarchical clustering in the revised Figure S1c of these datasets highlight their similarities, with a high Pearson's correlation coefficient (R2>0.96).*

*To directly compare the Pol binding with the GFP-Pol binding, we subsampled the ChIP-Seq datasets into equal amounts and stacked these signals at the peaks of the Pol binding. The heat map in the revised Figure 1d indicates that the Pol binding and GFP-Pol binding strength appears to be quite similar, suggesting that the GFP-degron tagging does not affect Pol binding. The scatter plot correlation analyses indicated that the ChIP-Seq signals for Pol and GFP-Pol are highly correlated, as shown in Figure S1e in the revised manuscript (R2>0.91). Consistently, in the paper published by Nora et al., 2017 where CTCF was investigated with the same degron system, they also showed there are no obvious CTCF chromatin binding differences between tagged/untagged CTCF cells (Figure S2A).*

*We also compared 3D topology of the genome at different scales, such as TAD and compartment between wild type and GFP-degron tagged cells. Insulation score and PC1 values (principal component) are critical features for examining the strength of TAD boundaries and compartments. Hierarchical clustering based on the insulation score and PC1 values from fourteen Hi-C datasets demonstrated that those mES cells upon different engineering are still mostly the same (Pearson's correlation coefficient>0.9). Together, our revised results demonstrate that the tagged mESCs are largely the same as the wild type mES cells in RNA polymerases chromatin binding, mRNA transcriptome, and 3D chromatin structures, suggesting that untagged (-auxin) samples are appropriate controls for our perturbation analyses.*

*For revision, we add the sentence “The RNA polymerases chromatin- binding affinity, mRNA transcriptome and 3D chromatin structures in the wild-type and tagged RNA polymerases cells are highly correlated (Figs. 1d, S1c, S1e, S2d), indicating that we could use tagged cells (untreated) as controls for our downstream analyses.” to the revised manuscript P6/line1.*

2. Figure 2 cross-comparisons and CTCF vs transcription.

It is unclear from the text or the figure legends if features (compartments, boundaries, TADs) were called separately in + / - auxin condition or the same set of features, called in the control (-auxin) condition, were used. The second approach would be more appropriate here in my opinion. Furthermore, the representation of TAD boundaries as function of distance plot is not really informative, the authors should plot the insulation score on boundaries identified in the control conditions instead. Finally and most importantly, despite stressing (accurately) in the introduction that the effect of CTCF and transcription is rarely disentangled, the authors then do not do any comparisons between CTCF vs non-CTCF based TADs and insulation. To conclusively show that there is no transcription-associated effect, the authors should: 1) examine insulation score at CTCF-bound vs CTCF-less promoters (active vs repressed) independent of TAD boundaries; 2) rank CTCF-less gene promoters based on gene expression and Pol II binding in control conditions and examine the insulation factor and 3) look at compartment domains vs loop domains as defined by Rowley et al. 2017 and Rao et al., 2014. They should also use TAD boundaries defined with high-resolution Hi-C where possible, for example Bonev et al., Cell 2017.

*Response: We call features (compartments, TADs) for +/- auxin conditions separately and used the union set of features in two conditions to avoid bias on either condition.*

*For revision, we add the sentence, “In this study, we called features (compartments, TADs) for +/- auxin condition separately, and used the union set of features in two conditions" in the revised manuscript method section of Hi-C, HiChIP and Ocean-C Data Analyses.*

*We agree with the reviewer that the distance plot of the TAD boundary is not informative. We compared the insulation score between CTCF vs non-CTCF based TAD boundaries defined with high-resolution Hi-C from Bonev et al., Cell 2017 as suggested by the reviewer. To do this, we used the TAD boundaries defined in Bonev et al., Cell 2017, and classified them into CTCF bound and non-CTCF boundaries. The results in the revised Figure 2 show that there are no obvious changes between untreated and auxin conditions after RNA polymerase depletions, while the insulation score dramatic decreases with the publicly available CTCF degradation Hi-C dataset.*

*For revision, we added the sentences, “We also performed insulation score analyses with the TAD boundaries defined through high-resolution Hi-C analyses published previously. The results showed that there were no obvious changes in either the CTCF-bound or CTCF-unbound boundaries under the untreated and auxin conditions used for the Pol I, Pol II or Pol III degron cells (Fig. 2a-c), while the insulation score showed dramatic decreases in the publicly available CTCF degradation Hi-C data sets (Fig. 2e).” to the revised manuscript P8/line12.*

*To conclusively show the transcription-associated effects, we carried out the insulation score analyses with different types of promoters, which was suggested by the reviewer, with our high-resolution HiChIP and Ocean-C data sets. As our HiChIP and Ocean-C data sets are not deep enough to allow us to examine the insulation scores for all promoters, we only used the promoters that are localized to loop anchors, which have more reliable insulation scores for the analyses. We then subgrouped the CTCF-bound vs CTCF-less promoters based on whether there is a CTCF binding peak within 5 kb or not. The genes with histone markers H3K4me3 and H3K79me2 are defined as active genes, and the genes without histone markers H3K4me3, H3K79me2, and H3K27me3 are defined as silent genes, the same definition in Whyte et al., Nature 2012. We observed tiny changes for the insulation scores of the active_CTCF-bound, active_CTCF-less, silent_CTCF-bound, and silent_CTCF-less promoters in the revised Figure 6d. We also subgrouped the CTCF-less promoters into high/medium/low groups based on GRO-Seq signals for the genes. The analyses in the revised Figure 6e show that there are no or few differences in the changes in insulation scores for high/medium/low expressed promoters. Taken together, our results suggest that there are no or few transcription-associated effects on promoter-mediated chromatin interactions with the system that we investigated.*

*For revision, we added the sentences, “To further investigate the roles of Pol II in promoter-associated interactions, we performed insulation score analyses for different groups of promoters. We observed relatively few or no changes in the insulation scores of the active_CTCF-bound, active_no CTCF, silent_CTCF-bound, and silent_ no CTCF promoters in the Pol II depletion H3K27ac HiChIP and Ocean-C data sets (Figs. 6d, S6d). We then created high/medium/low subgroups of interactions-associated with no CTCF promoters based on gene expression with GRO-Seq data. The analyses also showed that there were no or few differences in the changes in the insulation scores under untreated or Pol II degron conditions (Figs. 6e, S6e).” to the revised manuscript P13/line26.*

3. Figure 3: promoter loop comparison

There is plenty of evidence that active promoters engage in long-range interactions (Schoenfelder et al., 2015; Bonev et al., 2017) but these interactions frequently appear in pairs and not in clusters (Olivares-Chauvet et al., Nature 2016). The authors should look at aggregate contact strength enrichment (PE-scan/APA, etc) between pairs of promoters of high/lowly expressed genes (based on GRO-seq for example); bound/not bound by CTCF and across multiple distances (intra vs interTAD; <2MB vs >2MB). Further to these, the analysis for the H3K27ac HiChIP and the Ocean-C should also quantify interactions strength at pairs of promoters, not just based on loop identification (based on Figure5A even loops classified as no/no still have weak CTCF binding). Analogously, for Pol I and Pol III, they should focus on genes known to be regulated by these polymerases (ribosomal, tRNAs, etc) in addition to the global comparison.

*Response: As the reviewer suggested, we have examined the paired-end spatial chromatin analysis (PE-SCAn, Elzo de Wit et al., Nature 2013, see Methods section Meta feature analysis) between pairs of promoters ranked by gene expression (based on GRO-seq) in the revised Figure 6b and S6b, and PE-SCAn plots show no or few differences with our Pol II depletion HiChIP and Ocean-C data sets. We also performed similar analyses based on bound/not bound by CTCF and ranked by distances in the revised Figure 6a, 6c, S6a, S6c. The results consistently show no or very few differences between +/- auxin conditions. In summary, PE-SCAn analysis with higher resolution (10 kb bins) HiChIP and Ocean-C data sets also revealed that Pol II protein depletion causes small-scale changes for the promoter-promoter interactions with our Pol II (Rpb1) degradation system in the mESCs.*

*For revision, we add the sentences, “Multiple studies have shown that active promoters engage in interactions over a long range, but the roles of Pol II proteins in these promoter pairs remain unclear. We performed paired-end spatial chromatin analysis (PE-SCAn) among different subgroups with our high-resolution H3K27ac HiChIP and Ocean-C data sets on interactions after Pol II degradation. The PE-SCAn plots of pairs of promoters across multiple distances (intra-TAD<2 MB, long-range>2 MB) (Figs. 6a, S6a) for a different expression levels (high/medium/low) (Figs. 6b, S6b), with CTCF binding and without CTCF binding (Figs. 6c, S6c), collectively showed that the distance and expression level modestly affected the active transcription-mediated promoter-promoter interactions in a CTCF-independent manner.” to the revised manuscript P13/line16.*

*We also focused on genes known to be regulated by Pol I, Pol II, and Pol III (ribosomal RNA, mRNA, and tRNA) in addition to the cluster comparison, as suggested by the reviewer. The 45s rRNA locus does not show obvious changes after Pol I depletion, while it appears to have a slight decrease for the chromatin interactions in mRNA and tRNA loci after the depletions of Pol II and Pol III, respectively.*

*For revision, we add the sentences, “The chromatin interactions of different genes that are known to be regulated by different RNA polymerases were also investigated. We found that the 45s rRNA locus did not obviously change after Pol I depletion, although relatively modest effects were found on the chromatin interactions in the mRNA and tRNA loci after the depletion of Pol II and Pol III (Fig. 3f-h), respectively.” to the revised manuscript P9/line22.*

4. Figure 6: effect on cohesin/CTCF vs global compaction

After 6hr the authors report a decrease in accessibility and cohesin binding across promoters, enhancers and CTCF sites. Is that due to a general chromatin compaction or a specific effect at CTCF/cohesin sites? One can potentially address that by comparing the accessibility at CTCF/cohesin bound vs not bound ATAC-peaks (promoters vs distal elements separately) or using Hi-C data (cis-decay profile in A- vs B- TADs).

*Response: As the reviewer suggested, we added the comparison of the accessibility at CTCF/Cohesin bound vs. not bound ATAC-peaks (promoters vs. distal elements separately) in +/- auxin conditions. The results in the revised Figure S7a show no difference for the ATAC-Seq peaks bound with or without CTCF/Cohesin in both promoters and distal elements, indicating general chromatin compaction after Pol II depletion for 6 hr.*

*For revision, we add the sentences, “The ATAC-Seq peaks bound with or without CTCF/Cohesin in both promoters and distal elements showed no obvious differences (Fig. S7a), indicating general chromatin compaction after 6 hr of Pol II depletion.” to the revised manuscript P14/line25.*

5. Effect on gene loops

Several studies based on micro-C (Hsieh et al., biorxiv) or Hi-C (Boxer et al., Molecular Cell 2020) suggest that particular types of loops between promoters and the gene body of highly transcribed genes are associated with transcription and that inhibition of transcription leads to their disruption (Hsieh et al., biorxiv). The authors should also examine these types of loops upon Pol II degradation with their high-resolution HiChiP, as this could be important and novel conceptual finding.

*Response: Loops between promoters and the gene body of highly transcribed genes indicate stripe signals in C-types of chromatin structure data sets. We performed the same stripe analyses (used in Hsieh et al., Molecular Cell 2020) with our H3K27ac HiChIP data sets at enhancer, promoter, and CTCF insulator regions. The results in the revised Figure S6f indicate that there are few or no differences of these stripe signals, suggesting Pol II proteins do not predominantly involve in mediating the loops between promoters and the gene bodies.*

*For revision, we add the sentences, “Loops between promoters and the gene bodies of highly transcribed genes indicated stripe signals in C-types of data on chromatin structures. We performed stripe analyses with our H3K27ac HiChIP data sets at enhancer, promoter, and CTCF insulator regions. The results showed that there were relatively few or no differences in these stripe signals.” to the revised manuscript P14/line5.*

Minor

It is very surprising to see more than 3000 peaks for Pol I, can this be cross-validated (for example comparing it with published untagged Pol I ChIP). Analogous for Pol III.

*Response: The ChIP-seq peaks obtained in the original submission were defined by overlapping the peaks from two replicates of the same RNAP ChIP-Seq samples by at least 1 bp. This definition is relative less stringent. To reduce the number of false-positive peaks, we measured the IDR (version 2.0.3) on peak calls from each RNAP set of replicates. IDR analysis is widely used by the ENCODE projects and is part of their ChIP-seq guidelines and standards for replicated experiments. This approach compares a pair of ranked lists of peaks and assigns values that reflect its reproducibility. We performed IDR analysis in each group with the MACS2 peak score as input, and then filtered all peaks that above the threshold of 0.05. The high-confidence reproducible peaks excluding the ENCODE blacklisted regions were used for further analysis. By IDR analyses, we got 605 peaks for Pol I ChIP-Seq data, 1845 peaks for Pol III ChIP-Seq data in the mES cells.*

*We also used the same pipeline to process the previously published untagged Pol I (POLR1A/RPA194) ChIP-Seq from mouse NIH3T3 cells (Sanij et al., Genome Research, 2015), and found their data set called 1409 peaks. The overlap analyses indicated that 23.5% of our Pol I peaks (142/605) were also detected in their NIH3T3 Pol I ChIP-Seq dataset. We noticed that Sanij et al. sequencing around 105 million 36 nt single-end reads while we were sequencing around 328 million 150 nt pair-end reads. Additionally, the low overlapping rate may also due to the use of different cell lines and antibodies.*

*We also checked the number of Pol III dataset with the published untagged Pol III ChIP-Seq (Canella et al., Genome Research, 2012), which are processed the same as ours. We found their data set called 1536 peaks. The overlap analyses indicated that 10% of our Pol III peaks (182/1845) were also detected in their mouse liver Pol III ChIP-Seq data set. By carefully investigating, we found that they were only sequencing 56.7 million 36 nt single-end reads, while we were sequencing 256.6 million 150 nt pair-end reads for Pol III ChIP-Seq. We also used different antibodies for the Pol III ChIP-Seq. These may be the reasons that we have a small number of overlapped Pol III peaks. On the other side, these differences may also be due to the differences of cell types, as the previous findings show that the cell type-specific Pol III binding and tRNA expression (Barski et al., Nature structural & molecular biology, 2010; Dittmar et al., PLoS Genetics, 2016).*

The Hi-C datasets seem to have an abnormally high trans/cis ratio, is that typical for BAT Hi-C?

*Response: The ratio of cis- and trans-unique pair-end reads, which is a critical feature for the signal-to-noise ratio, was 3.58±1.39 for BAT-Hi-C (average ratio for 22 libraries generated in this study), 2.10 ± 0.98 for in situ Hi-C (Rao et al., Cell, 2014), and 3.85 ± 0.18 for HiChIP (Mumbach et al., Nature Methods, 2016). Indeed, the cis/trans ratio for Pol II_IAA_6h_rep1 and rep2 for the 0 hr and 6 hr batch are 1.52 and 1.7, which is a little bit abnormal for Hi-C, but this is not typical for our BAT Hi-C. The reason for that is these two libraries were generated at the very early stage of the development of our BAT Hi-C. The BAT Hi-C protocol is not that optimized compared with our published one (Huang et al., Methods, 2020). Fortunately, our time course Hi-C data sets for the 0 hr, 1 hr, and 6 hr batches also include Pol II_IAA_6 hr conditions (cis/trans ratios are 3.64 and 3.69), the cis/trans ratios of these two seem much better. Both batches of the datasets consistently show that Pol II protein is dispensable for large-scale chromatin structures but contributes to local, small-scale chromatin structures. Additionally, we also checked the cis/trans of Hi-C datasets generated from Drs. Leonid Mirny and Job Dekker (Falk et al., Nature 2019), Erez Lieberman Aiden (Rao et al., Cell 2014), Bing Ren (Kubo et al., bioRxiv 2020), Giacomo Cavalli (Bonev B et al., Cell 2017), Rafael Casellas (Vian et al., Cell 2018) and Wei Xie’s (Du et al., Nature 2017) labs, and found the cis/trans of their Hi-C data sets range from 1.25 to 9.15. Together, we feel confident that the Hi-C data sets generated by our BAT Hi-C are reliable.*

*For revision, we added the sentences “The ratio of cis- and trans-unique pair-end reads, which is a critical feature for the signal-to-noise ratio, was 3.58±1.39 for BAT-Hi-C (average ratio for 22 libraries generated in this study).” in the revised manuscript method section, and the cis/trans ratio of our libraries to the revised Table S2.*

Clustering at figure S2D should be done using individual replicates to increase robustness

*Response: We redo the clustering analyses with individual replicates to increase robustness in the revised Figure S2d, as suggested by the reviewer. All replicates show no or few changes in TAD and compartment structures, with relatively modest variations between the replicates. Additionally, detailed per replicate information for generated Hi-C data has been provided in Table S1.*

TADs defined by arrowhead tend to be mostly loop domains and are fewer than described in other papers from the same cell type. The authors should verify those finding using other approaches (insulation score, etc.) or publically available TAD coordinates (Bonev et al., Cell 2017)

*Response: We first compared TAD domains defined by arrowhead with two popular TAD calling algorithms, such as insulation score (Crane, E. et al., Nature 2015) and Directionality Index (DI) (Dixon et al., Nature 2012). The arrowhead indeed calls fewer TADs, but the number is not so different for the other approaches (see the bar graph below. This figure is not included in the revised manuscript because of the space limitation). On the other side, we also used insulation scores analyses and publically available TAD coordinates from Bonev et al., Cell 2017 in the revised manuscript, and consistently show RNA polymerase transcription modestly contributes to the local, small-scale 3D chromatin structures in mESCs.*

The data in Figure S2F is important but it is unclear which degron is referred to. Data for all of the +/auxin combinations should be shown here. Also S2F is not observed/expected ratios as in the text.

*Response: The original Figure S2f refers to the Pol II degron, and we have labeled it clear in the revised figure. We also performed similar analyses for Pol I, Pol III, and mitotic exit Hi-C data sets and found no changes between +/- auxin conditions but did not show them in the original submission. We now add them to the revised Figure S2f. The reviewer is right that the original Figure S2f is not observed/expected ratios, as in the text. This analysis was performed with relative contact frequency instead of observed/expected ratios.*

*For revision, we modified the text to “the observed/expected chromatin interactions for the TAD structures did not change notably after RNA polymerase degradation (Fig. 2a-c, right panel), and the relative chromatin interaction frequencies were almost the same for different distances (Fig. S2g).” in the revised manuscript P8/line5.*

Figure 4D: please add statistics: is this enrichment higher/lower than expected based on the frequency and length of the genomic feature?

*Response: In processing PLAC-seq data, we adopted Origami (Weintraub et al., Cell 2017), a statistical method to identify high-confidence interactions that are likely to represent structured chromatin contacts. First, Origami estimates a probability score for each putative interaction by modeling the relationship between the number of PETs observed to support each interaction, linear genomic distance between interaction anchors, and read depth at the interaction anchors. Then, it uses a semi-Bayesian two-component mixture model to estimate the probability that a putative interaction corresponds to one of two groups: structured chromatin contacts (Group 1) or non-structured chromatin contacts and technical artifacts (Group 0). In the observed/expected analysis, the probability score is determined by iteratively estimating the group identity of each putative interaction, which reflects the probability that the interaction results come from Group 1, then a hypergeometric p-value is calculated based on the number of overlapping features in the data as compared to the number of overlapping features in the background dataset (follows the binomial distribution) with 10000 permutations. High-confidence interactions are then identified as the subset of putative interactions that are likely to represent structured chromatin contacts by requiring a final origami score of at least 0.9 and a minimum PET count of 5. This information has now been added to the PLAC-seq analysis section in the revised manuscript method section.*

Contact enrichment (obs/exp) should be also quantified at promoter-promoter and promoter-enhancer loops, not just mean distance.

*Response: We added contact enrichment (obs/exp) quantification at promoter-promoter (average PET number 11.16, Hypergeometric p-value 3.563683e-04) and promoter-enhancer loops (average PET number 15.28, Hypergeometric p-value 6.477318e-06) to the Figure 4D legend.*

*For revision, we added the sentence, “Contact enrichment (obs/exp) for promoter-promoter (average PET number 11.16, hypergeometric p-value 3.563683e-04) and promoter-enhancer loops (average PET number 15.28, hypergeometric p-value 6.477318e-06) were quantified.” to the revised manuscript the Figure 4d legend.*

p14/ lines 48-51: unclear what is meant with this sentence.

*Response: the sentence in p14/ lines 48-51 is, “We then selected Pol II bound loops in the absence of Cohesin and found CTCF binding almost invisible at these loops.” We mean here that the loops with bound Pol II but without Cohesin showed very little CTCF binding.*

*For revision, we replaced the sentence with “We found that the loops with bound Pol II but without Cohesin showed very little CTCF binding.” in the revised manuscript P12/line13.*

**Second round of review**

**Reviewer 1**

Overall the authors have made solid improvements to the manuscript, including by the addition of analyses that compare the degredon cell lines with wild type, to show that the tagged proteins do not have a notable effect on the polymerase, and also the inclusion of analyses that show CTCF independence. Overall these make the assertions more robust, which is especially required when showing that Pol has small or no effect for certain aspects. Also, the improved language makes the manuscript somewhat easier to read and makes the take-home messages clearer.

I only have some minor textual comments on the manuscript:

Abstract

In "(for the first time this has been shown)" remove "for".

Consider replacing "confusing" with a more technical term, like seemingly "inconsistent" or "contradictory".

Background

In the sentence "Although the structure and function of RNA polymerases have been studied for 50 years [34], the roles of Pol I and Pol III in 3D chromatin organization, in particular, have been insufficiently investigated."

I suggest changing "insufficiently investigated." to "poorly investigated compared to Pol II". - It is not oblivious what any sufficiency criterion would be.

Overall, I suggest rephrasing the term "selected regions" to "small, specific regions" (or similar) as "selected" might suggest the authors' choice.

Results

First paragraph, second sentence needs a further adjustment. I suggest something like:

"however they either do no have the capacity to distinguish different RNA polymerases or are specific to a single RNA polymerase (such as alpha-amanitin) and only work at high concentrations, thus requiring a long treatment duration[35, 36].

I suggest changing the latter part of "polyA RNA-Seq did not changed dramatically" to "was not changed dramatically"

In the "A/B Compartments" section the sentence ending "conformational features such as DNA loops efficiently" repeats "efficiently".

I would pluralise "first delineated using Eigenvector[s]".

The various occurrences of "45s rRNA" should be "45S rRNA"

It is not clear to me how the following sentence relates to what is shown in Figure S3: "The histogram shows that most of the loop interaction strength does not change notably after Pol II degradation for the loops identified by both algorithms with the Hi-C, HiChIP, and Ocean-C data sets (Fig. S3)." The histograms for degredon and untreated samples seem fairly different in places...

Change "A loop-length analyses" to use singular "analysis".

In the paragraph before the "Immediate depletion of Pol II..." a third reason for the lack of changes to the stripe signal could be that these interactions are specific and setup by the TFs etc. that are present, and not by the general transcriptional process.

Discussion

In "By inhibiting transcription for extended periods, many potential secondary effects may emerged" change "emerged" to "emerge".

I would remove "not" from "but not for the short-term treatment did not have this effect (Fig. 7b-c)" to give "but the short-term treatment did not have this effect (Fig. 7b-c)"

Figures

In Fig 4D I suggest changing "loop number" to "loop count".

In the legend for Figures 4D and 4E what are the cellular conditions/type?

**Reviewer 2**

The authors have done excellent job with the revision and have addressed all my initial comments to my satisfaction. I now fully recommend this manuscript for publication and I'm looking forward to see its impact on the community.

**Authors Response**

**Point-by-point responses to the reviewers’ comments:**

Reviewer #1: I only have some minor textual comments on the manuscript:

Abstract

In "(for the first time this has been shown)" remove "for".

Consider replacing "confusing" with a more technical term, like seemingly "inconsistent" or "contradictory".

*Response: we used the edited abstract in the revised manuscript.*

Background

In the sentence "Although the structure and function of RNA polymerases have been studied for 50 years [34], the roles of Pol I and Pol III in 3D chromatin organization, in particular, have been insufficiently investigated."

I suggest changing "insufficiently investigated." to "poorly investigated compared to Pol II". - It is not oblivious what any sufficiency criterion would be.

*Response: we changed the words as the reviewer suggested in the revised manuscript page 4, line 17.*

Overall, I suggest rephrasing the term "selected regions" to "small, specific regions" (or similar) as "selected" might suggest the authors' choice.

*Response: we changed "selected regions" to "small, specific regions" across the revised manuscript.*

Results

First paragraph, second sentence needs a further adjustment. I suggest something like:

"however they either do no have the capacity to distinguish different RNA polymerases or are specific to a single RNA polymerase (such as alpha-amanitin) and only work at high concentrations, thus requiring a long treatment duration[35, 36].

*Response: we changed the sentence as the reviewer suggested in the revised manuscript page 5, line 12.*

I suggest changing the latter part of "polyA RNA-Seq did not changed dramatically" to "was not changed dramatically"

*Response: we changed the words as the reviewer suggested in the revised manuscript page 5, line 30.*

In the "A/B Compartments" section the sentence ending "conformational features such as DNA loops efficiently" repeats "efficiently".

*Response: we deleted the repeat “efficiently” in the revised manuscript page 7, line 2.*

I would pluralise "first delineated using Eigenvector[s]".

*Response: we used “Eigenvectors” as the reviewer suggested in the revised manuscript.*

The various occurrences of "45s rRNA" should be "45S rRNA"

*Response: we used “45S rRNA” as the reviewer suggested in the revised manuscript.*

It is not clear to me how the following sentence relates to what is shown in Figure S3: "The histogram shows that most of the loop interaction strength does not change notably after Pol II degradation for the loops identified by both algorithms with the Hi-C, HiChIP, and Ocean-C data sets (Fig. S3)." The histograms for degredon and untreated samples seem fairly different in places...

*Response: we changed the sentence to “The histogram shows that most of the loop interaction strength seems to be affected after Pol II degradation for the loops identified by both algorithms with the Hi-C, HiChIP, and Ocean-C data sets” in the revised manuscript page 10, lane 15.*

Change "A loop-length analyses" to use singular "analysis".

*Response: we changed to “analysis” as the reviewer suggested in the revised manuscript page 10, line 25.*

In the paragraph before the "Immediate depletion of Pol II..." a third reason for the lack of changes to the stripe signal could be that these interactions are specific and setup by the TFs etc. that are present, and not by the general transcriptional process.

*Response: we added the sentence “or 3) these interactions are specific and setup by the transcription factors etc. that are present, and not by the general transcriptional process” as the reviewer suggested in the revised manuscript page 14, line 21.*

Discussion

In "By inhibiting transcription for extended periods, many potential secondary effects may emerged" change "emerged" to "emerge".

*Response: we changed to “emerge” as the reviewer suggested in the revised manuscript page 15, line 9.*

I would remove "not" from "but not for the short-term treatment did not have this effect (Fig. 7b-c)" to give "but the short-term treatment did not have this effect (Fig. 7b-c)"

*Response: we deleted “not” as the reviewer suggested in the revised manuscript page 16, line 6.*

Figures

In Fig 4D I suggest changing "loop number" to "loop count".

*Response: we changed to “loop count” as the reviewer suggested.*

In the legend for Figures 4D and 4E what are the cellular conditions/type?

*Response: they are wild type ES cells, we added the information in the revised figure legend in page 31, line 17.*
